# Two classes of intrahepatic cholangiocarcinoma defined by relative abundance of mutations and copy number alterations

**DOI:** 10.18632/oncotarget.8183

**Published:** 2016-03-18

**Authors:** Young-Ho Kim, Eun-Kyung Hong, Sun-Young Kong, Sung-Sik Han, Seoung-Hoon Kim, Je-Keun Rhee, Soo-Kyung Hwang, Sang-Jae Park, Tae-Min Kim

**Affiliations:** ^1^ Translational Epidemiology Research Branch, Research Institute and Hospital, National Cancer Center, Goyang, Republic of Korea; ^2^ Center for Liver Cancer and Hospital, National Cancer Center, Goyang, Republic of Korea; ^3^ Department of Laboratory Medicine, Diagnostic Oncology Center and Hospital, National Cancer Center, Goyang, Republic of Korea; ^4^ Department of System Cancer Science, Graduate School of Cancer Science and Policy, National Cancer Center, Goyang, Republic of Korea; ^5^ Department of Medical Informatics, The Catholic University of Korea, Seoul, Republic of Korea; ^6^ Cancer Research Institute, College of Medicine, The Catholic University of Korea, Seoul, Republic of Korea

**Keywords:** cholangiocarcinoma, exome sequencing, transcriptome, somatic mutations

## Abstract

Intrahepatic cholangiocarcinoma (ICC) is a biliary tree-origin epithelial malignancy in liver with unfavorable clinical outcomes. Systematic genome analyses may advance our understanding of ICC pathogenesis also improving current diagnostic and therapeutic modalities. In this study, we analyzed 17 ICC tumor-vs-matched normal pairs using either whole-exome (*n =* 7), transcriptome sequencing (*n* = 7) or both platforms (*n* = 3). For somatic mutations, we identified recurrent mutations of previously reported genes such as *KRAS, TP53, APC* as well as epigenetic regulators and those of TGFβ signaling pathway. According to the abundance of somatic mutations and DNA copy number alterations (CNA), ten ICC exome cases were distinguished into two classes as those primarily driven by either somatic mutations (M class) or CNAs (C class). Compared to M class ICCs (92–147 somatic mutations; *n* = 5) with a relative deficit of CNAs, C class ICCs (54–84 mutations; *n* = 5) harbor recurrent focal CNAs including deletions involving *CDKN2A, ROBO1, ROBO2*, *RUNX3*, and *SMAD4*. We also show that transcriptome sequencing can be used for expression-based ICC categorization but the somatic mutation calling from the transcriptome can be heavily influenced by the gene expression level and potentially, by posttranscriptional modification such as nonsense mediated decay. Along with a substantial level of mutational heterogeneity of ICC genomes, our study reveals previously unrecognized two ICC classes defined by relative abundance of somatic mutations over CNAs or vice versa, which should be considered in the selection of genotyping platforms and sensitive screening of targets for ICC therapeutics.

## INTRODUCTION

Cholangiocarcinomas (CCA) are epithelial tumors arising from biliary trees with features of cholangiocyte differentitation [1]. CCA accounts for about 3% of total gastrointestinal malignancies (10 to 15% of all primary hepatobiliary cancers) with an increasing incidence over the last decade [2]. Patients with CCA have unfavorable prognosis, i.e., median survival of 24 months after the diagnosis. The surgical resection in early stages remains the only curative option for CCA, which is achieved for only 30% of the patients [3]. The current standard chemoregimen for CCA - the combined use of gemcitabine and cisplatin - has a limited improvement on the survival compared to the use of gemcitabine alone (e.g., 11.7-*vs*-8.1 months) [4]. According to the location in the biliary tree, CCAs are categorized into intrahepatic cholangiocarcinoma (ICC) as those arising in the hepatic parenchyma and extrahepatic CCA such as perihilar and distal CCAs. ICC cases comprise about 10% of the total CCA cases and they are often diagnosed at a later stage due to anatomic locations [5]. Some of the effective treatment options such as the neoadjuvant chemoradiation with the liver transplantation can be considered only for a limited subtype such as prehilar CCA highlighting a pressing need to advance targeted therapeutics for ICC [6].

Genome-wide studies have revealed potential oncogenic drivers of CCA and ICC and their recurrent nature in given cohorts. For example, whole-exome sequencing (WES) of fifteen CCA has revealed recurrent somatic mutations of *KRAS*, *TP53* and *SMAD4* [7, 8]. Along with 32 ICC WES study [9], those studies also revealed novel mutations, such as those arising in chromatin remodeling genes (e.g., *BAP1* and *ARID1A*) and metabolic genes (i.e., *IDH1* and *IDH2*). The examination of mutations across various gastrointestinal tumors revealed that *IDH1* or *IDH2* mutations are specific to ICC and they may serve as druggable targets [10]. The frequent mutations on protein tyrosine phosphatases including *PTPN3* in ICC genomes have been also recently reported [11]. The druggable targets that have been reported in ICC genomes are summarized elsewhere [12]. But it is still largely unknown as to the extent of mutational heterogeneity and the potential benefit of exome- or transcriptome-wide mutation screening of ICC in respect to the targeted therapeutics.

In this study, we performed WES and transcriptome sequencing (RNA-seq) to examine somatic mutations, read depth-based copy number alterations (CNAs) as well as gene expression for 17 ICC cases. First, we discuss WES-based identification of somatic mutations and CNAs, also demonstrating that ICC cases can be classified into two major molecular classes that are primarily driven by somatic mutations or CNAs. Then, we will discuss about the RNA-seq based somatic variants calling with additional findings on ICC transcriptomes. Our integrative analyses revealed previously unrecognized insights that may improve our understanding into the ICC pathogenesis as well as to advance current ICC therapeutics.

## RESULTS

### The landscape of somatic variants of ICC

**Figure 1:**
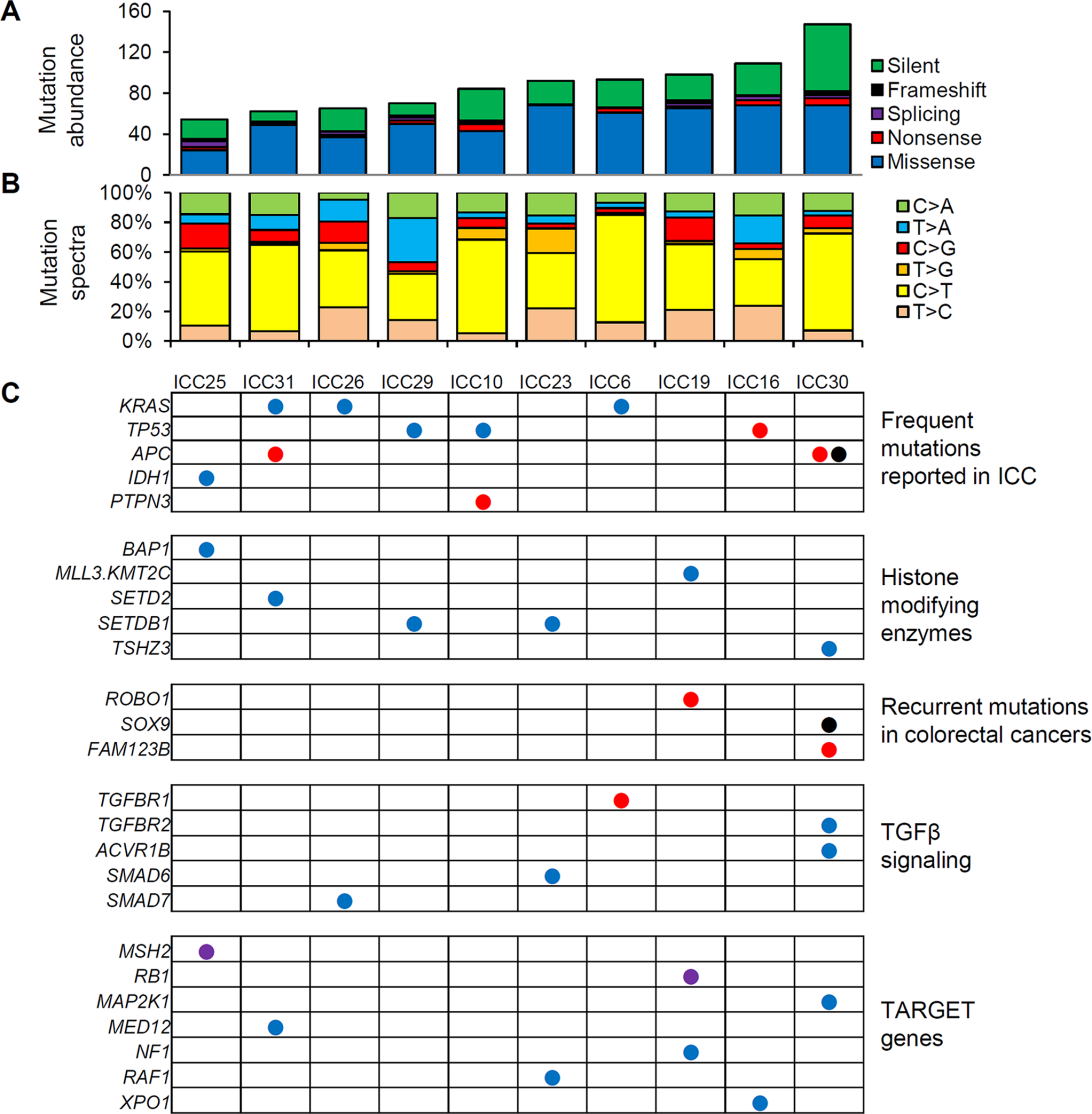
WES-based somatic mutation landscape of ICC (**A**) Ten ICC genomes are sorted in order of the mutational abundance. Somatic mutations are classified into five functional categories as reported by ANNOVAR with respective colors. (**B**) The six mutation spectra are shown with the dominance of C:G to T:A transitions in ICC genomes. (**C**) Nonsilent mutations on cancer-related genes are illustrated. The genes are categorized into five major categories. As cancer-related genes not previously reported to be recurrent in ICC, we used TARGET (tumor alterations relevant for genomics-driven therapy) database. Blue, red, purple, and black circles represent missense, nonsense, splicing mutations and frameshift indels, respectively.

The clinicopathological information of 17 ICC patients examined in this study is available in Table 1. We first performed WES of tumor and patient-matched adjacent normal genomic DNA to identify somatic point mutations (single nucleotide variants) and short indel for 10 ICC cases. As a result, we identified a total of 874 somatic variants in 10 ICC cases (54 to 147 variants per case; median of 88 variants) (Figure 1A). The full list of somatic variants is available in Supplementary Table S1. The sequencing depth and target coverage of WES is shown in Supplementary Table S2. Somatic mutations also showed the dominance of C-to-T transition (31.2% to 72.4% of six mutation spectra across the cases) as previously reported (Figure 1B) [7, 8].

**Table 1:** Clinicopathological information of ICC patients

Case	WES	RNA-Seq	Gender	Age (yrs; at diagnosis)	HBV Ag	HCV Ag	Hepatolithiasis	CA19–9 (U/m)	Tumor Differentiation	Tumor size (cm)	TNM stage	Vital Status	Follow-up months
ICC3		Yes	M	60	No	No	No	9.8	M	2.5	T1N0M0	Alive	19
ICC4		Yes	F	71	Yes	No	No	7	M	3.9	T1N0M0	Alive	18
ICC5		Yes	M	48	No	No	No	625	P	8	T3N1M0	Alive	17
ICC6	Yes	Yes	M	65	No	No	No	1327	P	7.5	T3N1M0	Death	7
ICC8		Yes	M	66	No	No	No	ND	P	6	T1N0M0	Death	9
ICC10	Yes	Yes	M	63	No	No	No	ND	P	9.8	T1N0M0	Alive	13
ICC11		Yes	M	66	No	No	No	14.1	P	4.8	T2bN1M0	Alive	12
ICC14		Yes	F	69	No	No	Yes	17630	P	10.5	T3N1M0	Death	4
ICC15		Yes	F	58	No	No	No	12.5	P	4.2	T2aN0M0	Alive	10
ICC16	Yes	Yes	F	62	Yes	No	No	18	ND	2.7	T4N0M0	Alive	8
ICC19	Yes		F	63	Yes	No	No	ND	P	2.5	T3bN0M0	Death	8
ICC23	Yes		M	49	Yes	No	No	ND	P	2.2	T1N0M0	Alive	74
ICC25	Yes		M	64	No	No	No	310	P	2.2	T2N0M0	Death	52
ICC26	Yes		M	57	Yes	No	No	ND	P	3.4	T4N1M0	Alive	67
ICC29	Yes		M	70	No	No	No	15.3	ND	3.6	T1N0M0	Death	9
ICC30	Yes		M	78	No	No	No	86	M	8	T1N0M0	Death	20
ICC31	Yes		M	61	No	No	No	2297	P	5	T4N1M0	Alive	7

ND represents non-determined in the corresponding case. Tumor differentiation are poor- (P), moderate- (M) and well-differentiated (W). Follow-up months represent the months to death in case of deceased patients.

Figure 1C illustrates the mutations previously reported as relevant in ICC or in other types of cancers. First, *KRAS* and *TP53* mutations were the most frequent targets of somatic mutations in ICC (30% of cases). All three missense *KRAS* mutations occurred at known hotspots of amino acid residues of position 12, 13 and 61 (G12D, G13D, Q61L in ICC26, ICC6, ICC41, respectively) as likely cancer drivers of three ICC cases. Three nonsilent *TP53* mutations include one nonsense mutation as an apparent loss-of-function event. All three *APC* mutations are loss-of-function events (two nonsense mutations and one frameshifting indel) and two of them were observed in one case (ICC30) suggestive of bialleleic inactivating events. Among the non-recurrent but ICC-relevant singleton mutations, a missense mutation was observed in *IDH1* at well-known hotspot of substrate binding (R132L) [13]. One nonsense *PTPN3* mutation was also observed as recently identified recurrent mutation targets on ICC [11]. Among the mutations that may affect the epigenetic regulation, we observed one *BAP1* missense mutation as well as additional missense mutations on *MLL3* (*KMT2C*), *SETD2*, *SETDB1*, and *TSHZ2* suggesting that the histone modification may be largely perturbed by somatic mutations during ICC development. We observed a nonsense *ROBO1* mutation as a potential tumor suppressor gene reported in other gastrointestinal tumors [14]. Loss-of-function mutations frequently observed in colorectal cancers (one frameshift indel in *SOX9* and a nonsense mutation in *FAM123B*) were also found in ICC genomes [15]. We also found lines of evidence that TGFβ signaling may be frequently perturbed by somatic mutations including a nonsense *TGFBR1* mutation along with additional missense mutations on *TGFBR2* and inhibitory SMADs such as *SMAD6* and *SMAD7*. Among known cancer-related genes [16], we observed splicing mutations on *MSH2* and *RB1* as well as a number of missense mutations on *GNAS*, *MAP2K1*, *MED12*, and *NF1*, which requires further investigation for their oncogenic potential in ICC. For the validation, we performed Sanger sequencing for the 20 variants on 14 selected genes, i.e., five genes harboring frequent ICC mutations (*KRAS*, *TP53*, *APC*, *IDH1*, and *PTPN3*), six cancer-related genes in TARGET database (*RB1*, *MAP2K1*, *MED12*, *NF1*, *RAF1*, and *XPO1*) and selected genes in the remaining categories (*BAP1*, *ROBO1* and *SMAD7*) (Supplementary Figure S1). The presence of peaks consistent with minor mutant alleles were confirmed for all the single base substitutions as well as for one out-of-frame indel on *APC*. As candidates of novel biomarkers, we report the recurrent nonsilent mutations (i.e., 19 nonsilent mutations observed in more than one ICC genomes but not listed in Figure 1) in Supplementary Figure S2.

### Chromosomal CNAs of ICC genomes

**Figure 2:**
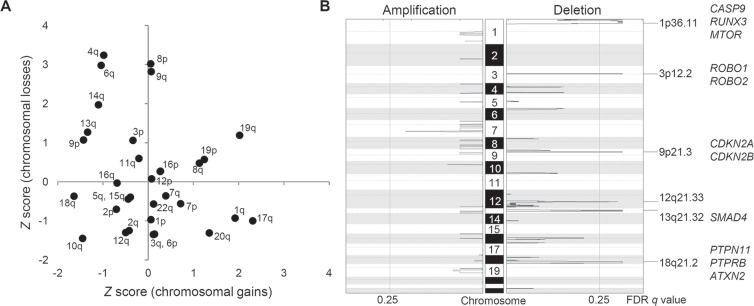
Recurrent arm-level and focal chromosomal CNAs in ICC (**A**) Chromosomal arms are shown with respect to the frequency of arm-level amplifications (X-axis) and deletions (Y-axis), respectively. As a frequency measure, *Z* score from GISTIC output is used. We report recurrent chromosomal arm gains and losses for those with *Z* score > 1. (**B**) Six focal deletions are shown as significantly (false discovery rate or FDR < 0.25) recurrent in ten ICC genomes. Selected cancer-related genes in focal peaks are shown at right.

For copy number profiling, we used log_2_-scaled and segmented read depth differences between the ICC tumor and matched normal WES data. First, broad chromosomal arm-level analyses using GISTIC algorithm [17] revealed frequent chromosomal losses of 3p, 4q, 6q, 8p, 9p/q, 13q, and 14q along with frequent chromosomal gains of 1q, 8q, 17q, 19p/q, and 20q (Figure 2A). These frequently altered chromosomal arms are largely concordant as previously reported by meta-analysis of ICC studies [18]. Second, GISTIC-based peak analyses identified additional focal and recurrent chromosomal deletions on 1p36, 3p12, 9p21, 12q21, 13q21, and 18q21 whereas no additional focal chromosomal amplification was identified (Figure 2B). Supporting our findings, a previous high-resolution study showed that focal chromosomal amplifications, unlike arm-level changes, are not as frequent as focal deletions in ICC genomes [29]. Thus, we considered GISTIC-based six focal deletions are potential drivers of ICC. The GISTIC output of six focal deletions are available in Supplementary Table S3.

### Two distinct ICC classes defined by the relative abundance of somatic mutations and CNA

**Figure 3:**
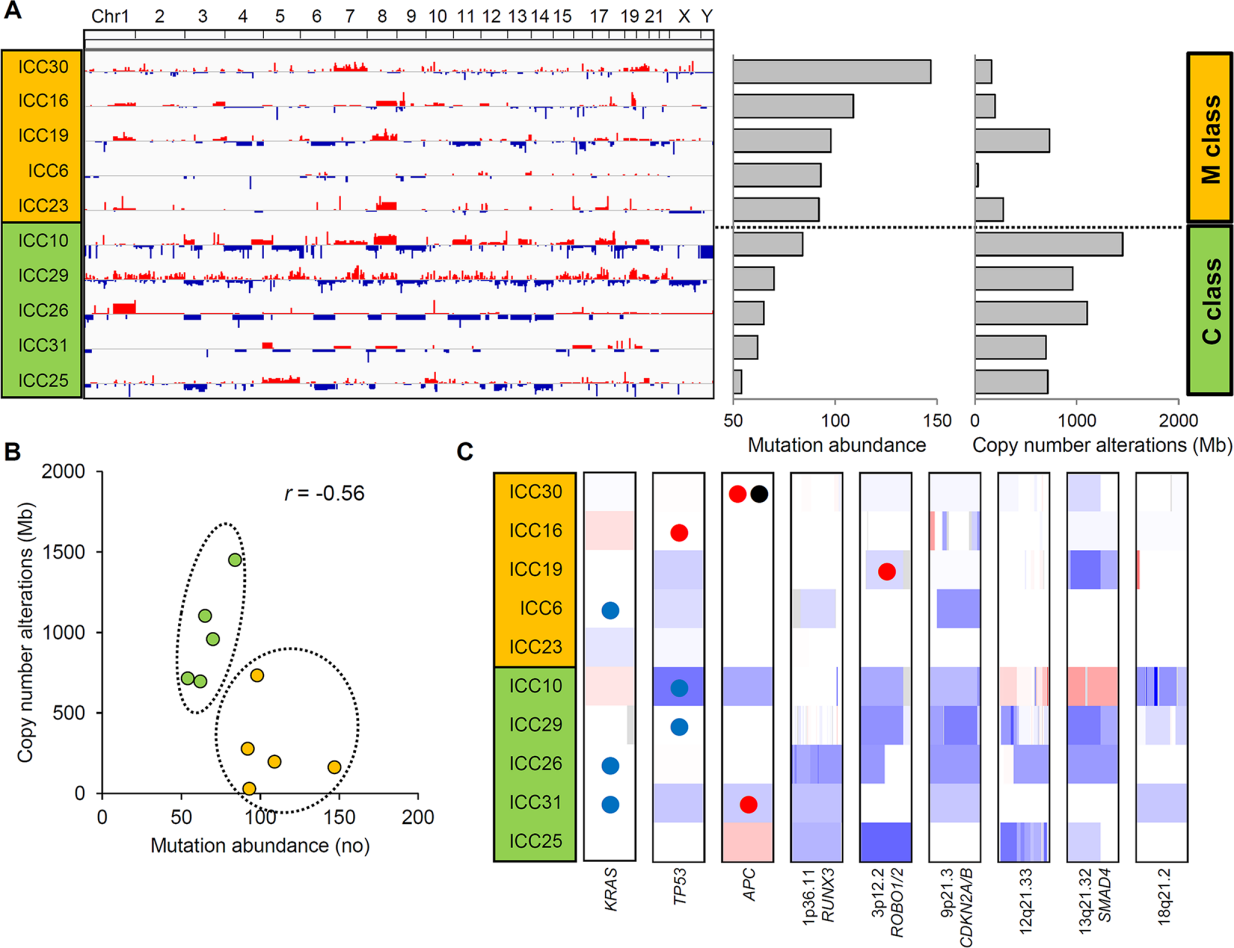
Two ICC classes defined by the abundance of somatic mutations and CNAs (**A**) Ten ICC cases are sorted in order of mutational abundance. The half of the ICC cases with more number of somatic mutations (above) show a relative deficit of CNAs compared to the half with less number of mutations (below). These two ICC classes are annotated as M and C class (orange and green), respectively. (**B**) A scatter plot shows that ICC cases can be distinguished into two classes with a negative correlation (*r* = −0.56) between the abundance of mutations and CNAs. (**C**) Along with three loci with recurrent somatic mutations (*KRAS*, *TP53* and *APC*), six focal and recurrent deletions identified by GISTIC are shown. One-Mb regions encompassing *KRAS*, *TP53* and *APC* along with focal deletions as defined by GISTIC are arbitrarily shown in the same-sized windows with red and blue representing chromosomal gains and losses, respectively. Colored circles represent the concurrent somatic mutations with blue, red and black representing missense, nonsense mutations and frameshift indels, respectively.

**Figure 4:**
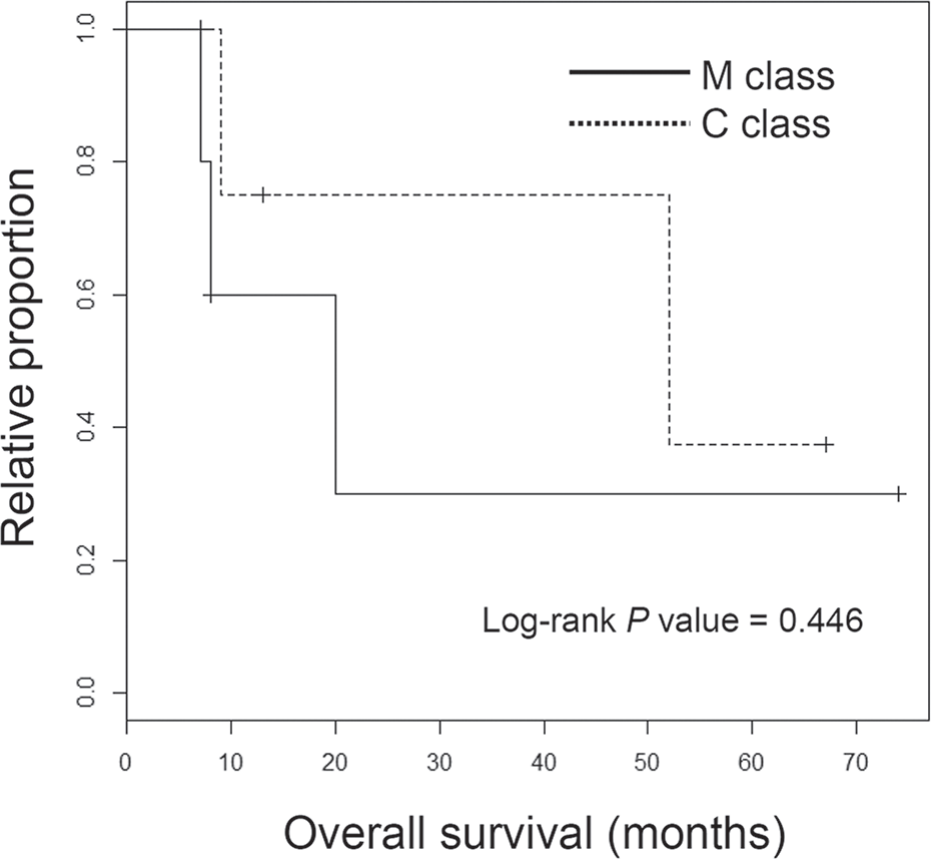
Survival analyses of ICC classes Kaplan-Meier survival plots are shown for M and C class ICC patients with the significance level (*P* value from log-rank test).

The genome-wide chromosomal heatmap of CNAs are shown in Figure 3A. Of note, when ten ICC genomes are sorted in order of mutation abundance, the majority of CNAs are observed in the cases with less number of somatic mutations (i.e., five ICC genomes with < 90 mutations per case) while the other five cases (> 90 mutations per case) show a relatively deficit of CNAs. This characteristic preference of ICC genomes to either somatic mutations or CNAs, can classify the cases into five M and C classes, as primarily driven by *m*utation and *c*opy number alterations, respectively. A substantial level of negative correlation (*r* = −0.568; *P* = 0.086) was also observed between the number and the genomic fraction of CNAs (Figure 3B). This correlation is largely attributed to chromosomal deletions (*r* = −0.684) rather than amplifications (*r* = −0.074). The copy number heatmaps corresponding to six focal deletions (Figure 2B) along with those of three loci with recurrent somatic mutations (*KRAS*, *TP53* and *APC*) are shown (Figure 3C). While the activating *KRAS* mutations appeared independent of CNAs, two potentially inactivating mutations (each of *TP53* and *APC* mutations) coincide with chromosomal deletions suggestive of biallelic inactivating events. In case of focal deletions, one at 1p36 harbors *RUNX3* that is frequently inactivated in ICC genomes by chromosomal deletion or promoter hypermethylation [20]. We also identified frequent focal deletions at 3p12 harboring *ROBO1* and *ROBO2*, one of which accompanies a nonsense mutation of *ROBO1*. Aberration of SLIT/ROBO signaling has been recently identified in other gastrointestinal tumors [14] suggestive of its potential oncogenic roles in ICC pathogenesis. In addition to *CDKN2A* and *CDKN2B* on 9p21, frequent deletions were observed on 13q21 harboring *SMAD4* whose inactivation appears to be entirely dependent on chromosomal deletions in our ICC cases. In addition, survival analyses revealed that M and C classes did not show significant difference in overall survival (log-rank *P* = 0.446; Figure 4). But survival plots of two classes show a segregation where M classes tend to have relatively shorter survival than C classes.

### Transcriptome sequencing of ICC

We further performed RNA-seq for ten ICC cases, three of which were also profiled by WES. Using a similar analysis pipeline designed for WES (see Materials and Methods), we identified a total of 465 somatic variants from RNA-seq (15–134 mutations per case; median of 39). The list of somatic variants called from RNA-seq and the sequencing information of RNA-seq are available in Supplementary Table S4 and Supplementary Table S5, respectively. In comparison with WES, we considered three cases both available for RNA-seq and WES (ICC6, ICC10 and ICC16). Among 289 somatic variants identified from WES in these three cases, only 65 variants (22.5%) were rediscovered by RNA-seq. This low concordance level is largely comparable to those reported for other tumor type (e.g., 36% for validated somatic mutations were expressed and detected in RNA-seq of breast cancers) [21]. Indeed, we observed that the median local coverage of WES mutations that were also found by RNA-seq or not, were 84X and 3X in RNA-seq, respectively. Thus, the WES mutations with low level of gene expression may be undetected by RNA-seq and this may also explain the low mutational abundance of RNA-seq compared to WES, i.e., median number of RNA-seq and WES mutations per case were 39 and 88, respectively.

**Figure 5:**
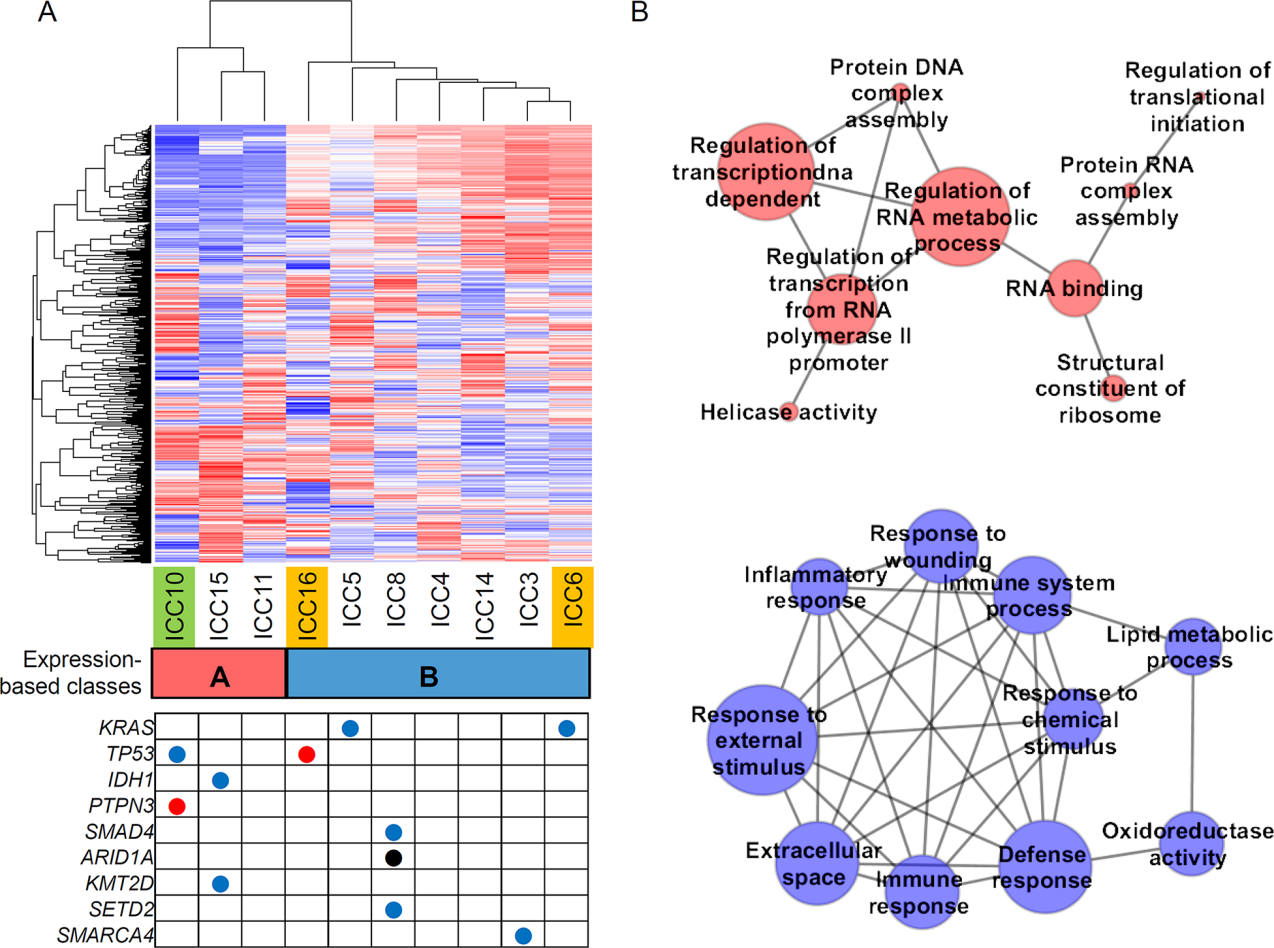
Expression-based ICC taxonomy (**A**) Hierarchical clustering of 1000 genes with variable expression segregates ten ICC expression profiles into two classes. Class A (*n* = 3; red) and class B (*n* = 7; blue) include one C class and two M class ICC cases (green and orange, respectively). Nine ICC-relevant somatic mutations called from the RNA-seq are shown in the table below. Red, blue and black represent the nonsense, missense mutations and frameshift indel, respectively. (**B**) Top ten enriched molecular functions identified by GSEA are shown in association networks. Red nodes are GO molecular terms whose gene members are relatively overexpresssed in class A compared to class B and blue for vice versa. The size of nodes corresponds to number of leading edge gene subsets of the corresponding GO terms. Edge represents the significant (Bonferroni corrected *P* < 0.05; Fisher's exact test) overlap of leading edge gene subsets between two nodes or molecular terms. One red node without any significant overlap with the remaining nodes is ignored.

Among the six potential driver mutations found in WES (ICC6, ICC10 and ICC16; Figure 1C), five mutations of *KRAS* (one missense), *PTPN3* (one nonsense), *TP53* (one missense and one nonsense), and *XPO1* (one missense) were also identified by RNA-seq. Of note, no mutant sequencing read was found in RNA-seq for one nonsense mutation on *TGFBR1* (Supplementary Figure S3). The absence of mutant reads in RNA-seq may be due to moderate coverage in RNA-seq (20X) and low MAF (5.1% in WES) of the corresponding mutation. Moreover, nonsense mediated decay may be also responsible for the absence of mutant RNA-seq reads [22], suggestive of additional source for the low sensitivity of RNA-seq based somatic mutation identification. Among the additional seven cases profiled by RNA-seq, we identified potential oncogenic drivers such as *KRAS* (G12D in ICC5), *IDH1* (R132C in ICC15) and *SMAD4* missense mutations (E134D in ICC8). For somatic mutations on epigenetic regulators, we found one frameshifting indel on *ARID1A* (ICC8) and missense mutations on *KMT2D* (*MLL2* in ICC15), *SETD2* (ICC8), *SMARCA4* (ICC3) (Figure 5A).

Hierarchical clustering of 1000 genes with top variable expression segregated the ten ICC gene expression profiles into two classes (class A and B with 3 and 7 ICC cases, respectively; Figure 5A). We further performed Gene Set Enrichment Analysis (GSEA) [23] to identify the GO (Gene Ontology) molecular terms enriched with genes showing relative over- or under-expression in class A compared to class B. The functional association maps (Figure 5B) show that top GO terms overexpressed in class A largely represent the DNA/RNA metabolism while those overexpressed in class B represent immune or inflammation functions. These functional categorization coincide with those previously reported molecular taxons of ICC based on gene expression (i.e. proliferation-vs-inflammation classes) [24]. Five genes (MBL2, AQP9, IL27, CCR1, and CCL24) were selected for the validation by quantitative PCR. The genes were selected among the leading edge gene subset of ‘Immune response’ gene category that showed the most significant up-regulation in class B (Supplementary Figure S4).

## DISCUSSION

ICC is a dismal disease with an increasing prevalence worldwide. Although conventional cytotoxic chemoregimen has shown a limited response rate in the treatment of ICC [25], it is expected that ICC patients will soon benefit from the targeted therapeutics based on the genomic profiling of individual cancer genomes. In this study, our WES and RNA-seq based analyses have revealed a number of important insights underlying ICC pathogenesis and targeted therapeutics against ICC. For example, we observed that ICC can be classified into two molecular classes that are relatively overrepresented with somatic mutations but depleted of CNAs (M class) or vice versa (C class). Although a similar notion has been recently reported across different tumor types [26], to our knowledge, this is the first demonstration of two distinct molecular ICC classes in terms of the abundance of somatic mutations and CNAs. First, this finding clearly shows the need for profiling both somatic mutations and CNAs to determine the molecular class and also to fully catalogue the potential oncogenic drivers in individual ICC genomes. Although WES has been mainly used to identify somatic mutations, it can be also used to call CNAs [27]. Thus, as demonstrated in our study, WES on tumor-normal matches may the choice for this dual purpose while whole-genomic sequencing can be also considered for optimal characterization of ICC genomes. Second, the cutoff of mutational abundance used to distinguish M class ICC genomes from C class was 90 coding mutations (corresponding to 1.3 mutations per Mb) and we observed that even the C class ICC genomes contain a substantial number of somatic mutations. Given that the three recurrent somatic mutations on *KRAS*, *TP53* and *APC* do not appear to be specifically enriched in M class, it can be assumed that these recurrent, potentially oncogenic drivers constitute early signatures of ICC genomes while ICC genomes will continue to develop with the preferential acquisition of either somatic mutations or CNAs, respectively. Third, we observed that one C class and two M class ICC genomes (three ICC both profiled by WES and RNA-seq) coincide with the expression-based proliferation and inflammation classes, respectively. This may suggest the potential overlap between the two molecular categorization schemes (i.e, gene expression- and genomic alteration-based ones). Fourth, in spite of the segregating patterns between the patient survival of M and C classes, the survival difference was not statistically significant in our cohort. This may be due to the small sample size of the study, and it requires a further validation in an extended set. In addition, four patients analyzed by WES (six patients in the entire cohort) received pre- or post-operative chemotherapy or radiation, and they were equally distributed into M- and C classes (i.e., two patients in each of the classes; Supplementary Table S6). Further evaluation will be required in a cohort of treatment-naive cases to examine the genomic impacts on the patient survival that are free from the additional therapeutic intervention. The potential relationship between somatic mutations (e.g., the mutually exclusive relationship between *KRAS* and *IDH1* mutations) also requires validation in an independent cohort [28]. Although a number of studies using gene panels have reported the prevalence of mutations for a limited number of cancer-related genes in CCA genomes (reviewed in [29]), the relationship between the abundance of mutations and copy number alterations can be only evaluated by exome- or genome-wide sequencing efforts.

WES and RNA-seq based analyses revealed candidate oncogenic drivers in ICC genomes. It should be noted that not all the mutations identified are ‘clinically actionable’, but recent efforts to identify alternative or combinatorial targets in relevant pathways or based on synthetic lethality are extending the list of druggable targets. For example, we found recurrent mutations of *KRAS* and *TP53* in ICC genomes that can be targeted by CDK4 and MDM2 inhibitors, respectively [30, 31]. Frequent mutations on epigenetic modifiers were also identified in ICC genomes, for which, synthetic lethality-based approaches have been recently proposed [32]. We also identified two *IDH1* mutations (two out of 17 ICC cases) that are known to be exclusive to ICC compared to other gastrointestinal tumors with potential diagnostic and therapeutic implications in ICC [13, 33]. Given that the majority of mutation profiling analyses of CCA genomes has used panel-based platforms targeting a small number of cancer-related genes [29], our study including a few additional WES-based studies [8, 9] may provide valuable information regarding the novel targets on ICC pathogenesis.

By comparing our results with previous reports, we observed one nonsense *PTPN3* mutation in a case but no additional mutation was found in nine phosphatase genes (*PTPN3, PTPRB, PTPRQ, PTPRS, PTPRZ1, SBF1, SBF2, MTMR3*, and *EYA1*) that are known to be frequently mutated in ICC [11], but the prevalence of mutations involving phosphatase as well as the frequent somatic mutations on *APC* and genes belonging to the TGFβ signaling pathway requires further validation in a larger cohort. While the APC/Wnt inactivation in ICC has been largely attributed to chromosomal losses [34], our results suggest that the *APC* truncating mutations may be more prevalent in ICC genomes than previously appreciated, often accompanying chromosomal losses to ensure the biallelic losses of *APC*.

We have used RNA-seq to call the somatic mutations and perform expression-based ICC categorization. The low overlap between the WES and RNA-seq based somatic mutations was noted, e.g., only 25% of WES mutations are expressed and detected by RNA-seq. Along with the impact of gene expression levels, the sensitivity of RNA-seq based mutation calling may also be diminished by posttranscriptional modifications as we have proposed with the example of *TGFBR1* nonsense mutation. Thus, the cataloguing somatic mutations by RNA-seq and their interpretation requires caution and it should be taken into accounts that low-frequent mutations or those in low-expressed genes are frequently missed.

In conclusion, we performed whole-exome and -transcriptome sequencing of 17 ICC cases (i) to obtain and characterize the landscape of somatic mutations, (ii) to classify ICC genomes into two classes with relative abundance of somatic mutations and CNAs and (iii) to compare the exome- and transcriptome-derived somatic variants. Along with a substantial level of mutational heterogeneity across ICC genomes observed, the presence of two ICC molecular classes suggests that whole-exome or -genome scale profiling of somatic mutations and CNAs will be required to fully understand the molecular pathogenesis of individual ICC case and also to identify the potentially druggable targets for personalized ICC therapeutics.

## MATERIALS AND METHODS

### Human ICC cases

Surgical specimens of 17 ICC patients were obtained from NCC (National Cancer Center, Republic of Korea). All patients were Koreans and the clinicopathologic features of the patients are summarized in Table 1. Among the patients, six received pre- or post-operative chemotherapy or radiation (Supplementary Table S6). This study received IRB approval, and include protocol number (NCCNSC13779) from NCC, Republic of Korea. Six out of 17 ICC patients received pre- or post-operative chemotherapy or radiations (Frozen tissue was cut and stained with hematoxylin. Microdissection was performed to obtain the tumor and adjacent non-tumor cells from hematoxylin-stained frozen sections. To extract the genomic DNA and RNA, we used DNeasy and RNeasy Blood and Tissue Kit (Qiagen, Hilden, Germany) according to the manufacturer's protocol.

### WES and somatic variants

WES was performed for the genomic DNA obtained from tumor and matched adjacent normal tissues. We used Agilent SureSelect Human All Exome 50 Mb kit (Agilent Technologies) and the genomic DNA library was prepared according to the manufacturer's instruction. We used Illumina HiSeq2000 platform to obtain 101 bp, paired-end sequencing reads. The alignment of sequencing reads onto the human genome reference (hg19) was done using Burrows-Wheeler alignment (BWA) algorithm [35]. Local realignment of sequencing reads and score recalibration were performed using the Genome Analysis ToolKit [36]. To call single nucleotide variants and small insertions/indels (indels), we used MuTect [37] and SomaticIndelDetector [36], respectively, by comparing the sequencing reads from tumor and matched normal genomes. For functional annotation of somatic variants in coding regions, we used ANNOVAR package [38].

### Inference of CNA from WES

We used VarScan2 to obtain the difference of sequencing read depth between the tumor and matched normal WES data [39]. After GC-correction, the read depth ratio was transformed into log_2_ scale. The log_2_ ratio of genomic bins were segmented using circular binary segmentation algorithm [40]. The segments with log_2_ ratio > 0.15 and < −0.15 are defined as chromosomal amplifications and deletions, respectively. We have used GISTIC (Genomic Identification of Significant Targets in Cancer) algorithm to define the recurrent focal alterations and the assessment of their significances [41].

### Transcriptome sequencing

We also used Illumina HiSeq2000 platform to obtain 101 bp, paired-end RNA-seq reads. The RNA-seq reads were mapped onto hg19 using STAR (Spliced Transcripts Alignment to a Reference) algorithm that is specialized in RNA-seq alignment and also showed better sensitivity than BWA [34] for RNA-seq [42]. STAR two-pass alignment steps comprise (i) the construction of human genome index (hg19) followed by the initial alignment of sequencing reads and (ii) the construction of the second genome index using the splice junction information from the first pass. The RNA-seq reads were then aligned onto the second genome index. We also used Genome Analysis ToolKit [36] to add the sequencing read groups, mark PCR duplicates and split the reads into exon segments followed by hard-clipping of overhanging sequences. The identification of the somatic mutations and indels as well as the annotation of coding variants were performed using MuTect, SomaticIndelDetector and ANNOVAR, respectively [36–38]. To obtain gene expression profiles, we aligned the sequencing reads using TopHat [43] and estimated the level of expression in terms of FPKM (fragments per kilobase per million) using CuffLinks [44]. FPKM was log-transformed and log_2_ (FPKM + 1) was used as the level of gene expression.

### Validation of gene mutation and gene expression

Gene mutations were confirmed by Sanger sequencing-based validation for the fourteen selected genes (*KRAS*, *TP53*, *APC*, *IDH1*, *PTPN3*, *RB1*, *MAP2K1*, *MED12*, *NF1*, *RAF1*, *XPO1, BAP1*, *ROBO1* and *SMAD7*) (Supplementary Figure S1) in frozen samples from ICC patients using the 20 somatic events primer sets. The presences of peaks consistent with minor mutant alleles were performed by direct DNA sequencing (ABI 3100 PRISM DNA Sequencer, Applied Biosystems, Foster City, CA, USA). Total RNAs were extracted from frozen samples of ten ICC patients using RNeasy Blood and Tissue Kit (Qiagen, Hilden, Germany), and reverse transcribed by using SuperScript II First-Strand Synthesis System (Life Technologies). Quantitative PCR was carried out according to the manufacturer's protocol of FastStart Essential DNA Green Master (Roche) by LightCycler^®^ 96 Real-Time PCR System (Roche) in a 20 μL reaction mixture composed of 10 μL 2x FastStart Essential DNA Green Mastermix, 4 μL H2O, 2 μL template DNA (approximately 20 ng/μL) and primers. Each reaction was performed in triplicate. The relative gene expression was calculated for each gene of interest by using the ΔΔCT method, where CT values were normalized to the housekeeping gene GAPDH.

### Statistical analyses

A Kaplan-Meier survival plot was generated using R (https://cran.r-project.org/). Log-rank test was performed to estimate the significant level of survival differences between M and C classes.

## SUPPLEMENTARY MATERIALS FIGURES AND TABLES


