# Supplementary material for: Two classes of intrahepatic cholangiocarcinoma defined by relative abundance of mutations and copy number alterations
Source: Oncotarget. 2016 Mar 18;7(17):23825–36. doi: 10.18632/oncotarget.8183 (PMC5029666; doi:10.18632/oncotarget.8183)
Supplement: Supplementary file 1 [file oncotarget-07-23825-s001.pdf]

Two classes of intrahepatic cholangiocarcinoma defined by relative abundance of mutations and copy number alterations

Supplementary Materials

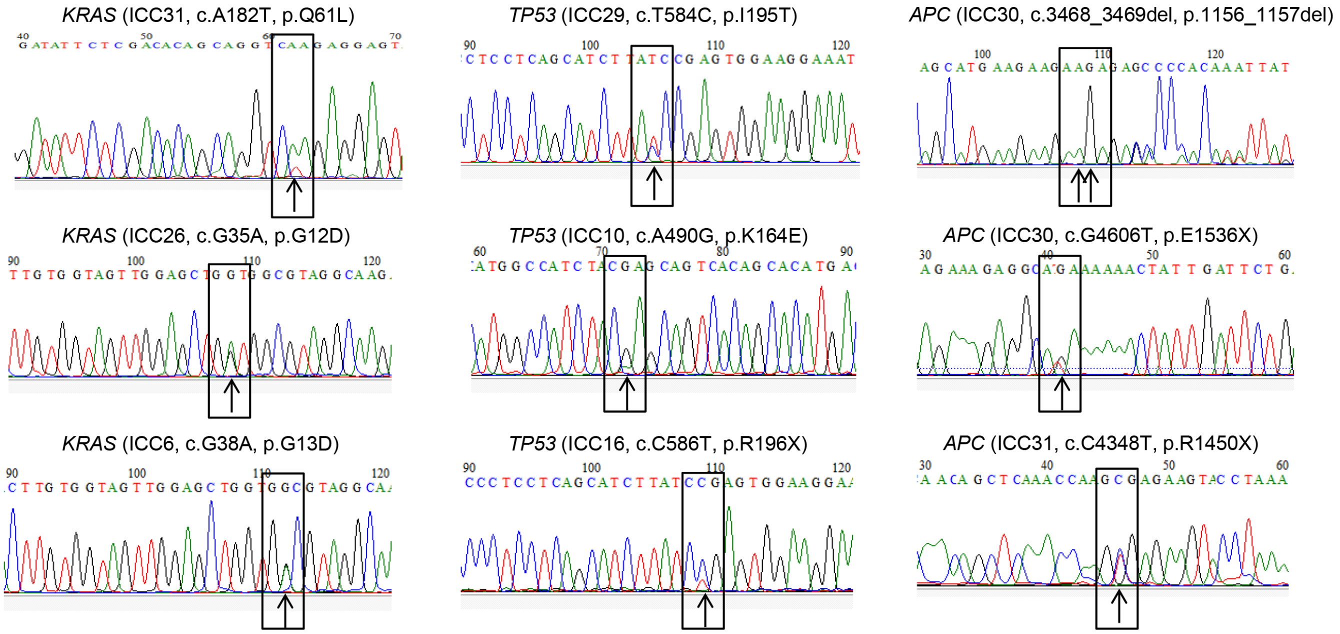

Supplementary Figure S1: Validation of somatic mutations. For selected single point mutations in each of five mutation categories, we performed Sanger sequencing for validation. Frequent ICC mutations (*KRAS, TP53*, *APC, IDH1*, and *PTPN3*) as well as mutation on histone modifying enzymes (*BAP1*), recurrent mutations in colorectal cancers (*ROBO1*), TGFβ signaling (*SMAD7*), and TARGET genes (*RB1, MAP2K1*, *MED12, NF1, RAF1, and XOP1*) were selected for validation. One microsatellite instability-like event (one-bp deletion of $(A)_{28}$) adjacent to splicing sites of *MSH2* was ignored. The chromatogram snapshots from the capillary sequencing are shown with arrows indicating the location of nucleotide substitution or deletion. Inbox indicates the nucleotide affected and those of 5′ and 3′ flaking. In case of splicing mutation (*RB1*), GT dinucleotide corresponding to splicing site is indicated.

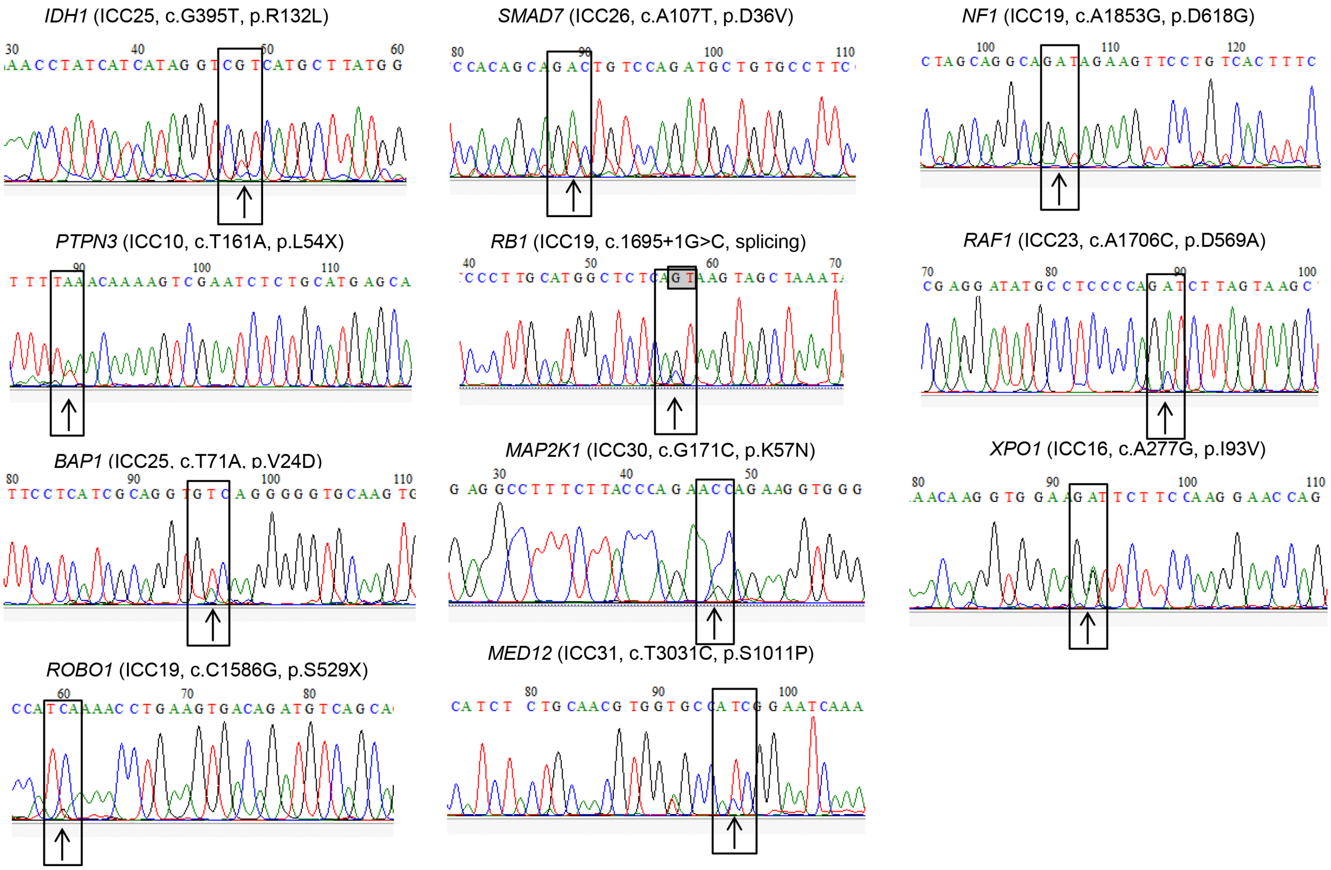

Supplementary Figure S1: Validation of somatic mutations. (Continued)

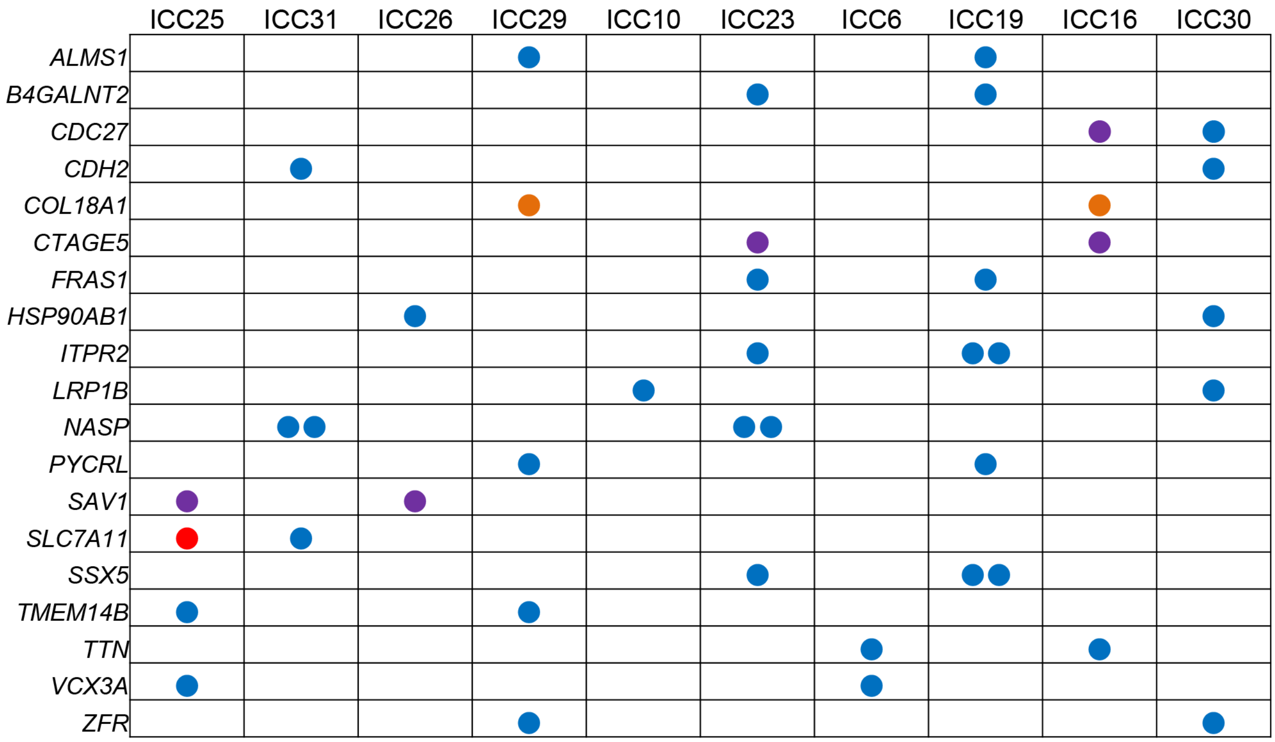

Supplementary Figure S2: Recurrent nonsilent mutations in ICC. As candidates of novel biomarkers, we report 19 nonsilent mutations observed in more than one ICC genomes but not listed in the main Figure 1. Blue, red, black, and purple circles represent missense, nonsense, frameshift, and splicing somatic variants, respectively.

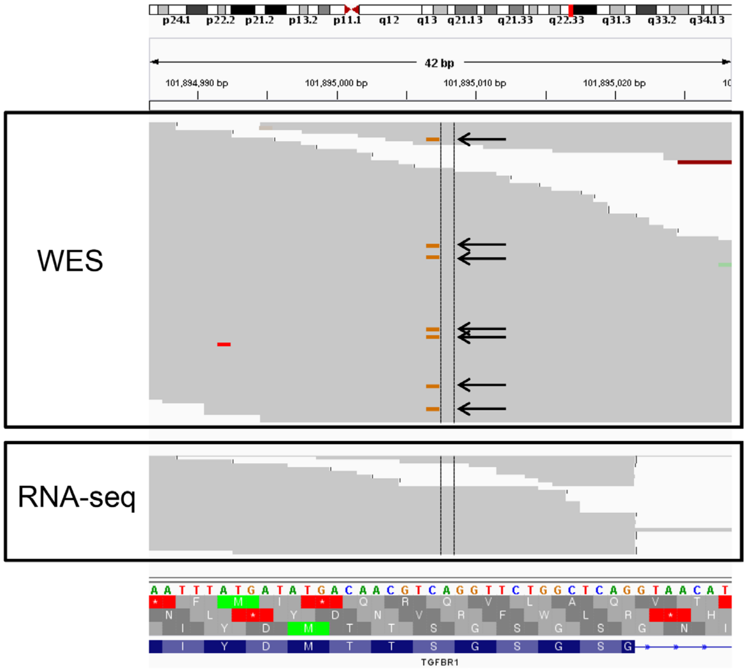

Supplementary Figure S3: A nonsense mutation at *TGFBR1* missing in RNA-seq. One nonsense mutation (Chr9:101895007/hg19, ICC6) at *TGFBR1* coding region is shown in WES and RNA-seq as IGVbrowser snapshots (above and below, respectively). The mutant alleles are shown with black arrows in WES. No corresponding mutant alleles are observed in the RNA-seq data.

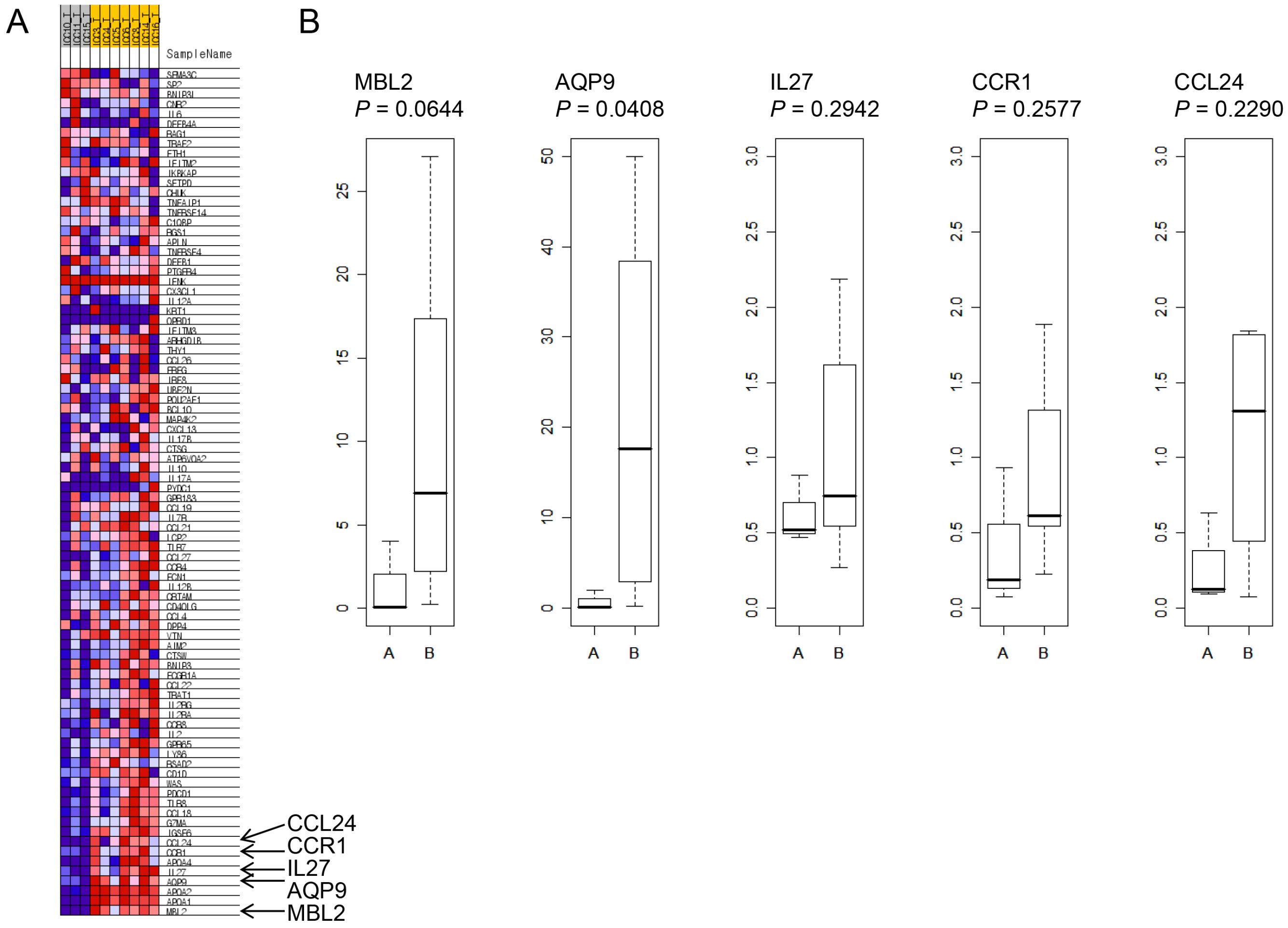

Supplementary Figure S4: Validation of gene expression. (**A**) The genes that belong to the 'Immune response' gene set are shown as a snapshot from GSEA output. This gene set showed top significant enrichment to the relative overexpression in class B compared to class A (yellow and grey at top). The genes are sorted in order of the relative expression. Five genes are selected for further validation. (**B**) The expression measured by quantitative PCR are shown for five selected genes. Significant difference between the expression of class A and B (*P* value; unpaired *t*-test) was observed for two genes (MBL2 and AQP9), but the relative overexpression of the genes in class B were consistently observed for the remaining genes of IL27, CCR1 and CCL24.

Supplementary Table S1: WES somatic variants

Case	Symbol	Category	Chr	Start	End	Ref	Obs	Description
ICC16	A2ML1	Silent	chr12	8975799	8975799	A	T	NM_144670:c.A84T:p.P28P
ICC10	AADAT	Missense	chr4	171009629	171009629	C	T	NM_016228:c.G154A:p.V52I
ICC26	AASDH	Missense	chr4	57248669	57248669	T	C	NM_181806:c.A325G:p.I109V
ICC30	ABCA1	Nonsense	chr9	107553269	107553269	G	C	NM_005502:c.C5861G:p.S1954X
ICC30	ABCC2	Missense	chr10	101591752	101591752	C	T	NM_000392:c.C3122T:p.A1041V
ICC26	ABTB2	Missense	chr11	34378995	34378995	C	T	NM_145804:c.G136A:p.A46T
ICC30	ACE	Missense	chr17	61570945	61570945	G	A	NM_001178057:c.G1339A:p.V447M
ICC19	ACTB	Missense	chr7	5567507	5567507	C	T	NM_001101:c.G1000A:p.E334K
ICC16	ACTL9	Missense	chr19	8808057	8808057	G	A	NM_178525:c.C995T:p.A332V
ICC30	ACTN2	Missense	chr1	236899007	236899007	C	T	NM_001103:c.C770T:p.A257V
ICC30	ACTN4	Missense	chr19	39207832	39207832	G	A	NM_004924:c.G1019A:p.R340H
ICC19	ACTR10	Missense	chr14	58675808	58675808	C	T	NM_018477:c.C325T:p.L109F
ICC26	ACTRT2	Silent	chr1	2938871	2938871	G	A	NM_080431:c.G621A:p.Q207Q
ICC30	ACVR1B	Missense	chr12	52379016	52379016	C	A	NM_004302:c.C1020A:p.N340K
ICC10	ADAM11	Missense	chr17	42854318	42854318	C	A	NM_002390:c.C1669A:p.L557I
ICC16	ADAMTS1	Missense	chr21	28210104	28210104	T	C	NM_006988:c.A2698G:p.S900G
ICC6	ADAMTS10	Missense	chr19	8670195	8670195	C	T	NM_030957:c.G137A:p.R46H
ICC30	ADCK5	Frameshift	chr8	145617534	145617549	TGGG GGTG CAAG GTGA	T	ADCK5:NM_174922:exon12:c.1256_ 1267T
ICC30	ADCY5	Missense	chr3	123166420	123166420	C	T	NM_183357:c.G973A:p.A325T
ICC30	ADCY5	Silent	chr3	123051444	123051444	G	A	NM_001199642:c.C435T:p.N145N
ICC30	ADCY8	Missense	chr8	131949376	131949376	C	T	NM_001115:c.G1424A:p.R475H
ICC30	ADIG	Silent	chr20	37214712	37214712	G	A	NM_001018082:c.G165A:p.E55E
ICC23	AGT	Missense	chr1	230841805	230841805	T	G	NM_000029:c.A998C:p.Q333P
ICC10	AHCTF1	Missense	chr1	247013960	247013960	G	C	NM_015446:c.C5375G:p.S1792C
ICC10	AHI1	Missense	chr6	135778702	135778702	T	G	NM_001134830:c.A1081C:p.I361L
ICC16	AHR	Silent	chr7	17379819	17379819	A	T	NM_001621:c.A2370T:p.P790P
ICC23	AIM1	Missense	chr6	106968693	106968693	C	T	NM_001624:c.C2386T:p.H796Y
ICC6	ALDH1L1	Missense	chr3	125872376	125872376	C	T	NM_001270365:c.G466A:p.E156K
ICC26	ALLC	Splicing	chr2	3721664	3721664	G	C	NM_018436:exon3:c.34-1G > C
ICC10	ALMS1	Nonframeshift	chr2	73613031	73613031	T	TGGA	ALMS1:NM_015120:exon1:c.35_35de linsTGGA
ICC19	ALMS1	Missense	chr2	73675168	73675168	C	T	NM_015120:c.C1511T:p.S504L
ICC19	ALMS1	Silent	chr2	73651768	73651768	G	A	NM_015120:c.G975A:p.S325S
ICC29	ALMS1	Missense	chr2	73747132	73747132	G	T	NM_015120:c.G9767T:p.R3256L
ICC30	ALOX15B	Silent	chr17	7948675	7948675	C	T	NM_001039130:c.C969T:p.C323C
ICC30	ANK2	Silent	chr4	114294251	114294251	C	T	NM_020977:c.C5361T:p.D1787D
ICC31	ANLN	Missense	chr7	36458908	36458908	A	T	NM_018685:c.A1689T:p.K563N
ICC30	ANO3	Missense	chr11	26463533	26463533	T	C	NM_031418:c.T115C:p.C39R

ICC30	ANP32C	Silent	chr4	165118807	165118807	C	A	NM_012403:c.G57T:p.V19V
ICC30	ANXA13	Silent	chr8	124714888	124714888	G	A	NM_004306:c.C180T:p.Y60Y
ICC25	AOC3	Missense	chr17	41003900	41003900	C	G	NM_003734:c.C540G:p.D180E
ICC30	APC	Frameshift	chr5	112174758	112174760	AAG	A	NM_000038:c.3468_3469del:p.1156_1157del
ICC30	APC	Nonsense	chr5	112175951	112175951	G	T	NM_000038:c.G4660T:p.E1554X
ICC31	APC	Nonsense	chr5	112175639	112175639	C	T	NM_000038:c.C4348T:p.R1450X
ICC19	APCDD1L	Missense	chr20	57036076	57036076	G	C	NM_153360:c.C1276G:p.L426V
ICC31	APEX2	Missense	chrX	55029522	55029522	G	T	NM_014481:c.G550T:p.V184L
ICC25	AQP1	Missense	chr7	30961398	30961398	C	G	NM_001185061:c.C176G:p.P59R
ICC30	AQPEP	Missense	chr5	115298621	115298621	G	A	NM_173800:c.G307A:p.V103M
ICC16	ARHGAP1	Missense	chr11	46717291	46717291	T	A	NM_004308:c.A148T:p.S50C
ICC16	ARHGAP32	Nonsense	chr11	128851449	128851449	G	A	NM_014715:c.C553T:p.Q185X
ICC16	ARHGAP39	Silent	chr8	145759525	145759525	G	A	NM_025251:c.C2676T:p.T892T
ICC10	ARHGAP5	Nonsense	chr14	32562474	32562474	C	T	NM_001030055:c.C2599T:p.R867X
ICC6	ARHGEF17	Missense	chr11	73073163	73073163	G	A	NM_014786:c.G4573A:p.V1525M
ICC23	ARHGEF40	Missense	chr14	21543099	21543099	G	A	NM_018071:c.G1210A:p.D404N
ICC29	ARHGEF6	Silent	chrX	135795470	135795470	A	T	NM_004840:c.T792A:p.L264L
ICC30	ARMC1	Silent	chr8	66525479	66525479	C	T	NM_018120:c.G465A:p.T155T
ICC29	ARNTL	Missense	chr11	13408194	13408194	G	C	NM_001030272:c.G1769C:p.S590T
ICC31	ASTN1	Missense	chr1	177001891	177001891	G	A	NM_004319:c.C566T:p.P189L
ICC31	ASTN2	Silent	chr9	119770372	119770372	G	A	NM_014010:c.C1437T:p.T479T
ICC16	ATAD2	Missense	chr8	124408453	124408453	G	T	NM_014109:c.C145A:p.P49T
ICC31	ATAD3B	Missense	chr1	1420466	1420466	T	A	NM_031921:c.T821A:p.F274Y
ICC19	ATIC	Missense	chr2	216177340	216177340	G	A	NM_004044:c.G139A:p.A47T
ICC6	ATP13A2	Missense	chr1	17312829	17312829	C	T	NM_001141974:c.G3128A:p.R1043H
ICC23	ATP4A	Missense	chr19	36047817	36047817	G	A	NM_000704:c.C1867T:p.R623W
ICC10	ATP6V0C	Silent	chr16	2569608	2569608	C	T	NM_001694:c.C330T:p.I110I
ICC23	ATP8A1	Missense	chr4	42524179	42524179	T	C	NM_001105529:c.A1900G:p.K634E
ICC16	ATXN3	Nonframeshift	chr14	92537354	92537354	C	CCTGCTGCTGCTG	NM_001164782:c.68_68delinsCAGCAGCAGCAGG:ATXN3
ICC30	AUTS2	Missense	chr7	69364311	69364311	C	T	NM_001127231:c.C349T:p.R117C
ICC16	B3GNT9	Missense	chr16	67183620	67183620	C	T	NM_033309:c.G769A:p.V257I
ICC19	B4GALNT2	Missense	chr17	47246909	47246909	C	A	NM_001159387:c.C1340A:p.T447N
ICC23	B4GALNT2	Missense	chr17	47210575	47210575	G	A	NM_153446:c.G188A:p.R63H
ICC23	BAALC	Missense	chr8	104153274	104153274	G	T	NM_001024372:c.G149T:p.G50V
ICC26	BACH1	Missense	chr21	30699535	30699535	A	G	NM_001186:c.A1390G:p.I464V
ICC23	BAGE, BAGE4, BAGE5	Missense	chr21	11098728	11098728	C	T	NM_001187:c.G10A:p.G4R
ICC23	BAHD1	Missense	chr15	40756680	40756680	G	A	NM_014952:c.G1986A:p.M662I
ICC10	BAI2	Missense	chr1	32205776	32205776	A	C	NM_001703:c.T1993G:p.F665V

ICC25	BAP1	Missense	chr3	52443621	52443621	A	T	NM_004656:c.T71A:p.V24D
ICC31	BASP1	Missense	chr5	17275998	17275998	G	A	NM_006317:c.C673A:p.L225I
ICC26	BCL6	Silent	chr3	187443371	187443371	C	T	NM_001134738:c.G1587A:p.R529R
ICC16	BCL9L	Missense	chr11	118779098	118779098	T	A	NM_182557:c.A293T:p.Q98L
ICC19	BCLAF1	Splicing	chr6	136589298	136589298	A	AT	NM_001077441:exon11:c.1878+2T > AT
ICC30	BIN1	Missense	chr2	127811534	127811534	C	T	NM_139344:c.G1057A:p.D353N
ICC10	BRAT1	Missense	chr7	2581777	2581777	G	A	NM_152743:c.C992T:p.T331M
ICC16	BVES	Silent	chr6	105581294	105581294	T	A	NM_001199563:c.A159T:p.A53A
ICC30	C11orf63	Missense	chr11	122775018	122775018	C	T	NM_024806:c.C730T:p.R244C
ICC31	C11orf80	Missense	chr11	66563810	66563810	G	A	NM_024650:c.G692A:p.R231H
ICC19	C11orf88	Silent	chr11	111385710	111385710	G	A	NM_001100388:c.G201A:p.A67A
ICC29	C11orf9	Missense	chr11	61536776	61536776	C	G	NM_001127392:c.C443G:p.S148C
ICC19	C12orf4	Missense	chr12	4643436	4643436	C	T	NM_020374:c.G211A:p.E71K
ICC30	C15orf27	Missense	chr15	76448975	76448975	G	C	NM_152335:c.G258C:p.K86N
ICC30	C1orf129	Silent	chr1	170985399	170985399	G	A	NM_001163629:c.G1830A:p.R610R
ICC16	C1orf177	Missense	chr1	55277824	55277824	C	T	NM_001110533:c.C724T:p.R242W
ICC30	C1QC	Missense	chr1	22974253	22974253	G	A	NM_001114101:c.G715A:p.G239S
ICC6	C1QTNF5	Frameshift	chr11	119210502	119210502	G	GC	C1QTNF5:NM_015645:exon15:c.271_271delinsGC
ICC16	C22orf29	Nonsense	chr22	19838797	19838797	C	A	NM_024627:c.G988T:p.E330X
ICC25	C2orf42	Silent	chr2	70408653	70408653	G	T	NM_017880:c.C465A:p.V155V
ICC30	C5orf42	Missense	chr5	37153858	37153858	T	A	NM_023073:c.A8195T:p.D2732V
ICC30	C6orf222	Missense	chr6	36291094	36291094	G	A	NM_001010903:c.C1447T:p.R483W
ICC30	C6orf222	Nonsense	chr6	36290159	36290159	G	C	NM_001010903:c.C1532G:p.S511X
ICC29	C8A	Missense	chr1	57378205	57378205	A	G	NM_000562:c.A1510G:p.N504D
ICC31	C8orf34	Missense	chr8	69552655	69552655	C	A	NM_052958:c.C1150A:p.P384T
ICC10	CACNA1E	Silent	chr1	181480632	181480632	C	T	NM_000721:c.C498T:p.I166I
ICC16	CACNA1E	Missense	chr1	181548343	181548343	G	A	NM_000721:c.G752A:p.C251Y
ICC16	CACNA1G	Missense	chr17	48696161	48696161	A	G	NM_198376:c.A5471G:p.N1824S
ICC23	CADM1	Missense	chr11	115102120	115102120	T	G	NM_001098517:c.A515C:p.K172T
ICC30	CAPN3	Silent	chr15	42700486	42700486	C	A	NM_173088:c.C342A:p.G114G
ICC16	CAPN9	Missense	chr1	230937323	230937323	A	G	NM_016452:c.A1976G:p.H659R
ICC6	CARM1	Missense	chr19	11018786	11018786	C	T	NM_199141:c.C418T:p.R140W
ICC10	CASP8AP2	Missense	chr6	90577760	90577760	A	G	NM_001137667:c.A4751G:p.Y1584C
ICC26	CASR	Missense	chr3	121980808	121980808	A	G	NM_000388:c.A926G:p.Q309R
ICC23	CBLN3	Missense	chr14	24898026	24898026	T	A	NM_001039771:c.A235T:p.S79C
ICC23	CC2D2A	Silent	chr4	15558956	15558956	T	C	NM_001080522:c.T2655C:p.P885P
ICC6	CCBP2	Missense	chr3	42906449	42906449	A	G	NM_001296:c.A455G:p.Y152C
ICC19	CCDC126	Silent	chr7	23651132	23651132	C	T	NM_138771:c.C198T:p.N66N
ICC6	CCDC129	Silent	chr7	31617967	31617967	G	A	NM_194300:c.G1089A:p.K363K
ICC29	CCDC173	Missense	chr2	170502450	170502450	C	G	NM_001085447:c.G1560C:p.Q520H

ICC30	CCDC178	Silent	chr18	30926284	30926284	G	A	NM_001105528:c.C549T:p.D183D
ICC31	CCDC24	Missense	chr1	44461753	44461753	G	A	NM_152499:c.G845A:p.G282E
ICC16	CCDC40	Missense	chr17	78061553	78061553	A	G	NM_001243342:c.A2597G:p.N866S
ICC6	CCDC42	Missense	chr17	8638829	8638829	C	T	NM_144681:c.G593A:p.R198H
ICC31	CD163L1	Missense	chr12	7522148	7522148	C	A	NM_174941:c.G3844T:p.V1282L
ICC30	CD1E	Silent	chr1	158324319	158324319	T	C	NM_001042583:c.T211C:p.L71L
ICC16	CDC27	Splicing	chr17	45221350	45221351	TA	T	
ICC30	CDC27	Missense	chr17	45234303	45234303	G	C	NM_001114091:c.C818G:p.A273G
ICC23	CDH11	Missense	chr16	65038621	65038621	G	A	NM_001797:c.C152T:p.S51F
ICC30	CDH16	Silent	chr16	66946316	66946316	G	A	NM_001204746:c.C1086T:p.L362L
ICC19	CDH19	Silent	chr18	64172151	64172151	T	C	NM_021153:c.A2217G:p.A739A
ICC30	CDH2	Missense	chr18	25572711	25572711	C	T	NM_001792:c.G1252A:p.A418T
ICC31	CDH2	Missense	chr18	25568587	25568587	G	A	NM_001792:c.C1642T:p.P548S
ICC10	CDH23	Silent	chr10	73545471	73545471	G	A	NM_022124:c.G5796A:p.P1932P
ICC10	CDKN2A	Nonframeshift	chr9	21974726	21974744	GCCT CCAG CAGC GCCC GCA	G	NM_000077:c.83_101C:CDKN2A
ICC10	CDT1	Missense	chr16	88871859	88871859	C	T	NM_030928:c.C500T:p.A167V
ICC16	CECR6	Missense	chr22	17601086	17601086	A	T	NM_031890:c.T932A:p.F311Y
ICC16	CENPC1	Missense	chr4	68357909	68357909	A	C	NM_001812:c.T2504G:p.I835S
ICC6	CENPO	Silent	chr2	25040603	25040603	G	A	NM_001199803:c.G813A:p.T271T
ICC6	CES2	Missense	chr16	66969362	66969362	C	G	NM_003869:c.C16G:p.R6G
ICC6	CFHR1	Silent	chr1	196797357	196797357	A	G	NM_002113:c.A588G:p.T196T
ICC16	CFTR	Missense	chr7	117267689	117267689	G	T	NM_000492:c.G3582T:p.E1194D
ICC29	CHD1	Missense	chr5	98207804	98207804	C	A	NM_001270:c.G3812T:p.G1271V
ICC30	CHST9	Missense	chr18	24628462	24628462	C	T	NM_001256316:c.G127A:p.V43M
ICC19	CIDEB	Silent	chr14	24775667	24775667	T	C	NM_014430:c.A249G:p.A83A
ICC26	CLEC18B	Missense	chr16	74451970	74451970	G	C	NM_001011880:c.C443G:p.T148S
ICC26	CLEC18B	Silent	chr16	74451960	74451960	C	T	NM_001011880:c.G453A:p.T151T
ICC16	CLUL1	Missense	chr18	627219	627219	T	A	NM_014410:c.T546A:p.D182E
ICC25	CMYA5	Missense	chr5	79025738	79025738	G	A	NM_153610:c.G1150A:p.D384N
ICC29	CNNM1	Missense	chr10	101122098	101122098	A	T	NM_020348:c.A1973T:p.E658V
ICC29	CNOT6L	Missense	chr4	78647460	78647460	C	T	NM_144571:c.G1316A:p.R439K
ICC16	CNTNAP4	Missense	chr16	76523606	76523606	C	T	NM_138994:c.C1687T:p.H563Y
ICC16	COL18A1	Frameshift	chr21	46924425	46924434	CGGC CCC CCA	C	NM_030582:c.3363_3364C:COL18A1
ICC29	COL18A1	Frameshift	chr21	46924425	46924434	CGGC CCC CCA	C	NM_030582:c.3363_3364C:COL18A1
ICC23	COL27A1	Missense	chr9	117047032	117047032	G	T	NM_032888:c.G3962T:p.G1321V
ICC31	COL5A2	Missense	chr2	189915371	189915371	G	A	NM_000393:c.C2963T:p.T988M

ICC6	COL5A3	Missense	chr19	10116299	10116299	G	A	NM_015719:c.C529T:p.R177C
ICC16	COL6A3	Silent	chr2	238249327	238249327	C	A	NM_057166:c.G6411T:p.T2137T
ICC30	COL6A6	Silent	chr3	130285832	130285832	G	A	NM_001102608:c.G1569A:p.L523L
ICC10	COL8A1	Silent	chr3	99514455	99514455	C	A	NM_020351:c.C1710A:p.P570P
ICC6	CPNE2	Missense	chr16	57155595	57155595	G	A	NM_152727:c.G790A:p.E264K
ICC6	CPQ	Missense	chr8	97797465	97797465	G	A	NM_016134:c.G340A:p.E114K
ICC10	CPXM2	Missense	chr10	125651031	125651031	G	A	NM_198148:c.C145T:p.R49C
ICC29	CR1	Missense	chr1	207793323	207793323	G	A	NM_000573:c.G5815A:p.E1939K
ICC6	CSDE1	Missense	chr1	115261316	115261316	C	T	NM_001242893:c.G2174A:p.R725H
ICC30	CSMD2	Silent	chr1	34238192	34238192	C	T	NM_052896:c.G1704A:p.S568S
ICC30	CST4	Silent	chr20	23667800	23667800	C	A	NM_001899:c.G267T:p.V89V
ICC16	CTAGE5	Splicing	chr14	39784004	39784006	GTA	G	
ICC23	CTAGE5	Splicing	chr14	39784004	39784006	GTA	G	
ICC26	CTDNEP1	Silent	chr17	7147543	7147543	G	A	NM_001143775:c.C696T:p.S232S
ICC16	CTTNBP2	Missense	chr7	117432351	117432351	G	A	NM_033427:c.C899T:p.T300I
ICC26	CTU2	Silent	chr16	88776642	88776642	G	A	NM_001012759:c.G234A:p.A78A
ICC31	CXorf59	Missense	chrX	36091370	36091370	C	A	NM_173695:c.C305A:p.T102K
ICC30	CYP11B1	Missense	chr8	143956558	143956558	C	T	NM_000497:c.G1213A:p.V405M
ICC25	CYP2A13	Missense	chr19	41595958	41595958	C	T	NM_000766:c.C350T:p.A117V
ICC25	CYP2A13	Silent	chr19	41595959	41595959	G	A	NM_000766:c.G351A:p.A117A
ICC16	CYP3A5	Missense	chr7	99246019	99246019	G	A	NM_000777:c.C1418T:p.P473L
ICC31	CYP4F2	Missense	chr19	15989696	15989696	G	C	NM_001082:c.C1448G:p.A483G
ICC31	CYYR1	Silent	chr21	27840903	27840903	A	G	NM_052954:c.T382C:p.L128L
ICC30	DAB2IP	Missense	chr9	124329507	124329507	G	A	NM_032552:c.G40A:p.E14K
ICC30	DCAF8L2	Nonframeshift	chrX	27765399	27765411	AGAGGAGGAGGAG	A	DCAF8L2:NM_001136533:exon1:c.387_399A
ICC30	DCDC5	Silent	chr11	30915831	30915831	C	T	NM_020869:c.G2178A:p.Q726Q
ICC19	DCHS1	Missense	chr11	6644707	6644707	C	T	NM_003737:c.G8200A:p.D2734N
ICC26	DCP1B	Nonsense	chr12	2061836	2061836	T	A	NM_152640:c.A1270T:p.R424X
ICC10	DDI2	Silent	chr1	15944177	15944177	C	G	NM_032341:c.C12G:p.T4T
ICC19	DDX53	Missense	chrX	23019577	23019577	A	G	NM_182699:c.A1403G:p.D468G
ICC19	DDX53	Silent	chrX	23019587	23019587	C	T	NM_182699:c.C1413T:p.I471I
ICC25	DFNB31	Missense	chr9	117266799	117266799	G	C	NM_001173425:c.C283G:p.R95G
ICC16	DGAT2	Missense	chr11	75501245	75501245	G	A	NM_001253891:c.G134A:p.S45N
ICC30	DGKB	Splicing	chr7	14775821	14775822	TG	T	
ICC23	DGKG	Missense	chr3	185969556	185969556	T	C	NM_001080744:c.A1678G:p.I560V
ICC23	DHX15	Missense	chr4	24529625	24529625	A	C	NM_001358:c.T2310G:p.N770K
ICC31	DHX30	Splicing	chr3	47889669	47889669	G	A	NM_138615:exon15:c.2287-1G > A
ICC30	DIDO1	Silent	chr20	61511899	61511899	C	T	NM_001193369:c.G5409A:p.Q1803Q
ICC16	DISC1	Silent	chr1	231856613	231856613	T	A	NM_001164552:c.T1137A:p.A379A

ICC25	DLC1	Splicing	chr8	13251061	13251061	C	T	NM_182643:exon5:c.1314+1G > A
ICC10	DLG5	Missense	chr10	79569483	79569483	G	A	NM_004747:c.C4469T:p.A1490V
ICC23	DLGAP2	Silent	chr8	1497687	1497687	G	T	NM_004745:c.G828T:p.G276G
ICC31	DMKN	Splicing	chr19	35991322	35991322	C	A	NM_001190347:exon14:c.1165-1G > T
ICC26	DMWD	Missense	chr19	46289493	46289493	G	C	NM_004943:c.C1261G:p.L421V
ICC30	DNAH10	Missense	chr12	124341718	124341718	G	A	NM_207437:c.G6200A:p.R2067H
ICC29	DNAH14	Nonsense	chr1	225490979	225490979	G	A	NM_001373:c.G8474A:p.W2825X
ICC30	DNAH5	Silent	chr5	13923470	13923470	G	A	NM_001369:c.C357T:p.N119N
ICC30	DNAH6	Silent	chr2	84861735	84861735	C	A	NM_001370:c.C4623A:p.L1541L
ICC16	DNAH8	Missense	chr6	38840745	38840745	C	A	NM_001206927:c.C7301A:p.S2434Y
ICC29	DNAH9	Missense	chr17	11696833	11696833	C	T	NM_001372:c.C8075T:p.S2692F
ICC16	DNAJC9	Missense	chr10	75006474	75006474	T	A	NM_015190:c.A293T:p.E98V
ICC16	DNHD1	Silent	chr11	6566183	6566183	G	A	NM_144666:c.G4014A:p.L1338L
ICC10	DNM1L	Missense	chr12	32832335	32832335	A	T	NM_005690:c.A38T:p.D13V
ICC26	DOCK10	Silent	chr2	225657746	225657746	C	T	NM_014689:c.G5256A:p.S1752S
ICC29	DOCK2	Nonsense	chr5	169138990	169138990	C	T	NM_004946:c.C1534T:p.R512X
ICC25	DOT1L	Splicing	chr19	2185929	2185929	G	T	NM_032482:exon3:c.200+1G > T
ICC25	DOT1L	Splicing	chr19	2211842	2211842	G	A	NM_032482:exon16:c.1557+1G > A)
ICC30	DSCAM	Missense	chr21	41559851	41559851	C	T	NM_001389:c.G2617A:p.E873K
ICC6	DSCAML1	Missense	chr11	117351241	117351241	C	T	NM_020693:c.G2882A:p.R961Q
ICC10	DSPP	Nonframeshift	chr4	88537072	88537081	TAGC AGT GAC	T	DSPP:NM_014208:exon5:c.3258_32 67T
ICC25	DSPP	Nonframeshift	chr4	88537072	88537081	TAGC AGT GAC	T	DSPP:NM_014208:exon5:c.3258_32 67T
ICC25	DSPP	Nonframeshift	chr4	88537297	88537306	CAGC AGC GAT	C	DSPP:NM_014208:exon5:c.3483_34 92C
ICC30	DSPP	Nonframeshift	chr4	88537072	88537081	TAGC AGT GAC	T	DSPP:NM_014208:exon5:c.3258_32 67T
ICC6	DSPP	Nonframeshift	chr4	88536928	88536937	CAGC AGC GAT	C	DSPP:NM_014208:exon5:c.3114_31 23C
ICC19	DTX3L	Missense	chr3	122287536	122287536	A	T	NM_138287:c.A600T:p.Q200H
ICC16	DUOXA1, DUOXA2	Missense	chr15	45409922	45409922	T	C	NM_207581:c.T778C:p.C260R
ICC26	DVL2	Missense	chr17	7133404	7133404	G	A	NM_004422:c.C481T:p.R161W
ICC16	DYNC1I1	Missense	chr7	95499272	95499272	A	C	NM_001135557:c.A392C:p.K131T
ICC10	ECHDC2	Nonsense	chr1	53370437	53370437	G	A	NM_001198962:c.C490T:p.R164X
ICC29	EEF1D	Missense	chr8	144671252	144671252	C	A	NM_001130053:c.G1000T:p.A334S
ICC6	EFHC2	Silent	chrX	44120438	44120438	C	T	NM_025184:c.G489A:p.R163R
ICC19	EIF4G1	Missense	chr3	184035281	184035281	G	T	NM_198244:c.G59T:p.G20V
ICC30	ELF3	Missense	chr1	201983140	201983140	G	A	NM_001114309:c.G989A:p.S330N

ICC10	ELFN2	Silent	chr22	37770495	37770495	G	A	NM_052906:c.C1080T:p.C360C
ICC10	EML1	Missense	chr14	100363532	100363532	C	T	NM_004434:c.C728T:p.T243M
ICC16	EMR2	Missense	chr19	14884803	14884803	G	A	NM_001271052:c.C146T:p.P49L
ICC30	EPAS1	Nonframeshift	chr2	46605201	46605204	CCAG	C	EPAS1:NM_001430:exon10:c.1418_1421C
ICC6	EPB41L1	Missense	chr20	34775678	34775678	A	G	NM_001258331:c.A680G:p.H227R
ICC16	EPB42	Missense	chr15	43507424	43507424	T	A	NM_000119:c.A389T:p.Q130L
ICC31	EPHB2	Missense	chr1	23232596	23232596	G	A	NM_004442:c.G1885A:p.G629R
ICC29	EPS8L1	Splicing	chr19	55594982	55594982	A	T	NM_133180:exon14:c.1357-2A > T
ICC19	EPSTI1	Missense	chr13	43491753	43491753	C	G	NM_033255:c.G665C:p.S222T
ICC23	ETV7	Silent	chr6	36343811	36343811	G	T	NM_001207035:c.C144A:p.R48R
ICC6	F10	Missense	chr13	113783786	113783786	G	T	NM_000504:c.G91T:p.A31S
ICC23	F13A1	Missense	chr6	6305594	6305594	T	G	NM_000129:c.A309C:p.E103D
ICC19	F5	Missense	chr1	169510565	169510565	G	C	NM_000130:c.C3763G:p.Q1255E
ICC30	FAM123B	Nonsense	chrX	63412530	63412530	G	A	NM_152424:c.C637T:p.Q213X
ICC6	FAM154A	Silent	chr9	18928785	18928785	A	G	NM_153707:c.T690C:p.C230C
ICC16	FAM180A	Missense	chr7	135418804	135418804	G	C	NM_205855:c.C441G:p.I147M
ICC19	FAM192A	Silent	chr16	57197953	57197953	T	C	NM_024946:c.A507G:p.K169K
ICC30	FAM196B	Silent	chr5	169310465	169310465	A	G	NM_001129891:c.T438C:p.S146S
ICC29	FAM71E2	Silent	chr19	55870127	55870127	G	T	NM_001145402:c.C2109A:p.I703I
ICC10	FAM83H	Silent	chr8	144810854	144810854	C	T	NM_198488:c.G777A:p.A259A
ICC26	FAR2	Silent	chr12	29469778	29469778	C	G	NM_018099:c.T960G:p.P320P
ICC26	FARP2	Missense	chr2	242429458	242429458	A	G	NM_014808:c.A2503G:p.S835G
ICC16	FAT3	Missense	chr11	92086917	92086917	G	A	NM_001008781:c.G1639A:p.V547I
ICC16	FBL	Missense	chr19	40327275	40327275	T	A	NM_001436:c.A716T:p.Q239L
ICC25	FBRS	Missense	chr16	30680559	30680559	G	A	NM_001105079:c.G976A:p.A326T
ICC6	FBXO28	Missense	chr1	224340869	224340869	T	C	NM_015176:c.T542C:p.L181S
ICC26	FBXW2	Missense	chr9	123550290	123550290	C	T	NM_012164:c.G247A:p.V83I
ICC10	FCGBP	Missense	chr19	40389657	40389657	T	G	NM_003890:c.A8525C:p.N2842T
ICC26	FCGR2A	Silent	chr1	161487839	161487839	C	T	NM_001136219:c.C855T:p.D285D
ICC30	FCGR3B	Missense	chr1	161600944	161600944	C	T	NM_001244753:c.G49A:p.G17S
ICC10	FCHO1	Missense	chr19	17886301	17886301	C	T	NM_001161359:c.C832T:p.R278W
ICC31	FFAR1	Missense	chr19	35842818	35842818	T	C	NM_005303:c.T364C:p.Y122H
ICC30	FGD3	Missense	chr9	95738639	95738639	C	T	NM_001083536:c.C101T:p.A34V
ICC30	FGFR1OP2	Silent	chr12	27110592	27110592	C	T	NM_001171887:c.C312T:p.Y104Y
ICC26	FHDC1	Missense	chr4	153881781	153881781	A	G	NM_033393:c.A728G:p.Y243C
ICC31	FLG	Missense	chr1	152283719	152283719	T	G	NM_002016:c.A3643C:p.S1215R
ICC25	FLG2	Missense	chr1	152324245	152324245	C	G	NM_001014342:c.G6017C:p.R2006T
ICC30	FLT4	Silent	chr5	180048630	180048630	C	T	NM_002020:c.G1932A:p.A644A
ICC29	FMN2	Missense	chr1	240341266	240341266	T	A	NM_020066:c.T1828A:p.S610T
ICC19	FMNL1	Missense	chr17	43317858	43317858	G	C	NM_005892:c.G1096C:p.E366Q

ICC16	FN3KRP	Missense	chr17	80674699	80674699	G	A	NM_024619:c.G68A:p.G23E
ICC10	FNDC1	Missense	chr6	159653544	159653544	G	A	NM_032532:c.G2000A:p.R667Q
ICC10	FNDC3B	Silent	chr3	172048427	172048427	G	T	NM_001135095:c.G1476T:p.T492T
ICC19	FNDC3B	Missense	chr3	172065020	172065020	G	A	NM_001135095:c.G2383A:p.A795T
ICC16	FOXB1	Silent	chr15	60297528	60297528	A	T	NM_012182:c.A366T:p.P122P
ICC10	FOXG1	Missense	chr14	29237168	29237168	C	G	NM_005249:c.C683G:p.S228C
ICC16	FOXI1	Missense	chr5	169533485	169533485	T	C	NM_012188:c.T524C:p.L175P
ICC30	FOXL1	Silent	chr16	86612417	86612417	C	T	NM_005250:c.C88T:p.L30L
ICC19	FRAS1	Missense	chr4	79366718	79366718	C	T	NM_001166133:c.C5708T:p.S1903L
ICC23	FRAS1	Missense	chr4	79238564	79238564	C	G	NM_001166133:c.C1862G:p.S621C
ICC6	FSD2	Nonsense	chr15	83455713	83455713	G	A	NM_001007122:c.C430T:p.Q144X
ICC26	FSHR	Missense	chr2	49190706	49190706	A	C	NM_181446:c.T1176G:p.I392M
ICC6	FTMT	Silent	chr5	121187808	121187808	C	T	NM_177478:c.C150T:p.A50A
ICC23	FTO	Silent	chr16	53859925	53859925	C	T	NM_001080432:c.C273T:p.L91L
ICC30	FUBP3	Missense	chr9	133493210	133493210	G	A	NM_003934:c.G594A:p.M198I
ICC19	FZD2	Missense	chr17	42635759	42635759	T	C	NM_001466:c.T703C:p.F235L
ICC23	FZD6	Missense	chr8	104342200	104342200	C	T	NM_001164615:c.C1859T:p.S620L
ICC6	GAA	Missense	chr17	78086800	78086800	C	T	NM_000152:c.C2014T:p.R672W
ICC6	GAB4	Missense	chr22	17449202	17449202	C	A	NM_001037814:c.G1009T:p.V337F
ICC31	GABRA6	Missense	chr5	161116672	161116672	C	T	NM_000811:c.C560T:p.T187M
ICC29	GALNTL2	Missense	chr3	16237350	16237350	T	A	NM_054110:c.T623A:p.L208Q
ICC19	GATA5	Missense	chr20	61048541	61048541	C	G	NM_080473:c.G617C:p.G206A
ICC6	GATSL3	Missense	chr22	30682352	30682352	C	T	NM_001037666:c.G643A:p.A215T
ICC31	GFRAL	Missense	chr6	55223813	55223813	G	T	NM_207410:c.G829T:p.A277S
ICC19	GJA8	Missense	chr1	147380369	147380369	C	T	NM_005267:c.C287T:p.A96V
ICC6	GLG1	Missense	chr16	74640712	74640712	G	A	NM_001145666:c.C281T:p.A94V
ICC16	GLI2	Silent	chr2	121555043	121555043	A	G	NM_005270:c.A147G:p.G49G
ICC30	GLIPR1	Silent	chr12	75874786	75874786	C	T	NM_006851:c.C126T:p.N42N
ICC6	GNAS	Missense	chr20	57484420	57484420	C	T	NM_001077489:c.C556T:p.R186C
ICC19	GNB4	Silent	chr3	179137234	179137234	C	T	NM_021629:c.G156A:p.R52R
ICC25	GPR101	Missense	chrX	136113398	136113398	G	T	NM_054021:c.C436A:p.R146S
ICC25	GPR112	Silent	chrX	135430074	135430074	G	A	NM_153834:c.G4209A:p.V1403V
ICC30	GPR77	Silent	chr19	47844185	47844185	C	T	NM_018485:c.G129T:p.R43R
ICC23	GPS2	Missense	chr17	7217860	7217860	T	C	NM_004489:c.A151G:p.R51G
ICC6	GRB7	Silent	chr17	37899446	37899446	G	A	NM_001030002:c.G477A:p.E159E
ICC6	GRIA2	Missense	chr4	158282709	158282709	G	A	NM_000826:c.G2311A:p.V771I
ICC10	GRIA4	Missense	chr11	105769082	105769082	G	A	NM_001112812:c.G814A:p.V272I
ICC29	GRIN2C	Missense	chr17	72838783	72838783	A	T	NM_000835:c.T3493A:p.C1165S
ICC16	GRIN3B	Missense	chr19	1008178	1008178	C	G	NM_138690:c.C2354G:p.S785C
ICC10	GRM3	Silent	chr7	86468567	86468567	C	A	NM_000840:c.C1737A:p.G579G
ICC16	GRM3	Silent	chr7	86468840	86468840	C	T	NM_000840:c.C2010T:p.F670F

ICC19	GRM8	Missense	chr7	126086303	126086303	G	A	NM_000845:c.C2554T:p.R852C
ICC31	GSDMD	Missense	chr8	144642819	144642819	C	T	NM_024736:c.C457T:p.R153C
ICC25	GSR	Silent	chr8	30560644	30560644	A	G	NM_000637:c.T606C:p.G202G
ICC23	GTDC2	Missense	chr3	43121833	43121833	A	T	NM_032806:c.T1091A:p.L364H
ICC6	GTPBP1	Missense	chr22	39126647	39126647	G	A	NM_004286:c.G1948A:p.G650R
ICC29	GUF1	Missense	chr4	44693726	44693726	A	G	NM_021927:c.A1523G:p.N508S
ICC19	GXYLT2	Missense	chr3	72937598	72937598	C	A	NM_001080393:c.C214A:p.P72T
ICC10	H6PD	Frameshift	chr1	9324325	9324332	GGGC TCGA	G	H6PD:NM_004285:exon5:c.1773_17 80G
ICC29	HACE1	Missense	chr6	105297059	105297059	T	C	NM_020771:c.A284G:p.D95G
ICC30	HAND1	Missense	chr5	153855452	153855452	G	T	NM_004821:c.C562A:p.P188T
ICC19	HAUS5	Missense	chr19	36110636	36110636	C	T	NM_015302:c.C1382T:p.T461M
ICC30	HAVCR1	Silent	chr5	156479481	156479481	C	T	NM_012206:c.G564A:p.T188T
ICC19	HDAC4	Missense	chr2	240085556	240085556	G	A	NM_006037:c.C554T:p.A185V
ICC19	HEATR5A	Missense	chr14	31785095	31785095	G	A	NM_015473:c.C4099T:p.H1367Y
ICC6	HERC1	Missense	chr15	63946457	63946457	C	T	NM_003922:c.G10151A:p.R3384Q
ICC10	HERC2	Missense	chr15	28473466	28473466	C	A	NM_004667:c.G5362T:p.V1788L
ICC6	HIC2	Silent	chr22	21800438	21800438	C	T	NM_015094:c.C1254T:p.S418S
ICC30	HIF3A	Missense	chr19	46825222	46825222	C	T	NM_022462:c.C1127T:p.S376L
ICC16	HK1	Missense	chr10	71119700	71119700	A	T	NM_000188:c.A274T:p.I92F
ICC6	HLX	Nonframeshift	chr1	221053600	221053600	A	AGCA GCAA CAGC CGCC	HLX:NM_021958:exon1:c.401_401deli nsAGCAGCAACAGCCGCC
ICC10	HMBS	Splicing	chr11	118959418	118959418	G	T	NM_001258208:exon3:c.160+1G > T
ICC31	HMCN1	Missense	chr1	186105803	186105803	C	G	NM_031935:c.C13316G:p.P4439R
ICC10	HOXD13	Silent	chr2	176957942	176957942	A	G	NM_000523:c.A324G:p.P108P
ICC26	HSP90AB1	Missense	chr6	44221104	44221104	A	G	NM_007355:c.A2054G:p.K685R
ICC30	HSP90AB1	Missense	chr6	44221047	44221047	C	T	NM_007355:c.C1997T:p.S666F
ICC30	HSP90AB1	Silent	chr6	44221048	44221048	T	G	NM_007355:c.T1998G:p.S666S
ICC6	HSPBP1	Missense	chr19	55785939	55785939	G	A	NM_012267:c.C467T:p.A156V
ICC16	HSPD1	Silent	chr2	198363501	198363501	C	T	NM_002156:c.G72A:p.R24R
ICC23	HSPD1	Silent	chr2	198363501	198363501	C	T	NM_002156:c.G72A:p.R24R
ICC25	HSPD1	Silent	chr2	198363501	198363501	C	T	NM_002156:c.G72A:p.R24R
ICC25	HSPG2	Nonsense	chr1	22216504	22216504	G	A	NM_005529:c.C544T:p.Q182X
ICC29	HTT	Nonframeshift	chr4	3076672	3076672	A	ACCG CCGC CG	HTT:NM_002111:exon1:c.120_120deli nsACCGCCGCCG
ICC23	HVCN1	Missense	chr12	111099093	111099093	G	T	NM_001256413:c.C122A:p.P41Q
ICC19	ICAM4	Missense	chr19	10397824	10397824	G	A	NM_001039132:c.G136A:p.V46M
ICC25	IDH1	Missense	chr2	209113112	209113112	C	A	NM_005896:c.G395T:p.R132L
ICC31	IFT81	Missense	chr12	110565283	110565283	A	G	NM_001143779:c.A98G:p.Q33R
ICC30	IGF2R	Silent	chr6	160526095	160526095	C	T	NM_000876:c.C7455T:p.D2485D

ICC25	IGLL1	Missense	chr22	23915574	23915574	G	A	NM_020070:c.C521T:p.A174V
ICC25	IGSF9B	Silent	chr11	133801975	133801975	C	T	NM_014987:c.G1101A:p.Q367Q
ICC23	IL16	Missense	chr15	81592627	81592627	T	G	NM_004513:c.T857G:p.L286R
ICC19	IL27RA	Missense	chr19	14153596	14153596	T	C	NM_004843:c.T763C:p.W255R
ICC23	INADL	Missense	chr1	62340969	62340969	A	C	NM_176877:c.A2890C:p.N964H
ICC23	IPO4	Missense	chr14	24657826	24657826	T	C	NM_024658:c.A88G:p.I30V
ICC16	IQSEC3	Missense	chr12	234978	234978	C	A	NM_001170738:c.C803A:p.A268D
ICC16	IRGC	Silent	chr19	44223727	44223727	G	A	NM_019612:c.G1017A:p.S339S
ICC23	IRS2	Missense	chr13	110436707	110436707	T	G	NM_003749:c.A1694C:p.Q565P
ICC31	IRX4	Missense	chr5	1882022	1882022	G	A	NM_016358:c.C197T:p.S66L
ICC16	ISL1	Missense	chr5	50683472	50683472	C	T	NM_002202:c.C367T:p.R123W
ICC16	ISLR	Missense	chr15	74468158	74468158	T	A	NM_005545:c.T959A:p.L320Q
ICC29	ITGAL	Silent	chr16	30529150	30529150	T	A	NM_001114380:c.T2814A:p.P938P
ICC29	ITGB3	Splicing	chr17	45377842	45377842	A	G	NM_000212:exon12:c.1914-2A > G
ICC29	ITGB4	Missense	chr17	73752521	73752521	C	T	NM_001005619:c.C4669T:p.R1557W
ICC6	ITIH5	Missense	chr10	7618557	7618557	G	A	NM_032817:c.C1195T:p.R399C
ICC19	ITPR2	Missense	chr12	26755578	26755578	T	C	NM_002223:c.A3524G:p.K1175R
ICC19	ITPR2	Missense	chr12	26755582	26755582	T	A	NM_002223:c.A3520T:p.N1174Y
ICC23	ITPR2	Missense	chr12	26648165	26648165	T	C	NM_002223:c.A5102G:p.N1701S
ICC19	ITPR3	Missense	chr6	33646519	33646519	C	T	NM_002224:c.C3875T:p.T1292M
ICC29	JARID2	Missense	chr6	15512515	15512515	G	A	NM_001267040:c.G2513A:p.C838Y
ICC29	KANK3	Frameshift	chr19	8400105	8400106	GT	G	KANK3:NM_198471:exon3:c.605_606C
ICC25	KAT6B	Missense	chr10	76784742	76784742	G	C	NM_001256468:c.G2850C:p.R950S
ICC10	KAT8	Missense	chr16	31131665	31131665	C	T	NM_032188:c.C292T:p.R98W
ICC25	KBTBD3	Nonsense	chr11	105923933	105923933	G	A	NM_152433:c.C1483T:p.Q495X
ICC30	KCNC1	Silent	chr11	17794171	17794171	G	A	NM_004976:c.G1530A:p.S510S
ICC16	KCNG1	Silent	chr20	49620807	49620807	G	A	NM_002237:c.C1311T:p.G437G
ICC6	KCNG2	Missense	chr18	77659278	77659278	G	A	NM_012283:c.G863A:p.R288Q
ICC30	KCNJ10	Missense	chr1	160011823	160011823	G	A	NM_002241:c.C500T:p.A167V
ICC6	KCNJ8	Missense	chr12	21926336	21926336	G	A	NM_004982:c.C215T:p.T72M
ICC23	KCNQ5	Missense	chr6	73904256	73904256	G	A	NM_001160134:c.G1588A:p.A530T
ICC30	KCNS2	Missense	chr8	99440299	99440299	G	A	NM_020697:c.G92A:p.R31H
ICC19	KCTD16	Silent	chr5	143586424	143586424	C	T	NM_020768:c.C147T:p.S49S
ICC16	KCTD20	Missense	chr6	36437888	36437888	G	A	NM_173562:c.G14A:p.R5H
ICC6	KDM4D	Missense	chr11	94730832	94730832	G	A	NM_018039:c.G296A:p.G99E
ICC16	KDM5B	Silent	chr1	202733229	202733229	T	C	NM_006618:c.A756G:p.R252R
ICC29	KHDC1	Missense	chr6	74001741	74001741	C	A	NM_001251874:c.G186T:p.K62N
ICC10	KHK	Missense	chr2	27315287	27315287	G	A	NM_000221:c.G180A:p.M60I
ICC31	KIAA0226	Missense	chr3	197423859	197423859	T	A	NM_014687:c.A1323T:p.E441D
ICC23	KIAA0368	Missense	chr9	114246693	114246693	G	T	NM_001080398:c.C220A:p.R74S

ICC23	KIAA1009	Missense	chr6	84859363	84859363	A	C	NM_014895:c.T3689G:p.I1230R
ICC29	KIAA1324	Missense	chr1	109716318	109716318	T	A	NM_020775:c.T811A:p.Y271N
ICC30	KIAA1429	Missense	chr8	95503865	95503865	C	G	NM_015496:c.G5081C:p.R1694T
ICC19	KIAA1751	Silent	chr1	1916854	1916854	C	G	NM_001080484:c.G471C:p.S157S
ICC16	KIF14	Splicing	chr1	200544820	200544820	C	T	NM_014875:exon23:c.3466-1G > A
ICC30	KIF17	Silent	chr1	21031184	21031184	C	T	NM_001122819:c.G879A:p.T293T
ICC31	KIF22	Missense	chr16	29808298	29808298	C	A	NM_007317:c.C155A:p.P52Q
ICC10	KIF26A	Missense	chr14	104642717	104642717	C	T	NM_015656:c.C3592T:p.R1198W
ICC26	KLHL24	Missense	chr3	183396929	183396929	G	T	NM_017644:c.G1658T:p.R553L
ICC26	KLHL24	Silent	chr3	183396930	183396930	G	T	NM_017644:c.G1659T:p.R553R
ICC31	KLRD1	Missense	chr12	10466072	10466072	G	T	NM_007334:c.G286T:p.A96S
ICC26	KRAS	Missense	chr12	25398284	25398284	C	T	NM_004985:c.G35A:p.G12D
ICC31	KRAS	Missense	chr12	25380276	25380276	T	A	NM_004985:c.A182T:p.Q61L
ICC6	KRAS	Missense	chr12	25398281	25398281	C	T	NM_004985:c.G38A:p.G13D
ICC29	KRT10	Nonframeshift	chr17	38975251	38975266	GCCG CCGC CGGA GCTT	G	KRT10:NM_000421:exon7:c.1521_1 536C
ICC6	KRT37	Missense	chr17	39580609	39580609	C	T	NM_003770:c.G167A:p.R56H
ICC16	KRT78	Nonsense	chr12	53241824	53241824	G	A	NM_173352:c.C466T:p.Q156X
ICC16	KRTAP10-6	Silent	chr21	46011298	46011298	G	A	NM_198688:c.C1068T:p.S356S
ICC19	KRTAP13-3	Missense	chr21	31798092	31798092	G	C	NM_181622:c.C139G:p.Q47E
ICC30	KRTAP19-6	Nonsense	chr21	31914024	31914024	G	T	NM_181612:c.C129A:p.C43X
ICC16	KRTAP21-1	Missense	chr21	32127540	32127540	C	A	NM_181619:c.G157T:p.G53C
ICC25	KRTAP4-5	Nonframeshift	chr17	39305775	39305775	T	TGGC AGCA GCTG GGGC	KRTAP4-5:NM_033188:exon1:c.245_2 45delinsGCCCCAGCTGCTGCCA
ICC30	KRTAP4-9	Missense	chr17	39261809	39261809	T	C	NM_001146041:c.T169C:p.S57P
ICC16	LAMA5	Missense	chr20	60888413	60888413	A	C	NM_005560:c.T8762G:p.L2921R
ICC30	LCE2D	Silent	chr1	152636788	152636788	T	C	NM_178430:c.T207C:p.G69G
ICC26	LCN6	Missense	chr9	139642884	139642884	C	T	NM_198946:c.G52A:p.A18T
ICC10	LDHC	Missense	chr11	18451374	18451374	G	C	NM_002301:c.G335C:p.R112P
ICC31	LDLRAD3	Missense	chr11	36248871	36248871	G	A	NM_174902:c.G691A:p.V231I
ICC10	LEPR	Nonsense	chr1	66067607	66067607	C	G	NM_001198687:c.C1367G:p.S456X
ICC31	LILRA1	Missense	chr19	55106778	55106778	G	A	NM_006863:c.G572A:p.R191H
ICC25	LILRB4	Missense	chr19	55175822	55175822	C	A	NM_001081438:c.C541A:p.P181T
ICC23	LIN9	Silent	chr1	226485450	226485450	T	C	NM_001270409:c.A177G:p.K59K
ICC19	LIPH	Missense	chr3	185252743	185252743	T	C	NM_139248:c.A227G:p.H76R
ICC16	LMO7	Missense	chr13	76427354	76427354	G	T	NM_015842:c.G3792T:p.Q1264H
ICC19	LOC100129520	Silent	chrX	124455510	124455510	G	A	NM_001195272:c.G1542A:p.E514E
ICC6	LOC100653515	Missense	chr17	76887844	76887844	C	T	NM_001243540:c.G742A:p.A248T
ICC25	LOC402160	Missense	chr4	2461863	2461863	C	T	NM_001193282:c.C1555T:p.R519W

ICC6	LONRF1	Missense	chr8	12586568	12586568	C	A	NM_152271:c.G1852T:p.A618S
ICC6	LRCH1	Missense	chr13	47315861	47315861	G	A	NM_015116:c.G2065A:p.E689K
ICC29	LRFN5	Missense	chr14	42356927	42356927	A	T	NM_152447:c.A1099T:p.T367S
ICC6	LRP1	Silent	chr12	57572304	57572304	C	T	NM_002332:c.C4524T:p.T1508T
ICC10	LRP1B	Missense	chr2	141253205	141253205	G	T	NM_018557:c.C8963A:p.T2988N
ICC30	LRP1B	Missense	chr2	141004693	141004693	G	T	NM_018557:c.C13286A:p.P4429Q
ICC23	LRP2	Silent	chr2	169989168	169989168	A	G	NM_004525:c.T13644C:p.D4548D
ICC16	LRP8	Silent	chr1	53726226	53726226	G	T	NM_033300:c.C1456A:p.R486R
ICC26	LRRC29	Missense	chr16	67241585	67241585	A	T	NM_001004055:c.T595A:p.F199I
ICC6	LRRC72	Missense	chr7	16566641	16566641	G	A	NM_001195280:c.G64A:p.E22K
ICC16	LRRCC1	Missense	chr8	86038951	86038951	A	G	NM_033402:c.A1300G:p.R434G
ICC30	LRRK2	Missense	chr12	40689336	40689336	G	T	NM_198578:c.G2986T:p.D996Y
ICC10	LRRN1	Nonsense	chr3	3888101	3888101	T	G	NM_020873:c.T1776G:p.Y592X
ICC31	LRSAM1	Silent	chr9	130242168	130242168	C	T	NM_001005374:c.C954T:p.N318N
ICC10	LRTOMT	Silent	chr11	71806035	71806035	C	T	NM_001205138:c.C276T:p.H92H
ICC19	LUZP1	Missense	chr1	23419569	23419569	G	C	NM_001142546:c.C1186G:p.Q396E
ICC31	LYPD6B	Missense	chr2	150069628	150069628	G	A	NM_177964:c.G451A:p.E151K
ICC10	MAML2	Nonframeshift	chr11	95825374	95825383	TTGC TGC TGC	T	MAML2:NM_032427:exon2:c.1812_ 1821A
ICC19	MAML2	Nonframeshift	chr11	95825371	95825374	CTGT	C	MAML2:NM_032427:exon2:c.1821_ 1824G
ICC19	MAML2	Silent	chr11	95825362	95825362	C	T	NM_032427:c.G1833A:p.Q611Q
ICC31	MAML2	Nonframeshift	chr11	95825374	95825383	TTGC TGC TGC	T	MAML2:NM_032427:exon2:c.1812_ 1821A
ICC30	MAP2K1	Missense	chr15	66727455	66727455	G	C	NM_002755:c.G171C:p.K57N
ICC30	MAP3K13	Splicing	chr3	185155234	185155234	G	C	NM_001242314:exon4:c.476-1G > C,NM_004721:exon3:c.476-1G > C
ICC23	MAPRE2	Silent	chr18	32585473	32585473	G	A	NM_001143827:c.G42A:p.R14R
ICC23	MB21D2	Missense	chr3	192517263	192517263	G	A	NM_178496:c.C388T:p.R130C
ICC30	MBTD1	Missense	chr17	49296318	49296318	A	C	NM_017643:c.T376G:p.L126V
ICC23	MCEE	Missense	chr2	71337246	71337246	T	C	NM_032601:c.A385G:p.N129D
ICC26	MCM3AP	Missense	chr21	47697571	47697571	G	C	NM_003906:c.C1728G:p.D576E
ICC30	MDH1	Silent	chr2	63816497	63816497	G	A	NM_001199111:c.G36A:p.T12T
ICC10	MDM1	Silent	chr12	68724930	68724930	C	A	NM_001205028:c.G90T:p.V30V
ICC31	MED12	Missense	chrX	70347792	70347792	T	C	NM_005120:c.T3031C:p.S1011P
ICC29	MED15	Nonframeshift	chr22	20920813	20920813	A	ACAG	NM_001003891:c.750_750delinsACA G:MED15
ICC23	MEGF11	Silent	chr15	66386798	66386798	G	A	NM_032445:c.C336T:p.C112C
ICC19	MEGF8	Silent	chr19	42879793	42879793	G	A	NM_001410:c.G7203A:p.R2401R
ICC31	MEGF8	Missense	chr19	42847688	42847688	G	A	NM_001410:c.G1573A:p.V525I
ICC10	MESP2	Silent	chr15	90320149	90320149	G	A	NM_001039958:c.G561A:p.G187G

ICC30	MESP2	Nonframeshift	chr15	90320134	90320146	AGGG CAGG GGC AG	A	MESP2:NM_001039958:exon1:c.546_558A
ICC30	MET	Silent	chr7	116339633	116339633	G	A	NM_000245:c.G495A:p.Q165Q
ICC30	MFAP2	Splicing	chr1	17302995	17302995	C	A	NM_001135248:exon7:c.283+1G > T,NM_017459:exon7:c.286+1G > T,NM_002403:exon7:c.286+1G > T,NM_001135247:exon7:c.283+1G > T
ICC19	MGP	Missense	chr12	15038715	15038715	A	G	NM_000900:c.T11C:p.L4P
ICC16	MINOS1	Missense	chr1	19950062	19950062	T	A	NM_001204082:c.T159A:p.H53Q
ICC31	MKI67	Missense	chr10	129905068	129905068	G	A	NM_001145966:c.C3956T:p.S1319F
ICC31	MKI67	Missense	chr10	129906301	129906301	C	T	NM_001145966:c.G2723A:p.R908Q
ICC31	MLL3	Missense	chr7	151945042	151945042	C	T	NM_170606:c.G2477A:p.G826D
ICC30	MOGAT2	Missense	chr11	75428961	75428961	C	T	NM_025098:c.C28T:p.P10S
ICC23	MRM1	Missense	chr17	34958297	34958297	T	G	NM_024864:c.T58G:p.F20V
ICC19	MRPL15	Missense	chr8	55047872	55047872	C	T	NM_014175:c.C29T:p.A10V
ICC19	MRPL39	Missense	chr21	26976134	26976134	C	G	NM_017446:c.G394C:p.D132H
ICC26	MRPS11	Silent	chr15	89020222	89020222	A	G	NM_176805:c.A315G:p.R105R
ICC19	MRPS9	Missense	chr2	105713648	105713648	G	T	NM_182640:c.G965T:p.R322L
ICC25	MSH2	Splicing	chr2	47641559	47641560	TA	T	
ICC26	MT1B	Silent	chr16	56686499	56686499	C	T	NM_005947:c.C45T:p.C15C
ICC23	MTA3	Missense	chr2	42871348	42871348	A	G	NM_020744:c.A463G:p.R155G
ICC10	MUC12	Silent	chr7	100643367	100643367	C	T	NM_001164462:c.C9523T:p.L3175L
ICC30	MUC12	Silent	chr7	100643367	100643367	C	T	NM_001164462:c.C9523T:p.L3175L
ICC29	MUC17	Missense	chr7	100679334	100679334	C	A	NM_001040105:c.C4637A:p.T1546N
ICC6	MUC4	Nonframeshift	chr3	195512373	195512373	G	GGAT	MUC4:NM_018406:exon2:c.6078_6078delinsATCC
ICC6	MUC4	Missense	chr3	195508307	195508307	T	G	NM_018406:c.A10144C:p.I3382L
ICC6	MUC4	Missense	chr3	195511076	195511076	T	A	NM_018406:c.A7375T:p.T2459S
ICC23	MUT	Silent	chr6	49421310	49421310	T	C	NM_000255:c.A1071G:p.S357S
ICC10	MVD	Missense	chr16	88722569	88722569	C	T	NM_002461:c.G547A:p.A183T
ICC31	MXRA5	Silent	chrX	3261731	3261731	G	A	NM_015419:c.C144T:p.S48S
ICC26	MYBPC2	Silent	chr19	50957351	50957351	G	A	NM_004533:c.G1824A:p.A608A
ICC26	MYH1	Missense	chr17	10399587	10399587	T	C	NM_005963:c.A4936G:p.N1646D
ICC16	MYH15	Missense	chr3	108224665	108224665	A	G	NM_014981:c.T160C:p.C54R
ICC6	MYH6	Missense	chr14	23869968	23869968	G	A	NM_002471:c.C1360T:p.R454C
ICC30	MYLK2	Missense	chr20	30419645	30419645	G	A	NM_033118:c.G1564A:p.V522I
ICC29	MYO16	Nonsense	chr13	109318260	109318260	C	T	NM_001198950:c.C55T:p.R19X
ICC25	MYO5B	Silent	chr18	47364009	47364009	G	A	NM_001080467:c.C5016T:p.G1672G
ICC30	MYT1	Silent	chr20	62839236	62839236	C	T	NM_004535:c.C687T:p.T229T
ICC19	MYT1L	Missense	chr2	1895889	1895889	C	T	NM_015025:c.G2197A:p.A733T
ICC23	NASP	Missense	chr1	46073299	46073299	C	T	NM_001195193:c.C524T:p.A175V

ICC23	NASP	Missense	chr1	46073323	46073323	G	A	NM_001195193:c.G548A:p.G183E
ICC31	NASP	Missense	chr1	46073299	46073299	C	T	NM_001195193:c.C524T:p.A175V
ICC31	NASP	Missense	chr1	46073323	46073323	G	A	NM_001195193:c.G548A:p.G183E
ICC25	NBPF10	Silent	chr1	145311110	145311110	G	C	NM_001039703:c.G1809C:p.R603R
ICC10	NBR1	Missense	chr17	41352493	41352493	C	T	NM_005899:c.C2336T:p.A779V
ICC30	NCAM2	Missense	chr21	22652904	22652904	T	A	NM_004540:c.T62A:p.L21H
ICC30	NCAM2	Silent	chr21	22881254	22881254	C	T	NM_004540:c.C2160T:p.D720D
ICC16	NCKAP5	Silent	chr2	133542860	133542860	G	A	NM_207363:c.C1524T:p.S508S
ICC29	NCOA6	Missense	chr20	33345157	33345157	T	A	NM_001242539:c.A1394T:p.Q465L
ICC6	NCOA7	Nonsense	chr6	126203587	126203587	C	T	NM_001199621:c.C277T:p.R93X
ICC26	NCOR1	Silent	chr17	15976849	15976849	T	G	NM_001190440:c.A3753C:p.P1251P
ICC30	NCR1	Silent	chr19	55423573	55423573	G	A	NM_001242356:c.G435A:p.T145T
ICC19	NF1	Missense	chr17	29552120	29552120	A	G	NM_000267:c.A1853G:p.D618G
ICC19	NKD2	Frameshift	chr5	1035506	1035507	CG	C	NM_001271082:c.577_578C:NKD2
ICC25	NKX6-2	Frameshift	chr10	134599226	134599226	A	AG	NKX6-2:NM_177400:exon1:c.227_227delinsCT
ICC6	NOSTRIN	Missense	chr2	169707904	169707904	C	T	NM_001171632:c.C761T:p.T254M
ICC10	NOVA1	Silent	chr14	27064755	27064755	G	A	NM_002515:c.C141T:p.D47D
ICC26	NPC1	Missense	chr18	21140411	21140411	T	C	NM_000271:c.A665G:p.N222S
ICC23	NR2E3	Missense	chr15	72104394	72104394	C	T	NM_014249:c.C449T:p.P150L
ICC31	NRXN1	Silent	chr2	51255079	51255079	G	A	NM_001135659:c.C333T:p.D111D
ICC30	NSFL1C	Missense	chr20	1435695	1435695	C	T	NM_016143:c.G361A:p.D121N
ICC30	NT5DC2	Silent	chr3	52558720	52558720	C	T	NM_001134231:c.G1440A:p.A480A
ICC30	NT5DC3	Silent	chr12	104208739	104208739	C	A	NM_001031701:c.G369T:p.R123R
ICC6	NTN1	Silent	chr17	8926560	8926560	C	T	NM_004822:c.C870T:p.H290H
ICC26	NTSR2	Silent	chr2	11809710	11809710	C	T	NM_012344:c.G546A:p.T182T
ICC23	NUDCD1	Missense	chr8	110302144	110302144	A	G	NM_001128211:c.T572C:p.I191T
ICC6	OCA2	Missense	chr15	28267708	28267708	G	C	NM_000275:c.C585G:p.S195R
ICC19	OPA1	Missense	chr3	193364929	193364929	G	T	NM_130831:c.G1557T:p.M519I
ICC6	OPRM1	Silent	chr6	154360922	154360922	C	T	NM_000914:c.C243T:p.C81C
ICC16	OR10G9	Missense	chr11	123893801	123893801	A	G	NM_001001953:c.A82G:p.I28V
ICC19	OR10H2	Silent	chr19	15838910	15838910	C	T	NM_013939:c.C57T:p.A19A
ICC6	OR10H2	Missense	chr19	15838972	15838972	T	A	NM_013939:c.T119A:p.L40Q
ICC23	OR10T2	Missense	chr1	158368318	158368318	C	A	NM_001004475:c.G939T:p.M313I
ICC6	OR10X1	Missense	chr1	158549346	158549346	A	G	NM_001004477:c.T344C:p.L115S
ICC6	OR13D1	Missense	chr9	107457081	107457081	A	G	NM_001004484:c.A379G:p.I127V
ICC10	OR2L8	Missense	chr1	248112937	248112937	C	T	NM_001001963:c.C778T:p.R260C
ICC19	OR2T4	Frameshift	chr1	248524971	248524971	C	CTCTA	OR2T4:NM_001004696:exon1:c.89_89delinsCTCTA
ICC29	OR4A15	Missense	chr11	55135707	55135707	G	C	NM_001005275:c.G348C:p.E116D
ICC16	OR52E8	Missense	chr11	5878551	5878551	G	A	NM_001005168:c.C382T:p.R128C
ICC29	OR6A2	Missense	chr11	6816831	6816831	C	T	NM_003696:c.G109A:p.V37M

ICC10	OSCP1	Missense	chr1	36904406	36904406	C	T	NM_145047:c.G218A:p.R73H
ICC19	OTOGL	Missense	chr12	80690724	80690724	G	T	NM_173591:c.G3019T:p.D1007Y
ICC26	OVGP1	Nonframeshift	chr1	111957501	111957525	CTCA CAGA CTGA TGAC TCAC AGG GG	C	OVGP1:NM_002557:exon11:c.1598_ 1622G
ICC19	OXSM	Splicing	chr3	25833435	25833435	A	C	NM_017897:c.A924C:p.S308S
ICC31	P4HB	Missense	chr17	79817158	79817158	G	A	NM_000918:c.C251T:p.A84V
ICC30	PABPC4L	Nonsense	chr4	135121319	135121319	G	A	NM_001114734:c.C1030T:p.R344X
ICC25	PAK7	Missense	chr20	9546950	9546950	G	A	NM_177990:c.C1072T:p.P358S
ICC19	PAPL	Missense	chr19	39589206	39589206	C	A	NM_001004318:c.C230A:p.T77N
ICC23	PARD3B	Missense	chr2	206480341	206480341	A	T	NM_057177:c.A3215T:p.Q1072L
ICC29	PARD6G	Missense	chr18	77917716	77917716	G	A	NM_032510:c.C1069T:p.R357C
ICC6	PARK2	Missense	chr6	162683694	162683694	G	A	NM_004562:c.C275T:p.A92V
ICC23	PARP15	Missense	chr3	122296630	122296630	C	T	NM_001113523:c.C116T:p.A39V
ICC30	PARP4	Missense	chr13	25016762	25016762	G	A	NM_006437:c.C3509T:p.T1170I
ICC30	PATL2	Missense	chr15	44965497	44965497	G	A	NM_001145112:c.C382T:p.R128W
ICC30	PATZ1	Silent	chr22	31740914	31740914	A	C	NM_014323:c.T675G:p.S225S
ICC29	PAX2	Missense	chr10	102546708	102546708	T	A	NM_003987:c.T625A:p.Y209N
ICC30	PCDH17	Missense	chr13	58207860	58207860	G	A	NM_001040429:c.G1180A:p.G394R
ICC16	PCDHA1	Silent	chr5	140167498	140167498	C	A	NM_018900:c.C1623A:p.G541G
ICC30	PCDHB15	Silent	chr5	140626775	140626775	C	T	NM_018935:c.C1629T:p.S543S
ICC30	PCDHGA4	Missense	chr5	140734951	140734951	C	T	NM_018917:c.C184T:p.R62C
ICC29	PCF11	Silent	chr11	82879852	82879852	A	C	NM_015885:c.A2475C:p.P825P
ICC6	PCLO	Missense	chr7	82584709	82584709	A	G	NM_014510:c.T5560C:p.S1854P
ICC19	PCSK7	Missense	chr11	117077005	117077005	C	T	NM_004716:c.G2066A:p.S689N
ICC19	PCSK7	Missense	chr11	117077009	117077009	A	C	NM_004716:c.T2062G:p.L688V
ICC26	PDE1B	Missense	chr12	54955378	54955378	C	G	NM_001165975:c.C10G:p.P4A
ICC25	PDE6B	Missense	chr4	629679	629679	A	G	NM_000283:c.A632G:p.K211R
ICC16	PDE8A	Silent	chr15	85656666	85656666	G	T	NM_173454:c.G1035T:p.A345A
ICC30	PENK	Silent	chr8	57358458	57358458	G	A	NM_006211:c.C55T:p.L19L
ICC19	PEX6	Missense	chr6	42946485	42946485	C	A	NM_000287:c.G404T:p.G135V
ICC31	PGLS	Silent	chr19	17627038	17627038	C	T	NM_012088:c.C345T:p.P115P
ICC23	PHF2	Silent	chr9	96439007	96439007	G	T	NM_005392:c.G2964T:p.P988P
ICC10	PHF3	Silent	chr6	64408114	64408114	T	C	NM_015153:c.T2682C:p.G894G
ICC16	PHLDB3	Silent	chr19	43999650	43999650	T	C	NM_198850:c.A948G:p.S316S
ICC19	PIGC	Silent	chr1	172411670	172411670	C	A	NM_002642:c.G93T:p.R31R
ICC16	PIK3R4	Missense	chr3	130435311	130435311	C	G	NM_014602:c.G2260C:p.G754R
ICC30	PITPNM3	Missense	chr17	6406870	6406870	G	T	NM_001165966:c.C143A:p.S48Y
ICC16	PKDREJ	Missense	chr22	46656474	46656474	G	T	NM_006071:c.C2746A:p.P916T

ICC29	PKLR	Silent	chr1	155270034	155270034	T	C	NM_000298:c.A138G:p.Q46Q
ICC30	PLA2G4E	Missense	chr15	42342846	42342846	G	A	NM_001206670:c.C56T:p.P19L
ICC6	PLAU	Missense	chr10	75674570	75674570	C	T	NM_001145031:c.C815T:p.A272V
ICC31	PLB1	Silent	chr2	28805355	28805355	C	T	NM_001170585:c.C1683T:p.C561C
ICC23	PLCB4	Silent	chr20	9353739	9353739	C	T	NM_000933:c.C732T:p.S244S
ICC16	PLCL1	Silent	chr2	198949594	198949594	T	C	NM_006226:c.T1353C:p.D451D
ICC30	PLIN4	Missense	chr19	4510530	4510530	G	A	NM_001080400:c.C3400T:p.R1134C
ICC23	PLXNA4	Missense	chr7	131910972	131910972	T	G	NM_020911:c.A1930C:p.M644L
ICC19	PLXNB2	Missense	chr22	50722116	50722116	C	A	NM_012401:c.G2485T:p.G829C
ICC10	PLXNB3	Nonframeshift	chrX	153032888	153032903	CCTG GCCA TCCG CCAG	C	NM_005393:c.606_621C:PLXNB3
ICC6	PODXL	Nonframeshift	chr7	131241029	131241035	GGGC GAC	G	NM_001018111:c.84_90C:PODXL
ICC10	POLQ	Missense	chr3	121248537	121248537	C	A	NM_199420:c.G1063T:p.D355Y
ICC16	POTEE	Silent	chr2	132021815	132021815	G	A	NM_001083538:c.G2787A:p.T929T
ICC23	POTEH	Missense	chr22	16287851	16287851	G	A	NM_001136213:c.C35T:p.S12F
ICC19	PPM1H	Missense	chr12	63042363	63042363	T	C	NM_020700:c.A1451G:p.D484G
ICC19	PPP1R16B	Nonsense	chr20	37536684	37536684	C	T	NM_001172735:c.C916T:p.R306X
ICC10	PPP2R1A	Nonsense	chr19	52729019	52729019	C	T	NM_014225:c.C1711T:p.Q571X
ICC25	PPP2R1B	Splicing	chr11	111608294	111608294	C	A	NM_181699:exon16:c.1790-1G > T,NM_181700:exon14:c.1598-1G > T
ICC23	PPP6R1	Silent	chr19	55748290	55748290	G	A	NM_014931:c.C1800T:p.D600D
ICC19	PQBP1	Silent	chrX	48759775	48759775	C	A	NM_001167992:c.C258A:p.P86P
ICC25	PRAMEF7, PRAMEF8	Silent	chr1	12979729	12979729	G	A	NM_001012277:c.G921A:p.S307S
ICC19	PRB1	Missense	chr12	11508477	11508477	A	G	NM_199353:c.T11C:p.I4T
ICC19	PRB2	Missense	chr12	11548453	11548453	A	G	NM_006248:c.T11C:p.I4T
ICC23	PRDX1	Silent	chr1	45977064	45977064	A	C	NM_001202431:c.T537G:p.P179P
ICC23	PREX2	Silent	chr8	69032463	69032463	G	A	NM_024870:c.G3537A:p.Q1179Q
ICC10	PRKAG2	Missense	chr7	151267290	151267290	A	C	NM_024429:c.T350G:p.F117C
ICC23	PRPH	Missense	chr12	49689301	49689301	C	A	NM_006262:c.C318A:p.D106E
ICC19	PRPS1L1	Missense	chr7	18066601	18066601	C	T	NM_175886:c.G805A:p.V269M
ICC30	PRRC2B	Missense	chr9	134322013	134322013	A	G	NM_013318:c.A839G:p.D280G
ICC10	PRRC2C	Missense	chr1	171556789	171556789	G	A	NM_015172:c.G7965A:p.M2655I
ICC10	PRUNE2	Missense	chr9	79320448	79320448	C	T	NM_015225:c.G6742A:p.G2248S
ICC26	PSD4	Missense	chr2	113943474	113943474	C	A	NM_012455:c.C1270A:p.P424T
ICC30	PSG8	Missense	chr19	43258694	43258694	C	T	NM_001130168:c.G668A:p.R223H
ICC26	PSKH1	Missense	chr16	67943151	67943151	C	T	NM_006742:c.C499T:p.R167W
ICC6	PSMG2	Silent	chr18	12706644	12706644	T	C	NM_020232:c.T153C:p.C51C
ICC16	PTPN14	Missense	chr1	214537983	214537983	T	C	NM_005401:c.A3307G:p.T1103A
ICC10	PTPN3	Nonsense	chr9	112200427	112200427	A	T	NM_001145369:c.T161A:p.L54X

ICC10	PTPRQ	Missense	chr12	80862515	80862515	G	A	NM_001145026:c.G421A:p.V141I
ICC6	PXDNL	Silent	chr8	52321280	52321280	G	A	NM_144651:c.C2904T:p.A968A
ICC6	PXDNL	Silent	chr8	52412309	52412309	T	C	NM_144651:c.A402G:p.L134L
ICC19	PYCRL	Missense	chr8	144688842	144688842	G	A	NM_023078:c.C380T:p.P127L
ICC29	PYCRL	Missense	chr8	144690251	144690251	C	T	NM_023078:c.G173A:p.R58K
ICC26	QRFPR	Missense	chr4	122250728	122250728	G	A	NM_198179:c.C1037T:p.A346V
ICC10	QRICH1	Missense	chr3	49095196	49095196	G	A	NM_198880:c.C437T:p.S146L
ICC23	RAB11FIP3	Missense	chr16	570781	570781	T	G	NM_001142272:c.T1325G:p.I442S
ICC23	RAB11FIP3	Missense	chr16	570790	570790	T	A	NM_001142272:c.T1334A:p.I445N
ICC26	RAD21L1	Silent	chr20	1212252	1212252	C	G	NM_001136566:c.C357G:p.T119T
ICC23	RAF1	Missense	chr3	12626443	12626443	T	G	NM_002880:c.A1706C:p.D569A
ICC16	RALGDS	Missense	chr9	135985719	135985719	G	A	NM_001042368:c.G287T:p.W96L
ICC30	RALGPS1	Nonsense	chr9	129958898	129958898	G	T	NM_014636:c.G1183T:p.G395X
ICC25	RAPGEF6	Silent	chr5	130857092	130857092	A	G	NM_001164386:c.T618C:p.D206D
ICC19	RAPH1	Frameshift	chr2	204305996	204305996	T	TG	RAPH1:NM_213589:exon14:c.1917_1917delinsCA
ICC23	RARS	Silent	chr5	167919852	167919852	G	A	NM_002887:c.G369A:p.Q123Q
ICC19	RB1	Splicing	chr13	48955580	48955580	G	C	NM_000321:exon17:c.1695+1G > C
ICC10	RBFOX1	Silent	chr16	7645562	7645562	C	T	NM_145891:c.C540T:p.F180F
ICC30	RBM15	Missense	chr1	110883546	110883546	T	G	NM_001201545:c.T1519G:p.W507G
ICC25	REPIN1	Frameshift	chr7	150069407	150069407	C	CG	NM_014374:c.1077_1077delinsCG:REPIN1
ICC29	RESP18	Missense	chr2	220197356	220197356	C	T	NM_001007089:c.G122A:p.G41E
ICC31	RETSAT	Missense	chr2	85570849	85570849	C	T	NM_017750:c.G1606A:p.G536R
ICC31	RETSAT	Missense	chr2	85570857	85570857	G	A	NM_017750:c.C1598T:p.A533V
ICC23	RGSL1	Silent	chr1	182491237	182491237	A	C	NM_001137669:c.A1924C:p.R642R
ICC16	RHBDF2	Nonsense	chr17	74469139	74469139	C	A	NM_001005498:c.G1858T:p.E620X
ICC31	RHOT2	Missense	chr16	721116	721116	G	A	NM_138769:c.G782A:p.R261H
ICC6	RIMBP2	Missense	chr12	130907079	130907079	G	A	NM_015347:c.C2389T:p.R797W
ICC26	RIMS1	Silent	chr6	72892743	72892743	C	G	NM_014989:c.C1569G:p.G523G
ICC26	RIPPLY2	Missense	chr6	84566997	84566997	A	T	NM_001009994:c.A276T:p.L92F
ICC16	RNF17	Silent	chr13	25433269	25433269	C	T	NM_001184993:c.C3729T:p.G1243G
ICC6	RNF183	Silent	chr9	116060297	116060297	G	A	NM_145051:c.C168T:p.C56C
ICC19	RNPS1	Missense	chr16	2305613	2305613	C	T	NM_006711:c.G791A:p.R264H
ICC19	ROBO1	Nonsense	chr3	78717389	78717389	G	C	NM_001145845:c.C1586G:p.S529X
ICC30	ROBO2	Silent	chr3	77607111	77607111	T	C	NM_002942:c.T1248C:p.P416P
ICC29	ROBO3	Missense	chr11	124738791	124738791	G	A	NM_022370:c.G254A:p.C85Y
ICC26	RPAP3	Missense	chr12	48064077	48064077	T	A	NM_001146075:c.A1237T:p.I413F
ICC26	RPL10	Splicing	chrX	153626737	153626737	T	G	
ICC29	RPL13A	Missense	chr19	49990891	49990891	C	A	NM_012423:c.C5A:p.A2E
ICC23	RPN2	Missense	chr20	35852297	35852297	C	G	NM_001135771:c.C1013G:p.S338C
ICC26	RPS13	Silent	chr11	17099183	17099183	A	G	NM_001017:c.T6C:p.G2G

ICC25	RSPH6A	Silent	chr19	46308005	46308005	C	T	NM_030785:c.G1158A:p.A386A
ICC29	RXRA	Missense	chr9	137321026	137321026	C	A	NM_002957:c.C983A:p.T328N
ICC30	SACS	Silent	chr13	23910620	23910620	C	T	NM_014363:c.G7395A:p.S2465S
ICC25	SAMD4B	Missense	chr19	39866452	39866452	C	T	NM_018028:c.C830T:p.A277V
ICC19	SAP30BP	Missense	chr17	73663541	73663541	C	T	NM_013260:c.C89T:p.A30V
ICC10	SASS6	Silent	chr1	100550923	100550923	C	T	NM_194292:c.G1935A:p.A645A
ICC25	SAV1	Splicing	chr14	51111461	51111461	C	T	NM_021818:exon4:c.806+1G > A
ICC26	SAV1	Splicing	chr14	51111461	51111461	C	T	NM_021818:exon4:c.806+1G > A
ICC23	SBK1	Missense	chr16	28331562	28331562	C	T	NM_001024401:c.C595T:p.R199C
ICC31	SCN10A	Silent	chr3	38763767	38763767	C	T	NM_006514:c.G3489A:p.L1163L
ICC29	SCN3A	Missense	chr2	166003373	166003373	T	A	NM_001081676:c.A1547T:p.N516I
ICC19	SCN5A	Missense	chr3	38592734	38592734	G	A	NM_001099405:c.C5075T:p.S1692L
ICC31	SCUBE1	Missense	chr22	43619154	43619154	C	T	NM_173050:c.G1276A:p.G426S
ICC10	SCUBE2	Nonframeshift	chr11	9113008	9113011	GGCA	G	NM_001170690:c.65_68C:SCUBE2
ICC30	SDK1	Missense	chr7	4273010	4273010	C	T	NM_001079653:c.C1412T:p.A471V
ICC29	SEC14L5	Splicing	chr16	5050927	5050927	G	A	NM_014692:exon10:c.1130+1G > A
ICC30	SEC63	Missense	chr6	108214751	108214751	T	C	NM_007214:c.A1609G:p.T537A
ICC10	SERPINA1	Silent	chr14	94847426	94847426	G	A	NM_000295:c.C699T:p.H233H
ICC23	SERPINA1	Silent	chr14	94847330	94847330	C	T	NM_000295:c.G795A:p.L265L
ICC10	SETBP1	Silent	chr18	42643234	42643234	C	T	NM_015559:c.C4362T:p.R1454R
ICC19	SETD2	Missense	chr3	47164030	47164030	T	A	NM_014159:c.A2096T:p.D699V
ICC23	SETDB1	Missense	chr1	150900299	150900299	A	G	NM_001145415:c.A109G:p.M37V
ICC29	SETDB1	Missense	chr1	150921872	150921872	G	A	NM_001145415:c.G1451A:p.R484Q
ICC23	SETX	Silent	chr9	135204225	135204225	A	T	NM_015046:c.T2760A:p.P920P
ICC25	SF3A1	Missense	chr22	30730681	30730681	T	C	NM_001005409:c.A2089G:p.I697V
ICC19	SF3B1	Missense	chr2	198269822	198269822	T	C	NM_012433:c.A1517G:p.N506S
ICC10	SH3BP4	Nonsense	chr2	235951091	235951091	C	T	NM_014521:c.C1678T:p.R560X
ICC10	SH3BP5	Missense	chr3	15297738	15297738	C	T	NM_001018009:c.G752A:p.S251N
ICC10	SHANK1	Missense	chr19	51205841	51205841	G	A	NM_016148:c.C1630T:p.R544C
ICC29	SHROOM4	Missense	chrX	50377806	50377806	G	T	NM_020717:c.C1267A:p.H423N
ICC23	SIGLEC15	Missense	chr18	43405695	43405695	T	C	NM_213602:c.T2C:p.M1T
ICC16	SLC12A1	Missense	chr15	48525034	48525034	A	C	NM_000338:c.A1086C:p.Q362H
ICC16	SLC13A5	Missense	chr17	6609969	6609969	C	A	NM_001143838:c.G360T:p.K120N
ICC10	SLC22A6	Missense	chr11	62748488	62748488	G	A	NM_004790:c.C1006T:p.R336C
ICC23	SLC28A1	Missense	chr15	85447335	85447335	G	T	NM_004213:c.G469T:p.A157S
ICC29	SLC30A3	Missense	chr2	27479278	27479278	C	T	NM_003459:c.G994A:p.V332I
ICC23	SLC36A4	Missense	chr11	92881877	92881877	T	C	NM_152313:c.A1341G:p.I447M
ICC10	SLC38A10	Missense	chr17	79226927	79226927	G	A	NM_001037984:c.C1402T:p.R468W
ICC30	SLC44A2	Missense	chr19	10747017	10747017	G	C	NM_001145056:c.G1246C:p.E416Q
ICC23	SLC46A2	Silent	chr9	115652833	115652833	C	T	NM_033051:c.G129A:p.V43V
ICC19	SLC6A13	Silent	chr12	330554	330554	G	A	NM_001190997:c.C1398T:p.G466G

ICC25	SLC7A11	Nonsense	chr4	139104428	139104428	G	C	NM_014331:c.C947G:p.S316X
ICC31	SLC7A11	Missense	chr4	139157563	139157563	G	C	NM_014331:c.C320G:p.S107C
ICC23	SMAD6	Missense	chr15	67073638	67073638	C	T	NM_001142861:c.C473T:p.A158V
ICC26	SMAD7	Missense	chr18	46468922	46468922	T	A	NM_001190823:c.A107T:p.D36V
ICC30	SMARCA4	Silent	chr19	11097100	11097100	C	T	NM_001128845:c.C591T:p.P197P
ICC6	SMARCC1	Silent	chr3	47719729	47719729	A	T	NM_003074:c.T1530A:p.A510A
ICC30	SNAP47	Silent	chr1	227935779	227935779	C	T	NM_053052:c.C477T:p.V159V
ICC29	SNTG2	Missense	chr2	1241752	1241752	C	A	NM_018968:c.C812A:p.A271E
ICC6	SNX1	Missense	chr15	64388225	64388225	G	T	NM_001242933:c.G13T:p.G5C
ICC16	SORD	Silent	chr15	45332635	45332635	A	G	NM_003104:c.A96G:p.P32P
ICC30	SOX9	Frameshift	chr17	70120344	70120344	G	GCT	SOX9:NM_000346:exon3:c.1346_1346delinsGCT
ICC26	SPDYE6	Missense	chr7	101988983	101988983	G	A	NM_001146210:c.C890T:p.P297L
ICC25	SPEG	Missense	chr2	220327027	220327027	T	G	NM_001173476:c.T77G:p.L26R
ICC6	SPEN	Missense	chr1	16257668	16257668	G	A	NM_015001:c.G4933A:p.V1645I
ICC6	SPG20	Missense	chr13	36878633	36878633	G	A	NM_001142294:c.C1870T:p.L624F
ICC25	SPRR3	Silent	chr1	152975739	152975739	T	A	NM_001097589:c.T243A:p.G81G
ICC30	SPRY2	Missense	chr13	80911584	80911584	G	C	NM_005842:c.C257G:p.P86R
ICC30	SPTBN2	Silent	chr11	66472485	66472485	G	A	NM_006946:c.C2262T:p.A754A
ICC31	SPZ1	Missense	chr5	79617117	79617117	C	G	NM_032567:c.C1083G:p.D361E
ICC19	SSX5	Missense	chrX	48054217	48054217	T	C	NM_175723:c.A143G:p.Y48C
ICC19	SSX5	Missense	chrX	48054218	48054218	A	T	NM_175723:c.T142A:p.Y48N
ICC23	SSX5	Missense	chrX	48049649	48049649	G	T	NM_175723:c.C386A:p.A129E
ICC30	ST6GALNAC5	Missense	chr1	77509964	77509964	C	A	NM_030965:c.C337A:p.Q113K
ICC23	STAT6	Missense	chr12	57496641	57496641	T	C	NM_001178081:c.A946G:p.I316V
ICC29	STIL	Missense	chr1	47755148	47755148	A	G	NM_001048166:c.T982C:p.F328L
ICC19	SULF1	Missense	chr8	70541824	70541824	C	T	NM_001128204:c.C2194T:p.R732W
ICC19	SULT1A2	Silent	chr16	28603714	28603714	C	T	NM_001054:c.G645A:p.L215L
ICC30	SUSD2	Silent	chr22	24579089	24579089	G	A	NM_019601:c.G141A:p.T47T
ICC23	SYNPR	Missense	chr3	63542364	63542364	G	A	NM_144642:c.G295A:p.V99I
ICC30	SYT3	Missense	chr19	51135883	51135883	C	T	NM_032298:c.G334A:p.G112R
ICC30	TADA2A	Missense	chr17	35837062	35837062	G	C	NM_001166105:c.G1307C:p.R436T
ICC16	TAF1	Missense	chrX	70604898	70604898	A	C	NM_004606:c.A2285C:p.Q762P
ICC23	TAF11	Missense	chr6	34855574	34855574	T	C	NM_001270488:c.A161G:p.E54G
ICC30	TAGLN2	Silent	chr1	159889554	159889554	G	A	NM_003564:c.C252T:p.T84T
ICC16	TANC2	Missense	chr17	61498648	61498648	C	A	NM_025185:c.C5305A:p.P1769T
ICC26	TBC1D17	Silent	chr19	50381459	50381459	A	T	NM_024682:c.A81T:p.R27R
ICC6	TBKBP1	Missense	chr17	45773695	45773695	G	A	NM_014726:c.G217A:p.E73K
ICC26	TBRG4	Missense	chr7	45148659	45148659	T	C	NM_001261834:c.A211G:p.K71E
ICC26	TCEB3C, TCEB3CL	Silent	chr18	44555098	44555098	G	A	NM_001100817:c.C1116T:p.P372P
ICC26	TCHH	Missense	chr1	152080367	152080367	C	T	NM_007113:c.G5326A:p.E1776K

ICC16	TCHHL1	Missense	chr1	152057844	152057844	A	G	NM_001008536:c.T2314C:p.S772P
ICC25	TECPR1	Silent	chr7	97857357	97857357	G	A	NM_015395:c.T2482T:p.W828W
ICC29	TECPR2	Missense	chr14	102904399	102904399	G	A	NM_001172631:c.G2435A:p.G812D
ICC16	TECTA	Missense	chr11	121016451	121016451	A	G	NM_005422:c.A3731G:p.Y1244C
ICC30	TENM4	Silent	chr11	78497995	78497995	G	A	NM_001098816:c.C2313T:p.R771R
ICC31	TENM4	Missense	chr11	78383149	78383149	C	T	NM_001098816:c.G5722A:p.D1908N
ICC30	TEP1	Silent	chr14	20859166	20859166	C	T	NM_007110:c.G2187A:p.L729L
ICC6	TGFBR1	Nonsense	chr9	101895007	101895007	C	G	NM_004612:c.C560G:p.S187X
ICC30	TGFBR2	Nonframeshift	chr3	30713543	30713546	GAGA	G	NM_003242:c.868_871G:TGFBR2
ICC30	TGFBR2	Missense	chr3	30691938	30691938	T	A	NM_003242:c.T440A:p.I147N
ICC30	TGM2	Missense	chr20	36793594	36793594	C	T	NM_004613:c.G7A:p.E3K
ICC23	TH1L	Missense	chr20	57568591	57568591	A	C	NM_198976:c.A1486C:p.M496L
ICC6	THSD4	Missense	chr15	72069691	72069691	C	T	NM_024817:c.C3035T:p.T1012M
ICC6	TICAM1	Missense	chr19	4817266	4817266	T	C	NM_182919:c.A1124G:p.H375R
ICC23	TIGD5	Missense	chr8	144681130	144681130	G	T	NM_032862:c.G1057T:p.A353S
ICC6	TIGIT	Missense	chr3	114014556	114014556	C	T	NM_173799:c.C226T:p.H76Y
ICC29	TKTL2	Missense	chr4	164393233	164393233	T	A	NM_032136:c.A1654T:p.S552C
ICC31	TLR3	Missense	chr4	187003955	187003955	G	T	NM_003265:c.G1115T:p.S372I
ICC6	TMCC3	Missense	chr12	94972226	94972226	C	T	NM_020698:c.G1075A:p.A359T
ICC19	TMEM119	Silent	chr12	108986085	108986085	G	T	NM_181724:c.C75A:p.A25A
ICC19	TMEM132B	Silent	chr12	126137013	126137013	C	T	NM_052907:c.C1926T:p.I642I
ICC6	TMEM143	Missense	chr19	48837390	48837390	G	T	NM_018273:c.C1040A:p.T347K
ICC25	TMEM14B	Missense	chr6	10756728	10756728	C	T	NM_001127711:c.C220T:p.R74C
ICC29	TMEM14B	Missense	chr6	10756728	10756728	C	T	NM_001127711:c.C220T:p.R74C
ICC30	TMEM184B	Missense	chr22	38643871	38643871	T	C	NM_001195071:c.A97G:p.T33A
ICC16	TMEM2	Silent	chr9	74315641	74315641	G	T	NM_001135820:c.C3105A:p.I1035I
ICC29	TMEM214	Missense	chr2	27262956	27262956	A	T	NM_001083590:c.A1546T:p.S516C
ICC19	TMEM63A	Silent	chr1	226034909	226034909	G	A	NM_014698:c.C2256T:p.Y752Y
ICC31	TMPRSS13	Nonframeshift	chr11	117789312	117789327	CGGG CTGG AGAT GCCT	C	NM_001077263:c.248_263G:TMPR SS13
ICC6	TMPRSS13	Nonframeshift	chr11	117789312	117789327	CGGG CTGG AGAT GCCT	C	NM_001077263:c.248_263G:TMPR SS13
ICC10	TMPRSS15	Missense	chr21	19685373	19685373	G	A	NM_002772:c.C2054T:p.T685M
ICC10	TNC	Missense	chr9	117853243	117853243	C	T	NM_002160:c.G55A:p.A19T
ICC10	TNFRSF10C	Silent	chr8	22974370	22974370	G	A	NM_003841:c.G606A:p.P202P
ICC19	TNFRSF13B	Missense	chr17	16852126	16852126	C	T	NM_012452:c.G371A:p.G124E
ICC16	TNFRSF21	Silent	chr6	47254023	47254023	A	G	NM_014452:c.T405C:p.P135P
ICC29	TNKS	Missense	chr8	9623838	9623838	G	T	NM_003747:c.G3643T:p.A1215S
ICC16	TNRC6A	Silent	chr16	24801883	24801883	A	T	NM_014494:c.A1920T:p.G640G

ICC26	TNRC6A	Missense	chr16	24801562	24801562	A	T	NM_014494:c.A1599T:p.K533N
ICC10	TP53	Missense	chr17	7578440	7578440	T	C	NM_000546:c.A490G:p.K164E
ICC16	TP53	Nonsense	chr17	7578263	7578263	G	A	NM_000546:c.C586T:p.R196X
ICC29	TP53	Missense	chr17	7578265	7578265	A	G	NM_000546:c.T584C:p.I195T
ICC16	TPTE	Missense	chr21	10934942	10934942	A	G	NM_199260:c.T737C:p.L246P
ICC26	TRAPPC8	Missense	chr18	29511309	29511309	T	A	NM_014939:c.A335T:p.Y112F
ICC29	TRIM14	Missense	chr9	100849984	100849984	C	T	NM_014788:c.G1097A:p.R366H
ICC16	TRIM23	Missense	chr5	64906836	64906836	C	T	NM_001656:c.G680A:p.R227Q
ICC26	TRIM24	Missense	chr7	138258340	138258340	G	C	NM_003852:c.G1865C:p.R622T
ICC16	TRIM36	Silent	chr5	114482814	114482814	T	G	NM_018700:c.A576C:p.I192I
ICC10	TRIM45	Silent	chr1	117661251	117661251	G	A	NM_001145635:c.C627T:p.P209P
ICC23	TRIM49	Missense	chr11	89531764	89531764	T	C	NM_020358:c.A893G:p.N298S
ICC26	TRIM49C	Missense	chr11	89774252	89774252	G	A	NM_001195234:c.G893A:p.S298N
ICC6	TRIM72	Silent	chr16	31235761	31235761	C	T	NM_001008274:c.C1119T:p.H373H
ICC19	TRPA1	Missense	chr8	72981272	72981272	T	C	NM_007332:c.A430G:p.N144D
ICC30	TRPA1	Silent	chr8	72951118	72951118	T	C	NM_007332:c.A2277G:p.S759S
ICC30	TRPC1	Missense	chr3	142499703	142499703	A	T	NM_003304:c.A690T:p.Q230H
ICC30	TRPC5	Silent	chrX	111195610	111195610	C	T	NM_012471:c.G39A:p.P13P
ICC16	TRPM1	Splicing	chr15	31321573	31321573	C	T	NM_002420:exon24:c.3082+1G > A
ICC25	TRRAP	Missense	chr7	98547196	98547196	G	T	NM_003496:c.G4870T:p.A1624S
ICC16	TSC22D2	Missense	chr3	150128962	150128962	A	G	NM_014779:c.A1825G:p.I609V
ICC23	TSGA13	Missense	chr7	130368467	130368467	T	C	NM_052933:c.A67G:p.K23E
ICC10	TSHZ3	Silent	chr19	31769874	31769874	C	T	NM_020856:c.G825A:p.L275L
ICC30	TSHZ3	Missense	chr19	31770589	31770589	G	C	NM_020856:c.C110G:p.A37G
ICC30	TSPAN14	Missense	chr10	82271948	82271948	T	G	NM_001128309:c.T130G:p.Y44D
ICC19	TTLL5	Silent	chr14	76259413	76259413	A	G	NM_015072:c.A3141G:p.P1047P
ICC25	TTLL9	Missense	chr20	30497592	30497592	T	A	NM_001008409:c.T371A:p.L124Q
ICC16	TTN	Missense	chr2	179614790	179614790	T	C	NM_133379:c.A12337G:p.I4113V
ICC6	TTN	Missense	chr2	179579256	179579256	C	T	NM_133378:c.G22513A:p.V7505I
ICC30	TUBGCP6	Silent	chr22	50656933	50656933	G	A	NM_020461:c.C4938T:p.F1646F
ICC23	TULP4	Missense	chr6	158925125	158925125	C	A	NM_020245:c.C4430A:p.A1477D
ICC19	TYR	Missense	chr11	88911782	88911782	G	A	NM_000372:c.G661A:p.E221K
ICC16	UBASH3A	Missense	chr21	43867189	43867189	T	C	NM_001243467:c.T1540C:p.S514P
ICC23	UBE3A	Missense	chr15	25616683	25616683	T	C	NM_130838:c.A578G:p.D193G
ICC23	UBP1	Silent	chr3	33454317	33454317	G	A	NM_001128160:c.C345T:p.S115S
ICC16	UBQLN3	Missense	chr11	5528966	5528966	T	A	NM_017481:c.A1823T:p.Q608L
ICC10	UBR4	Frameshift	chr1	19518780	19518780	C	CT	UBR4:NM_020765:exon11:c.1296_1296delinsAG
ICC26	UBR5	Missense	chr8	103338791	103338791	T	C	NM_015902:c.A1582G:p.I528V
ICC16	UBXN1	Missense	chr11	62445991	62445991	A	C	NM_015853:c.T196G:p.S66A
ICC6	UMPS	Silent	chr3	124459010	124459010	C	T	NM_000373:c.C1122T:p.T374T

ICC19	UNC80	Missense	chr2	210786335	210786335	A	G	NM_032504:c.A5257G:p.I1753V
ICC16	URGCP	Missense	chr7	43916423	43916423	C	T	NM_017920:c.G2612A:p.G871D
ICC29	USP43	Missense	chr17	9586239	9586239	T	A	NM_001267576:c.T1205A:p.F402Y
ICC6	USP6	Missense	chr17	5037195	5037195	G	A	NM_004505:c.G398A:p.R133K
ICC30	UTS2R	Silent	chr17	80333142	80333142	C	T	NM_018949:c.C942T:p.L314L
ICC16	VAT1L	Missense	chr16	77859249	77859249	A	T	NM_020927:c.A470T:p.E157V
ICC16	VCAN	Missense	chr5	82815194	82815194	A	G	NM_001164098:c.A1069G:p.S357G
ICC26	VCPIP1	Frameshift	chr8	67577959	67577963	CTAAG	C	VCPIP1:NM_025054:exon1:c.1231_1235G
ICC25	VCX3A	Missense	chrX	6451869	6451869	C	T	NM_016379:c.G478A:p.V160M
ICC25	VCX3A	Silent	chrX	6451864	6451864	C	T	NM_016379:c.G483A:p.E161E
ICC6	VCX3A	Missense	chrX	6451869	6451869	C	T	NM_016379:c.G478A:p.V160M
ICC6	VCX3A	Silent	chrX	6451864	6451864	C	T	NM_016379:c.G483A:p.E161E
ICC25	VEPH1	Silent	chr3	157081319	157081319	T	C	NM_001167911:c.A1569G:p.E523E
ICC10	VGLL4	Missense	chr3	11600049	11600049	G	T	NM_001128220:c.C614A:p.S205Y
ICC19	VPRBP	Silent	chr3	51457718	51457718	G	C	NM_001171904:c.C2544G:p.T848T
ICC19	VPS13B	Missense	chr8	100168814	100168814	C	G	NM_015243:c.C2051G:p.S684C
ICC10	VSIG10L	Silent	chr19	51840598	51840598	G	A	NM_001163922:c.C2199T:p.A733A
ICC16	VWA3A	Silent	chr16	22163874	22163874	C	T	NM_173615:c.C3324T:p.G1108G
ICC31	VWA3A	Missense	chr16	22157632	22157632	C	T	NM_173615:c.C2806T:p.R936C
ICC30	VWF	Silent	chr12	6166219	6166219	C	T	NM_000552:c.G1749A:p.A583A
ICC25	WDR35	Silent	chr2	20178570	20178570	G	A	NM_001006657:c.C378T:p.D126D
ICC31	WDR49	Missense	chr3	167218053	167218053	T	A	NM_178824:c.A1863T:p.E621D
ICC6	WDR66	Silent	chr12	122396424	122396424	C	T	NM_001178003:c.C1977T:p.P659P
ICC30	WDR72	Silent	chr15	53992011	53992011	C	T	NM_182758:c.G1701A:p.P567P
ICC30	WDR82	Silent	chr3	52312327	52312327	G	A	NM_025222:c.C51T:p.F17F
ICC16	WISP1	Missense	chr8	134237728	134237728	A	T	NM_080838:c.A445T:p.T149S
ICC30	XIRP2	Missense	chr2	168104342	168104342	C	G	NM_001199144:c.C5774G:p.T1925S
ICC16	XPO1	Missense	chr2	61749770	61749770	T	C	NM_003400:c.A277G:p.I93V
ICC6	XRN1	Silent	chr3	142051367	142051367	G	T	NM_001042604:c.C4069A:p.R1357R
ICC29	YBX1	Missense	chr1	43166630	43166630	G	A	NM_004559:c.G919A:p.D307N
ICC19	ZCCHC11	Silent	chr1	52933893	52933893	A	G	NM_001009881:c.T2925C:p.I975I
ICC25	ZDHHC11	Missense	chr5	825293	825293	C	G	NM_024786:c.G1009C:p.D337H
ICC23	ZDHHC21	Missense	chr9	14662273	14662273	C	T	NM_178566:c.G305A:p.R102H
ICC23	ZER1	Missense	chr9	131515103	131515103	G	C	NM_006336:c.C772G:p.L258V
ICC26	ZFHX3	Nonframeshift	chr16	72821642	72821642	A	ACCG CCG	NM_001164766:c.7791_7791delinsCGGCGGT:ZFHX3
ICC26	ZFHX3	Silent	chr16	72821618	72821618	A	G	NM_001164766:c.T7815C:p.G2605G
ICC29	ZFHX3	Silent	chr16	72821618	72821618	A	G	NM_001164766:c.T7815C:p.G2605G
ICC23	ZFP64	Missense	chr20	50701509	50701509	C	T	NM_199427:c.G1525A:p.A509T
ICC29	ZFR	Missense	chr5	32380242	32380242	A	G	NM_016107:c.T2678C:p.V893A
ICC30	ZFR	Missense	chr5	32395347	32395347	C	T	NM_016107:c.G1897A:p.E633K

ICC30	ZFYVE26	Missense	chr14	68264958	68264958	C	A	NM_015346:c.G2021T:p.G674V
ICC31	ZGPAT	Missense	chr20	62366114	62366114	A	T	NM_001083113:c.A929T:p.E310V
ICC30	ZIC4	Silent	chr3	147113919	147113919	G	A	NM_001168378:c.C558T:p.D186D
ICC6	ZIC5	Nonsense	chr13	100622544	100622544	C	T	NM_033132:c.G1386A:p.W462X
ICC16	ZMAT3	Missense	chr3	178785314	178785314	T	A	NM_022470:c.A227T:p.N76I
ICC31	ZNF224	Missense	chr19	44611089	44611089	C	G	NM_013398:c.C776G:p.P259R
ICC10	ZNF257	Missense	chr19	22271643	22271643	T	A	NM_033468:c.T1091A:p.I364K
ICC6	ZNF326	Silent	chr1	90493104	90493104	C	T	NM_182976:c.C1593T:p.G531G
ICC29	ZNF343	Silent	chr20	2465001	2465001	T	A	NM_024325:c.A606T:p.A202A
ICC30	ZNF426	Frameshift	chr19	9639144	9639146	TGA	T	ZNF426:NM_024106:exon8:c.1575_1577A
ICC23	ZNF467	Missense	chr7	149462429	149462429	C	T	NM_207336:c.G1162A:p.E388K
ICC23	ZNF479	Silent	chr7	57188855	57188855	C	T	NM_033273:c.G267A:p.T89T
ICC10	ZNF554	Silent	chr19	2834595	2834595	C	T	NM_001102651:c.C1362T:p.H454H
ICC29	ZNF592	Missense	chr15	85334000	85334000	A	T	NM_014630:c.A2285T:p.H762L
ICC19	ZNF613	Missense	chr19	52448430	52448430	G	C	NM_001031721:c.G1294C:p.E432Q
ICC10	ZNF616	Missense	chr19	52619071	52619071	T	G	NM_178523:c.A1346C:p.H449P
ICC16	ZNF681	Silent	chr19	23926705	23926705	A	G	NM_138286:c.T1647C:p.L549L
ICC26	ZNF687	Missense	chr1	151259929	151259929	G	A	NM_020832:c.G1162A:p.V388I
ICC16	ZNF700	Missense	chr19	12059912	12059912	G	C	NM_144566:c.G1073C:p.G358A
ICC30	ZNF701	Silent	chr19	53085819	53085819	C	T	NM_018260:c.C507T:p.N169N
ICC29	ZNF703	Nonframeshift	chr8	37555933	37555942	GCGCCGCCGC	G	ZNF703:NM_025069:exon2:c.1514_1523G
ICC26	ZNF729	Missense	chr19	22498959	22498959	G	A	NM_001242680:c.G2740A:p.G914S
ICC6	ZNF786	Missense	chr7	148768873	148768873	C	T	NM_152411:c.G991A:p.G331R
ICC19	ZNF804A	Missense	chr2	185800953	185800953	C	T	NM_194250:c.C830T:p.S277F
ICC26	ZNF804B	Missense	chr7	88965331	88965331	A	G	NM_181646:c.A3035G:p.N1012S
ICC23	ZNF814	Nonframeshift	chr19	58385869	58385869	C	CATA	ZNF814:NM_001144989:exon3:c.889_889delinsTATG
ICC29	ZNF835	Missense	chr19	57175341	57175341	T	A	NM_001005850:c.A1226T:p.Q409L
ICC29	ZRANB1	Silent	chr10	126631272	126631272	T	A	NM_017580:c.T210A:p.S70S
ICC26	ZSCAN21	Nonsense	chr7	99661707	99661707	A	T	NM_145914:c.A889T:p.R297X
ICC23	ZSWIM1	Missense	chr20	44512264	44512264	G	A	NM_080603:c.G1033A:p.E345K
ICC6	ZSWIM2	Missense	chr2	187698723	187698723	C	T	NM_182521:c.G778A:p.E260K

A total of 874 somatic variants of point mutations and indels are listed. Category and Description entries are as available from ANNOVAR output. Genomic coordinates (Chr-Start-End) are shown in accordance with hg19.

Supplementary Table S2: The description of WES data

Samples*	Sequencing reads**	Mapped (%)	Coverage (mean)	Coverage (median)	% of bases (> = 20 reads)
ICC-6N	61,418,697	60,967,388 (99.3%)	68.69	60	83.3%
ICC-6T	59,862,012	59,416,250 (99.3%)	66.01	58	83.4%
ICC-10N	69,422,056	68,895,772 (99.2%)	78.49	70	85.7%
ICC-10T	68,443,175	67,977,794 (99.3%)	77.16	65	83.9%
ICC-16N	69,914,613	69,467,729 (99.4%)	78.04	69	85.2%
ICC-16T	69,005,839	68,554,949 (99.3%)	77.06	66	84.6%
ICC-19N	67,620,768	67,107,891 (99.2%)	74.99	64	84.5%
ICC-19T	75,901,954	75,408,574 (99.3%)	85.42	73	85.5%
ICC-23N	68,505,596	68,039,715 (99.3%)	81.42	69	81.2%
ICC-23T	74,893,898	74,370,466 (99.3%)	83.05	72	86.2%
ICC-25N	66,858,895	66,308,854 (99.2%)	74.47	64	84.8%
ICC-25T	72,026,878	71,518,474 (99.3%)	78.58	67	85.3%
ICC-26N	66,838,463	66,373,284 (99.3%)	74.84	64	84.2%
ICC-26T	62,380,218	61,984,141 (99.4%)	70.77	59	82.4%
ICC-29N	70,946,000	70,439,091 (99.3%)	80.33	68	85.4%
ICC-29T	68,791,465	68,363,696 (99.4%)	77.68	68	85.1%
ICC-30N	60,616,351	60,179,140 (99.3%)	67.9	59	83.6%
ICC-30T	70,105,055	69,662,544 (99.4%)	78.82	68	85.2%
ICC-31N	62,619,020	62,205,258 (99.3%)	69.34	61	84.0%
ICC-31T	60,392,446	59,974,386 (99.3%)	66.78	59	83.3%

*The tumor and matched normal genomes are discriminated with the use of 'T' and 'N', respectively.

**The mean and median coverage as well as the % of bases (> = 20 reads) were calculated onto the targeted regions (Agilent SureSelect 50Mb exon).

Supplementary Table S3: Focal recurrent deletions of ICC

Cytobands	FDR	Chr	Start	End	Size (Mb)	Genes	Cancer genes
1p36.11	0.159	chr1	1	31905769	31.9	507	*CASP9, RUNX3, MTOR*
3p12.2	0.159	chr3	72799350	93593023	20.79	33	*ROBO1, ROBO2*
9p21.3	0.159	chr9	19895722	27289560	7.39	48	*CDKN2A, CDKN2B*
13q21.32	0.159	chr13	60706917	79945865	19.23	37	
18q21.2	0.159	chr18	46005343	59958610	13.95	74	*SMAD4*
12q21.33	0.174	chr12	69633297	132237633	62.6	447	*ATXN2, PTPN11, PTPRB*

GISTIC output of six focal deletions are shown.

Supplementary Table S4: RNA-seq somatic variants

Case	Symbol	Category	Chr	Start	End	Ref	Obs	Description
ICC10	SEPT6	Frameshift	chrX	118750704	118750704	A	AA	SEPT6:NM_145802:exon10:c.1282_1282delinsTT
ICC4	SEPT6	Silent	chr17	75478344	75478344	G	A	SEPT9:NM_001113496:exon2:c.G87A:p.G29G
ICC10	AADAT	Missense	chr4	171009629	171009629	C	T	AADAT:NM_016228:exon2:c.G154A:p.V52I
ICC16	ABCA5	Frameshift	chr17	67300835	67300835	A	AA	ABCA5:NM_172232:exon7:c.905_905delinsTT
ICC11	ABCC3	Missense	chr17	48745353	48745353	A	G	ABCC3:NM_003786:exon13:c.A1765G:p.I589V
ICC10	ACRC	Missense	chrX	70828888	70828888	G	A	ACRC:NM_052957:exon10:c.G1532A:p.G511E
ICC8	ACSL3	Missense	chr2	223781098	223781098	T	G	ACSL3:NM_203372:exon4:c.T440G:p.F147C
ICC6	ACSM2B	Splicing	chr16	20548686	20548686	T	C	ACSM2B(NM_182617:exon16:c.1630-2A > G
ICC3	ADAM15	Missense	chr1	155034741	155034741	C	T	ADAM15:NM_207191:exon19:c.C2228T:p.P743L
ICC8	ADAM17	Missense	chr2	9645454	9645454	A	G	ADAM17:NM_003183:exon12:c.T1385C:p.I462T
ICC16	ADAMTS1	Missense	chr21	28210104	28210104	T	C	ADAMTS1:NM_006988:exon9:c.A2698G:p.S900G
ICC15	ADAMTSL4	Silent	chr1	150531552	150531552	C	G	ADAMTSL4:NM_019032:exon16:c.G2674G:p.E892E
ICC15	ADCY3	Missense	chr2	25141324	25141324	G	C	ADCY3:NM_004036:exon1:c.C533G:p.S178C
ICC6	ADIPOR1	Missense	chr1	202914279	202914279	A	C	ADIPOR1:NM_015999:exon5:c.T449G:p.F150C
ICC8	ADIPOR2	Missense	chr12	1863585	1863585	C	G	ADIPOR2:NM_024551:exon2:c.C76G:p.Q26E
ICC16	AGAP4	Missense	chr10	46321745	46321745	C	A	AGAP4:NM_133446:exon7:c.G1610T:p.R537M
ICC16	AGBL2	Frameshift	chr11	47721031	47721031	T	TT	AGBL2:NM_024783:exon8:c.661_661delinsAA
ICC10	AHCTF1	Missense	chr1	247013960	247013960	G	C	AHCTF1:NM_015446:exon33:c.C5375G:p.S1792C
ICC16	AHR	Silent	chr7	17379819	17379819	A	T	AHR:NM_001621:exon10:c.A2370T:p.P790P
ICC14	AKR7A2	Silent	chr1	19630776	19630776	A	G	AKR7A2:NM_003689:exon7:c.T1023C:p.D341D
ICC8	ALG10	Frameshift	chr12	34179314	34179314	T	TT	ALG10:NM_032834:exon3:c.886_886delinsTT
ICC4	ALPK1	Missense	chr4	113353694	113353694	G	C	ALPK1:NM_001102406:exon11:c.G2991C:p.E997D
ICC8	ANKRD17	Missense	chr4	73944497	73944497	G	C	ANKRD17:NM_198889:exon30:c.C6517G:p.P2173A
ICC8	ANKRD27	Missense	chr19	33090910	33090910	C	G	ANKRD27:NM_032139:exon27:c.G2814C:p.Q938H
ICC5	ANXA4	Splicing	chr2	70037724	70037724	A	G	ANXA4(NM_001153:exon7:c.398-2A > G)

ICC8	AP3D1	Missense	chr19	2115319	2115319	C	G	AP3D1:NM_003938:exon20:c.G2248C:p.E750Q
ICC8	APH1B	Silent	chr15	63579732	63579732	C	T	APH1B:NM_031301:exon4:c.C465T:p.F155F
ICC8	AQR	Missense	chr15	35182486	35182486	T	C	AQR:NM_014691:exon24:c.A2611G:p.I871V
ICC3	ARAP2	Frameshift	chr4	36162165	36162165	T	TT	ARAP2:NM_015230:exon13:c.2358_2358delinsAA
ICC16	ARHGAP1	Missense	chr11	46717291	46717291	T	A	ARHGAP1:NM_004308:exon3:c.A148T:p.S50C
ICC16	ARHGAP39	Silent	chr8	145759525	145759525	G	A	ARHGAP39:NM_025251:exon9:c.C2676T:p.T892T
ICC10	ARHGAP5	Nonsense	chr14	32562474	32562474	C	T	ARHGAP5:NM_001030055:exon2:c.C2599T:p.R867X
ICC4	ARHGAP8	Missense	chr22	45204936	45204936	A	G	ARHGAP8:NM_001017526:exon5:c.A350G:p.K117R
ICC15	ARHGEF35	Missense	chr7	143885454	143885454	T	C	ARHGEF35:NM_001003702:exon2:c.A23G:p.H8R
ICC8	ARID1A	Frameshift	chr1	27101017	27101017	T	TT	ARID1A:NM_006015:exon18:c.4299_4299delinsTT
ICC11	ARL6IP5	Missense	chr3	69153765	69153765	A	G	ARL6IP5:NM_006407:exon3:c.A545G:p.Y182C
ICC11	ARMCX4	Missense	chrX	100749643	100749643	G	A	ARMCX4:NM_001256155:exon2:c.G6067A:p.E2023K
ICC8	ARMCX4	Frameshift	chrX	100749649	100749649	A	AA	ARMCX4:NM_001256155:exon2:c.6073_6073delinsAA
ICC8	ARRDC1	Missense	chr9	140509144	140509144	C	G	ARRDC1:NM_152285:exon7:c.C929G:p.S310C
ICC11	ATAD3A	Silent	chr1	1447756	1447756	G	C	ATAD3A:NM_001170535:exon1:c.G108C:p.G36G
ICC4	ATF6	Missense	chr1	161821600	161821600	C	A	ATF6:NM_007348:exon11:c.C1408A:p.L470I
ICC10	ATN1	Missense	chr12	7043435	7043435	A	C	ATN1:NM_001007026:exon3:c.A124C:p.S42R
ICC6	ATP13A2	Missense	chr1	17312829	17312829	C	T	ATP13A2:NM_001141973:exon29:c.G3415A:p.A1139T
ICC8	ATP2C1	Missense	chr3	130698226	130698226	G	A	ATP2C1:NM_001199181:exon18:c.G1806A:p.M602I
ICC10	ATP6V0C	Silent	chr16	2569608	2569608	C	T	ATP6V0C:NM_001694:exon3:c.C330T:p.I110I
ICC4	ATP6V0E1	Silent	chr5	172447277	172447277	C	G	ATP6V0E1:NM_003945:exon3:c.C189G:p.L63L
ICC14	ATRX	Frameshift	chrX	76855024	76855024	T	TT	ATRX:NM_138270:exon24:c.5698_5698delinsAA
ICC16	B3GNT9	Missense	chr16	67183620	67183620	C	T	B3GNT9:NM_033309:exon2:c.G769A:p.V257I
ICC8	BANP	Missense	chr16	88014663	88014663	T	C	BANP:NM_079837:exon3:c.T92C:p.V31A
ICC4	BAZ2B	Silent	chr2	160176839	160176839	T	C	BAZ2B:NM_013450:exon37:c.A6444G:p.R2148R
ICC4	BBS7	Missense	chr4	122782809	122782809	G	A	BBS7:NM_176824:exon4:c.C191T:p.P64L

ICC8	BCL9L	Missense	chr11	118771664	118771664	C	G	BCL9L:NM_182557:exon6:c.G2788C:p.G930R
ICC10	BRAT1	Missense	chr7	2581777	2581777	G	A	BRAT1:NM_152743:exon7:c.C992T:p.T331M
ICC14	C11orf80	Silent	chr11	66523921	66523921	A	G	C11orf80:NM_024650:exon3:c.A414G:p.S138S
ICC8	C19orf44	Missense	chr19	16612294	16612294	G	A	C19orf44:NM_032207:exon2:c.G691A:p.E231K
ICC16	C22orf29	Nonsense	chr22	19838797	19838797	C	A	C22orf29:NM_024627:exon3:c.G988T:p.E330X
ICC8	C8orf33	Missense	chr8	146279439	146279439	G	T	C8orf33:NM_023080:exon5:c.G586T:p.A196S
ICC8	C9orf9	Missense	chr9	135763745	135763745	G	T	C9orf9:NM_018956:exon4:c.G416T:p.S139I
ICC15	CAPN3	Splicing	chr15	42698123	42698123	G	A	CAPN3(NM_000070:exon15:c.1783-1G > A
ICC3	CAPN3	Silent	chr15	42698129	42698129	G	A	CAPN3:NM_173088:exon4:c.G252A:p.K84K
ICC16	CAPN9	Missense	chr1	230937323	230937323	A	G	CAPN9:NM_016452:exon19:c.A1976G:p.H659R
ICC6	CARM1	Missense	chr19	11018786	11018786	C	T	CARM1:NM_199141:exon3:c.C418T:p.R140W
ICC10	CASP8AP2	Missense	chr6	90577760	90577760	A	G	CASP8AP2:NM_001137667:exon8:c.A4751G:p.Y1584C
ICC15	CBWD6	Missense	chr9	69256833	69256833	A	G	CBWD6:NM_001085457:exon3:c.T298C:p.W100R
ICC8	CC2D2A	Missense	chr4	15529184	15529184	A	C	CC2D2A:NM_001080522:exon13:c.A1264C:p.S422R
ICC10	CCDC120	Missense	chrX	48925467	48925467	C	G	CCDC120:NM_001163321:exon10:c.C1817G:p.A606G
ICC16	CCDC180	Nonframeshift	chr9	100092983	100092983	G	GGAG GAG	CCDC180:NM_020893:exon18:c.2340_2340delinsGGAGGAG
ICC8	CCDC40	Missense	chr17	78021101	78021101	G	T	CCDC40:NM_017950:exon4:c.G576T:p.Q192H
ICC8	CCDC6	Missense	chr10	61572438	61572438	C	T	CCDC6:NM_005436:exon5:c.G802A:p.E268K
ICC15	CCDC74A	Missense	chr2	132289293	132289293	T	C	CCDC74A:NM_138770:exon4:c.T601C:p.S201P
ICC14	CD6	Missense	chr11	60775067	60775067	A	G	CD6:NM_001254750:exon3:c.A154G:p.S52G
ICC16	CDCP1	Nonsense	chr3	45152234	45152234	C	T	CDCP1:NM_022842:exon4:c.G755A:p.W252X
ICC4	CDHR2	Silent	chr5	176011850	176011850	C	A	CDHR2:NM_017675:exon19:c.C2568A:p.V856V
ICC3	CDK16	Missense	chrX	47085744	47085744	C	G	CDK16:NM_006201:exon9:c.C859G:p.L287V
ICC16	CENPC	Missense	chr4	68357909	68357909	A	C	CENPC:NM_001812:exon16:c.T2504G:p.I835S
ICC4	CEP95	Missense	chr17	62529257	62529257	A	C	CEP95:NM_138363:exon16:c.A1865C:p.H622P
ICC15	CERS6	Missense	chr2	169622200	169622200	A	G	CERS6:NM_001256126:exon9:c.A944G:p.N315S

ICC6	CES2	Missense	chr16	66969362	66969362	C	G	CES2:NM_198061:exon1:c.C16G:p.R6G
ICC5	CHD9	Frameshift	chr16	53243685	53243685	A	AA	CHD9:NM_025134:exon3:c.1744_1744del insAA
ICC8	CHMP4C	Silent	chr8	82670505	82670505	G	A	CHMP4C:NM_152284:exon4:c.G612A:p. S204S
ICC11	CLASP2	Missense	chr3	33576835	33576835	G	A	CLASP2:NM_015097:exon34:c.C3704T:p. P1235L
ICC8	CNKSR3	Nonsense	chr6	154731495	154731495	G	A	CNKSR3:NM_173515:exon12:c.C1351T:p. R451X
ICC15	CNTNAP3	Silent	chr9	39078814	39078814	C	A	CNTNAP3:NM_033655:exon22:c.G3546T:p. A1182A
ICC6	COL4A6	Silent	chrX	107413930	107413930	G	A	COL4A6:NM_033641:exon35:c.G3402T:p. R1134R
ICC8	CPS1	Nonframeshift	chr2	211421454	211421454	C	CTTC	CPS1:NM_001122633:exon2:c.15_15delin sCTTC
ICC4	CREBBP	Silent	chr16	3778409	3778409	C	T	CREBBP:NM_004380:exon31:c.G6639A:p. Q2213Q
ICC6	CSDE1	Missense	chr1	115261316	115261316	C	T	CSDE1:NM_001130523:exon19:c. G2312A:p.R771H
ICC3	CTAGE15	Nonframeshift	chr7	143269075	143269078	TCTC	T	CTAGE15:NM_001008747:exon1:c.165_1 68T
ICC4	CTAGE4	Missense	chr7	143882454	143882454	A	G	CTAGE4:NM_198495:exon1:c.A1858G:p. T620A
ICC8	CTNS	Missense	chr17	3558308	3558308	G	C	CTNS:NM_001031681:exon6:c.G242C:p. G81A
ICC11	CYP2C19	Missense	chr10	96609793	96609793	C	A	CYP2C19:NM_000769:exon8:c.C1269A:p. N423K
ICC16	CYP4A22	Missense	chr1	47610065	47610065	A	C	CYP4A22:NM_001010969:exon7:c. A827C:p.K276T
ICC15	CYTH3	Missense	chr7	6217518	6217518	C	A	CYTH3:NM_004227:exon5:c.G304T:p. A102S
ICC14	DAXX	Frameshift	chr6	33288235	33288235	T	TT	DAXX:NM_001350:exon4:c.1173_1173de linsAA
ICC10	DDX11	Silent	chr12	31242399	31242399	T	C	DDX11:NM_152438:exon8:c.T855C:p. C285C
ICC4	DDX11	Silent	chr12	31242861	31242861	A	C	DDX11:NM_152438:exon9:c.A922C:p. R308R
ICC6	DDX11	Missense	chr12	31244689	31244689	G	A	DDX11:NM_152438:exon10:c.G1126A:p. A376T
ICC6	DDX11	Silent	chr12	31244706	31244706	G	A	DDX11:NM_152438:exon10:c.G1143A:p. A381A
ICC15	DDX54	Missense	chr12	113603656	113603656	C	G	DDX54:NM_001111322:exon13:c. G1596C:p.K532N
ICC11	DHX58	Missense	chr17	40263868	40263868	G	C	DHX58:NM_024119:exon3:c.C43G:p.L15V
ICC8	DHX9	Missense	chr1	182827728	182827728	C	G	DHX9:NM_001357:exon9:c.C853G:p. Q285E
ICC3	DIS3L	Missense	chr15	66606416	66606416	G	A	DIS3L:NM_001143688:exon6:c.G775A:p. A259T
ICC15	DKC1	Missense	chrX	154003525	154003525	G	A	DKC1:NM_001142463:exon13:c.G1300A:p. A434T

ICC11	DLAT	Missense	chr11	111916633	111916633	A	G	DLAT:NM_001931:exon10:c.A1337G:p.Y446C
ICC15	DNAH1	Missense	chr3	52409985	52409985	G	A	DNAH1:NM_015512:exon46:c.G7174A:p.E2392K
ICC16	DNAJC9	Missense	chr10	75006474	75006474	T	A	DNAJC9:NM_015190:exon2:c.A293T:p.E98V
ICC10	DNM1L	Missense	chr12	32832335	32832335	A	T	DNM1L:NM_012062:exon1:c.A38T:p.D13V
ICC8	DUOXA2	Silent	chr15	45408784	45408784	T	C	DUOXA2:NM_207581:exon4:c.T411C:p.N137N
ICC3	DYNC2LI1	Splicing	chr2	44027978	44027979	AG	A	
ICC4	DYRK1A	Missense	chr21	38865352	38865352	T	A	DYRK1A:NM_130438:exon7:c.T985A:p.S329T
ICC11	DZIP3	Frameshift	chr3	108363324	108363324	A	AA	DZIP3:NM_014648:exon14:c.1455_1455delinsAA
ICC10	ECHDC2	Nonsense	chr1	53370437	53370437	G	A	ECHDC2:NM_018281:exon6:c.C490T:p.R164X
ICC8	EEF1D	Missense	chr8	144672086	144672086	C	T	EEF1D:NM_001130053:exon3:c.G166A:p.D56N
ICC8	EFCAB4B	Missense	chr12	3806059	3806059	A	G	EFCAB4B:NM_032680:exon4:c.T107C:p.L36P
ICC15	EHD2	Silent	chr19	48220010	48220010	G	T	EHD2:NM_014601:exon2:c.G141T:p.S47S
ICC10	EIF1AD	Missense	chr11	65766846	65766846	C	A	EIF1AD:NM_001242482:exon5:c.G328T:p.A110S
ICC10	EIF1AD	Missense	chr11	65766845	65766845	G	A	EIF1AD:NM_001242482:exon5:c.C329T:p.A110V
ICC4	EIF3C	Missense	chr16	28403381	28403381	T	C	EIF3C:NM_001199142:exon9:c.A859G:p.K287E
ICC4	EIF3CL	Missense	chr16	28734567	28734567	A	G	EIF3CL:NM_001099661:exon9:c.A859G:p.K287E
ICC10	ELFN2	Silent	chr22	37770495	37770495	G	A	ELFN2:NM_052906:exon3:c.C1080T:p.C360C
ICC3	EPG5	Frameshift	chr18	43490676	43490676	A	AA	EPG5:NM_020964:exon23:c.4015_4015delinsTT
ICC15	EPN3	Missense	chr17	48614260	48614260	G	C	EPN3:NM_017957:exon2:c.G343C:p.D115H
ICC3	ERAP2	Silent	chr5	96237326	96237326	G	A	ERAP2:NM_022350:exon11:c.G1689A:p.Q563Q
ICC3	F8	Frameshift	chrX	154157693	154157693	T	TT	F8:NM_000132:exon14:c.4372_4372delinsAA
ICC8	FAIM	Missense	chr3	138341144	138341144	G	C	FAIM:NM_001033030:exon4:c.G328C:p.E110Q
ICC8	FAM109A	Silent	chr12	111800641	111800641	G	A	FAM109A:NM_144671:exon3:c.C591T:p.F197F
ICC8	FAM177A1	Silent	chr14	35515783	35515783	C	G	FAM177A1:NM_173607:exon1:c.C114G:p.V38V
ICC16	FAM179B	Missense	chr14	45497475	45497475	G	A	FAM179B:NM_015091:exon10:c.G3601A:p.E1201K
ICC3	FAM179B	Missense	chr14	45432121	45432121	A	G	FAM179B:NM_015091:exon1:c.A497G:p.Q166R
ICC8	FAM193B	Missense	chr5	176959575	176959575	C	T	FAM193B:NM_001190946:exon5:c.G1144A:p.E382K

ICC14	FAM208B	Frameshift	chr10	5788560	5788560	A	AA	FAM208B:NM_017782:exon15:c.3176_3176delinsAA
ICC11	FAM47E	Splicing	chr4	77204532	77204533	AG	A	
ICC15	FAS	Missense	chr10	90762811	90762811	C	G	FAS:NM_152871:exon2:c.C56G:p.S19W
ICC5	FBXL19	Silent	chr16	30941489	30941489	G	A	FBXL19:NM_001099784:exon7:c.G945A:p.Q315Q
ICC4	FCGR3B	Missense	chr1	161596040	161596040	G	A	FCGR3B:NM_001271036:exon4:c.C421T:p.H141Y
ICC6	FCGR3B	Missense	chr1	161599779	161599779	G	C	FCGR3B:NM_001271036:exon3:c.C57G:p.S19R
ICC6	FCGR3B	Silent	chr1	161599773	161599773	A	G	FCGR3B:NM_001271036:exon3:c.T63C:p.L21L
ICC8	FCGR3B	Missense	chr1	161594399	161594399	G	A	FCGR3B:NM_001271036:exon5:c.C557T:p.S186F
ICC8	FCGR3B	Readthrough	chr1	161594307	161594307	A	G	FCGR3B:NM_001271036:exon5:c.T649C:p.X217R
ICC4	FCHSD1	Silent	chr5	141023863	141023863	A	T	FCHSD1:NM_033449:exon17:c.T1785A:p.R595R
ICC3	FKTN	Missense	chr9	108382330	108382330	G	A	FKTN:NM_001198963:exon10:c.G1160A:p.G387E
ICC15	FMNL1	Missense	chr17	43323305	43323305	G	C	FMNL1:NM_005892:exon24:c.G3055C:p.D1019H
ICC16	FN3KRP	Missense	chr17	80674699	80674699	G	A	FN3KRP:NM_024619:exon1:c.G68A:p.G23E
ICC10	FNDC3B	Silent	chr3	172048427	172048427	G	T	FNDC3B:NM_022763:exon13:c.G1476T:p.T492T
ICC8	FOSL2	Missense	chr2	28627143	28627143	T	A	FOSL2:NM_005253:exon2:c.T272A:p.V91D
ICC8	FPGS	Missense	chr9	130573240	130573240	A	T	FPGS:NM_001018078:exon14:c.A1154T:p.Y385F
ICC8	FRRS1	Missense	chr1	100174510	100174510	C	A	FRRS1:NM_001013660:exon17:c.G1825T:p.D609Y
ICC10	FUT5	Silent	chr19	5866816	5866816	G	A	FUT5:NM_002034:exon2:c.C921T:p.D307D
ICC11	GABRP	Silent	chr5	170215638	170215638	T	C	GABRP:NM_014211:exon2:c.T19C:p.L7L
ICC8	GALNT1	Missense	chr18	33282942	33282942	G	C	GALNT1:NM_020474:exon9:c.G1381C:p.G461R
ICC6	GATSL3	Missense	chr22	30682352	30682352	C	T	GATSL3:NM_001037666:exon6:c.G643A:p.A215T
ICC4	GCC2	Missense	chr2	109089299	109089299	C	T	GCC2:NM_181453:exon7:c.C2804T:p.S935L
ICC8	GLB1L2	Missense	chr11	134244595	134244595	A	G	GLB1L2:NM_138342:exon18:c.A1807G:p.S603G
ICC11	GLUD2	Missense	chrX	120182659	120182659	T	C	GLUD2:NM_012084:exon1:c.T1121C:p.V374A
ICC11	GPBP1	Missense	chr5	56527090	56527090	A	G	GPBP1:NM_001127236:exon4:c.A374G:p.N125S
ICC5	GPR126	Missense	chr6	142688869	142688869	A	C	GPR126:NM_001032395:exon3:c.A267C:p.E89D
ICC6	GRB7	Silent	chr17	37899446	37899446	G	A	GRB7:NM_001242442:exon5:c.G546A:p.E182E

ICC16	GRIN3B	Missense	chr19	1009534	1009534	T	C	GRIN3B:NM_138690:exon9:c.T3065C:p.L1022S
ICC8	GSTM2	Silent	chr1	110214175	110214175	A	T	GSTM2:NM_001142368:exon7:c.A537T:p.P179P
ICC3	GSTT2	Missense	chr22	24300580	24300580	C	T	GSTT2:NM_000854:exon4:c.G417A:p.M139I
ICC4	GTF2I	Missense	chr7	74146979	74146979	A	C	GTF2I:NM_001163636:exon14:c.A1214C:p.E405A
ICC6	GTPBP1	Missense	chr22	39126647	39126647	G	A	GTPBP1:NM_004286:exon12:c.G1948A:p.G650R
ICC8	GTPBP4	Missense	chr10	1042167	1042167	G	A	GTPBP4:NM_012341:exon4:c.G445A:p.E149K
ICC4	GYS1	Missense	chr19	49484850	49484850	G	C	GYS1:NM_001161587:exon7:c.C914G:p.P305R
ICC15	H2AFX	Nonsense	chr11	118965921	118965921	C	A	H2AFX:NM_002105:exon1:c.G184T:p.E62X
ICC8	H2AFZ	Silent	chr4	100870026	100870026	A	G	H2AFZ:NM_002106:exon4:c.T267C:p.L89L
ICC10	H6PD	Frameshift	chr1	9324326	9324333	GGCTCGAG	G	H6PD:NM_004285:exon5:c.1774_1781G
ICC15	HARS	Silent	chr5	140056437	140056437	A	T	HARS:NM_001258041:exon10:c.T936A:p.T312T
ICC8	HELZ	Missense	chr17	65190136	65190136	G	C	HELZ:NM_014877:exon9:c.C504G:p.F168L
ICC8	HERC2	Silent	chr15	28517382	28517382	G	C	HERC2:NM_004667:exon9:c.C1062G:p.P354P
ICC8	HGSNAT	Missense	chr8	43046689	43046689	G	T	HGSNAT:NM_152419:exon12:c.G1201T:p.G401C
ICC8	HLA-DQB1	Missense	chr6	32632757	32632757	C	T	HLA-DQB1:NM_002123:exon2:c.G197A:p.R66Q
ICC8	HN1	Missense	chr17	73150471	73150471	T	G	HN1:NM_001002032:exon1:c.G22C:p.E8Q
ICC6	HNRNPA1L2	Missense	chr13	53217547	53217547	T	G	HNRNPA1L2:NM_001011725:exon6:c.T920G:p.V307G
ICC8	HNRNPAB	Missense	chr5	177631843	177631843	C	T	HNRNPAB:NM_004499:exon2:c.C5T:p.S2L
ICC8	HPR	Silent	chr16	72110764	72110764	C	T	HPR:NM_020995:exon5:c.C831T:p.N277N
ICC11	IDH1	Missense	chr2	209113113	209113113	G	A	IDH1:NM_001282387:exon4:c.C394T:p.R132C
ICC6	IFT81	Frameshift	chr12	110566871	110566871	T	TT	IFT81:NM_001143779:exon4:c.365_365delinsTT
ICC14	IGF2BP2	Missense	chr3	185390409	185390409	A	G	IGF2BP2:NM_006548:exon10:c.T1120C:p.F374L
ICC3	IGLL5	Missense	chr22	23237570	23237570	A	G	IGLL5:NM_001256296:exon2:c.A116G:p.N39S
ICC3	IGLL5	Silent	chr22	23235982	23235982	C	G	IGLL5:NM_001178126:exon2:c.C309G:p.T103T
ICC8	ILDR1	Silent	chr3	121712495	121712495	C	A	ILDR1:NM_001199800:exon5:c.G834T:p.G278G
ICC3	IQSEC3	Missense	chr12	275027	275027	T	G	IQSEC3:NM_001170738:exon11:c.T2942G:p.V981G
ICC3	ITGA4	Missense	chr2	182347347	182347347	G	A	ITGA4:NM_000885:exon9:c.G1010A:p.G337E

ICC16	ITPR3	Silent	chr6	33655290	33655290	G	A	ITPR3:NM_002224:exon46:c.G6213A:p.L2071L
ICC4	ITSN2	Missense	chr2	24493592	24493592	A	G	ITSN2:NM_147152:exon20:c.T2303C:p.F768S
ICC8	JAK1	Missense	chr1	65307258	65307258	G	C	JAK1:NM_002227:exon18:c.C2430G:p.C810W
ICC4	JUP	Silent	chr17	39921215	39921215	T	C	JUP:NM_002230:exon6:c.A1014G:p.L338L
ICC11	KBTBD6	Frameshift	chr13	41704817	41704817	A	AA	KBTBD6:NM_152903:exon1:c.1831_1831delinsTT
ICC10	KCNMA1	Missense	chr10	79397298	79397298	T	C	KCNMA1:NM_001271521:exon1:c.A103G:p.S35G
ICC8	KIAA0556	Silent	chr16	27709782	27709782	C	G	KIAA0556:NM_015202:exon9:c.C1074G:p.L358L
ICC11	KIAA1217	Missense	chr10	24833237	24833237	A	G	KIAA1217:NM_019590:exon19:c.A5038G:p.I1680V
ICC11	KIAA2026	Frameshift	chr9	5944880	5944880	T	TT	KIAA2026:NM_001017969:exon5:c.2373_2373delinsAA
ICC8	KIDINS220	Missense	chr2	8943218	8943218	A	G	KIDINS220:NM_020738:exon8:c.T643C:p.Y215H
ICC8	KIF13A	Missense	chr6	17951634	17951634	T	C	KIF13A:NM_001243423:exon3:c.A173G:p.N58S
ICC8	KLHDC7A	Missense	chr1	18808310	18808310	G	A	KLHDC7A:NM_152375:exon1:c.G835A:p.E279K
ICC15	KMT2D	Missense	chr12	49422659	49422659	T	G	KMT2D:NM_003482:exon45:c.A14334C:p.K4778N
ICC5	KRAS	Missense	chr12	25398284	25398284	C	T	KRAS:NM_004985:exon2:c.G35A:p.G12D
ICC6	KRAS	Missense	chr12	25398281	25398281	C	T	KRAS:NM_004985:exon2:c.G38A:p.G13D
ICC15	KTI12	Missense	chr1	52499259	52499259	G	A	KTI12:NM_138417:exon1:c.C175T:p.R59C
ICC15	LAMA5	Silent	chr20	60899587	60899587	C	T	LAMA5:NM_005560:exon42:c.G5553A:p.R1851R
ICC16	LAMA5	Missense	chr20	60888413	60888413	A	C	LAMA5:NM_005560:exon64:c.T8762G:p.L2921R
ICC16	LAMB3	Missense	chr1	209804031	209804031	T	C	LAMB3:NM_001127641:exon9:c.A872G:p.E291G
ICC8	LAMTOR4	Splicing	chr7	99747121	99747121	G	C	LAMTOR4(NM_001008395:exon2:c.4-1G > C)
ICC4	LGALS9B	Missense	chr17	20353378	20353378	C	G	LGALS9B:NM_001042685:exon11:c.G976C:p.V326L
ICC6	LGALS9B	Missense	chr17	20353378	20353378	C	G	LGALS9B:NM_001042685:exon11:c.G976C:p.V326L
ICC11	LIMCH1	Missense	chr4	41646606	41646606	A	G	LIMCH1:NM_001112717:exon10:c.A1061G:p.N354S
ICC4	LOC158434	Missense	chr9	98871285	98871285	T	C	LOC158434:NM_001256408:exon3:c.A68G:p.Q23R
ICC4	LOC158434	Silent	chr9	98871251	98871251	T	C	LOC158434:NM_001256408:exon3:c.A102G:p.R34R
ICC8	LRBA	Missense	chr4	151520237	151520237	G	A	LRBA:NM_006726:exon38:c.C5968T:p.R1990W
ICC8	LRIF1	Missense	chr1	111494191	111494191	T	A	LRIF1:NM_018372:exon2:c.A1315T:p.N439Y

ICC5	LRRC37A2	Missense	chr17	44632693	44632693	A	C	LRRC37A2:NM_001006607:exon13:c.A4957C:p.M1653L
ICC15	LRRC40	Missense	chr1	70671147	70671147	G	A	LRRC40:NM_017768:exon1:c.C77T:p.S26L
ICC16	LRRCC1	Missense	chr8	86038951	86038951	A	G	LRRCC1:NM_033402:exon9:c.A1300G:p.R434G
ICC10	LRTOMT	Silent	chr11	71806035	71806035	C	T	LRTOMT:NM_001145307:exon5:c.C330T:p.H110H
ICC8	LTB4R2	Missense	chr14	24780405	24780405	G	A	LTB4R2:NM_019839:exon2:c.G535A:p.A179T
ICC15	LZTS2	Silent	chr10	102766820	102766820	G	T	LZTS2:NM_032429:exon5:c.G1905T:p.L635L
ICC15	MAMDC4	Frameshift	chr9	139751241	139751241	G	GG GAG	MAMDC4:NM_206920:exon15:c.1800_1800delinsGGGAG
ICC4	MAML2	Silent	chr11	95825410	95825410	C	T	MAML2:NM_032427:exon2:c.G1785A:p.Q595Q
ICC11	MAP3K11	Silent	chr11	65365858	65365858	T	A	MAP3K11:NM_002419:exon10:c.A2448T:p.P816P
ICC8	MAP4K2	Missense	chr11	64564817	64564817	G	C	MAP4K2:NM_004579:exon18:c.C1206G:p.I402M
ICC6	MAP4K5	Nonframeshift	chr14	50906732	50906732	T	TTCT	MAP4K5:NM_198794:exon23:c.1704_1704delinsAGAA
ICC15	MAPKBP1	Missense	chr15	42109135	42109135	G	A	MAPKBP1:NM_001128608:exon15:c.G1631A:p.R544Q
ICC8	MAST4	Silent	chr5	66461133	66461133	G	A	MAST4:NM_015183:exon28:c.G5559A:p.T1853T
ICC8	MBD5	Missense	chr2	149247930	149247930	A	G	MBD5:NM_018328:exon12:c.A4030G:p.S1344G
ICC8	MED15	Missense	chr22	20918961	20918961	C	A	MED15:NM_015889:exon6:c.C676A:p.Q226K
ICC14	MEF2BNB	Splicing	chr19	19288271	19288271	T	C	MEF2BNB(NM_001145784:exon7:c.403-2A > G)
ICC15	MEGF8	Nonsense	chr19	42867324	42867324	T	A	MEGF8:NM_001410:exon34:c.T5982A:p.C1994X
ICC4	MFAP3	Missense	chr5	153433260	153433260	A	T	MFAP3:NM_001135037:exon2:c.A638T:p.N213I
ICC11	MIDN	Missense	chr19	1254276	1254276	C	G	MIDN:NM_177401:exon5:c.C495G:p.H165Q
ICC16	MINOS1	Missense	chr1	19950062	19950062	T	A	MINOS1:NM_001204082:exon2:c.T159A:p.H53Q
ICC8	MMP14	Missense	chr14	23315000	23315000	G	A	MMP14:NM_004995:exon10:c.G1501A:p.A501T
ICC8	MPP3	Missense	chr17	41891699	41891699	C	T	MPP3:NM_001932:exon15:c.G1040A:p.C347Y
ICC10	MRAS	Missense	chr3	138121025	138121025	G	A	MRAS:NM_001252093:exon5:c.G313A:p.E105K
ICC8	MRC1	Missense	chr10	17869577	17869577	C	T	MRC1:NM_002438:exon3:c.C500T:p.T167I
ICC10	MT1A	Missense	chr16	56673821	56673821	A	G	MT1A:NM_005946:exon3:c.A145G:p.I49V
ICC10	MT1A	Silent	chr16	56673838	56673838	A	G	MT1A:NM_005946:exon3:c.A162G:p.S54S
ICC5	MTF2	Missense	chr1	93599721	93599721	A	G	MTF2:NM_001164391:exon15:c.A1087G:p.T363A

ICC5	MTMR10	Silent	chr15	31260142	31260142	C	T	MTMR10:NM_017762:exon6:c.G546A:p.G182G
ICC6	MUC20	Silent	chr3	195452632	195452632	C	T	MUC20:NM_001282506:exon2:c.C1158T:p.S386S
ICC10	MVD	Missense	chr16	88722569	88722569	C	T	MVD:NM_002461:exon5:c.G547A:p.A183T
ICC4	MYO5A	Nonframeshift	chr15	52643454	52643454	G	GTCATCCTTG	MYO5A:NM_001142495:exon28:c.3846_3846delinsCAAGGATGAC
ICC5	N4BP2L2	Frameshift	chr13	33017582	33017582	A	AA	N4BP2L2:NM_033111:exon7:c.1092_1092delinsTT
ICC8	NABP2	Silent	chr12	56619996	56619996	C	T	NABP2:NM_024068:exon5:c.C324T:p.F108F
ICC3	NBPF10	Silent	chr1	145296510	145296510	C	G	NBPF10:NM_001039703:exon3:c.C432G:p.L144L
ICC4	NBPF10	Missense	chr1	145298272	145298272	A	C	NBPF10:NM_001039703:exon5:c.A684C:p.K228N
ICC8	NBPF10	Missense	chr1	145299906	145299906	T	G	NBPF10:NM_001039703:exon6:c.T955G:p.S319A
ICC8	NBPF10	Missense	chr1	145301750	145301750	T	C	NBPF10:NM_001039703:exon7:c.T1019C:p.F340S
ICC8	NBPF10	Silent	chr1	145301739	145301739	C	T	NBPF10:NM_001039703:exon7:c.C1008T:p.D336D
ICC3	NBPF11	Silent	chr1	146035094	146035094	T	C	NBPF11:NM_183372:exon23:c.A2688G:p.R896R
ICC5	NBPF12	Missense	chr1	146399596	146399596	G	C	NBPF12:NM_001278141:exon10:c.G535C:p.D179H
ICC4	NBPF16	Missense	chr1	148592119	148592119	G	A	NBPF16:NM_001102663:exon13:c.G1475A:p.G492D
ICC10	NBR1	Missense	chr17	41352493	41352493	C	T	NBR1:NM_031862:exon17:c.C2336T:p.A779V
ICC16	NDRG1	Missense	chr8	134251224	134251224	C	T	NDRG1:NM_001258433:exon15:c.G839A:p.R280H
ICC6	NFKBIZ	Missense	chr3	101570929	101570929	T	C	NFKBIZ:NM_031419:exon2:c.T290C:p.V97A
ICC8	NONO	Missense	chrX	70516476	70516476	G	A	NONO:NM_001145408:exon7:c.G712A:p.E238K
ICC16	NOP56	Missense	chr20	2638684	2638684	A	C	NOP56:NM_006392:exon12:c.A1529C:p.K510T
ICC10	NOTCH2	Missense	chr1	120572572	120572572	C	T	NOTCH2:NM_024408:exon2:c.G112A:p.E38K
ICC10	NOTCH2	Missense	chr1	120572547	120572547	T	C	NOTCH2:NM_024408:exon2:c.A137G:p.N46S
ICC4	NOTCH2	Missense	chr1	120539668	120539668	T	A	NOTCH2:NM_024408:exon4:c.A703T:p.T235S
ICC6	NPAT	Silent	chr11	108032702	108032702	C	T	NPAT:NM_002519:exon17:c.G3111A:p.G1037G
ICC8	NPEPPS	Missense	chr17	45668095	45668095	C	A	NPEPPS:NM_006310:exon10:c.C1108A:p.H370N
ICC4	NPIPA2	Missense	chr16	14819896	14819896	C	T	NPIPA2:NM_001277324:exon8:c.C799T:p.P267S

ICC10	NPIPB5	Silent	chr16	22546842	22546842	T	C	NPIPB5:NM_001135865:exon7:c.T2538C:p.P846P
ICC3	NRP2	Nonsense	chr2	206610604	206610604	T	A	NRP2:NM_201267:exon10:c.T1776A:p.C592X
ICC8	NSD1	Missense	chr5	176707753	176707753	C	G	NSD1:NM_022455:exon18:c.C5810G:p.S1937C
ICC15	NUP210	Missense	chr3	13372064	13372064	C	G	NUP210:NM_024923:exon30:c.G4006C:p.E1336Q
ICC5	OBSCN	Missense	chr1	228528856	228528856	C	T	OBSCN:NM_001271223:exon84:c.C20629T:p.R6877C
ICC8	OBSL1	Silent	chr2	220431760	220431760	G	T	OBSL1:NM_001173408:exon5:c.C1926A:p.I642I
ICC8	OCIAD2	Silent	chr4	48887540	48887540	T	A	OCIAD2:NM_001014446:exon7:c.A426T:p.S142S
ICC14	OGFR	Missense	chr20	61444840	61444840	C	A	OGFR:NM_007346:exon7:c.C1873A:p.R625S
ICC8	OS9	Missense	chr12	58114619	58114619	A	C	OS9:NM_001017956:exon14:c.A1766C:p.E589A
ICC6	OXCT1	Silent	chr5	41739525	41739525	A	G	OXCT1:NM_000436:exon16:c.T1488C:p.D496D
ICC8	P4HB	Missense	chr17	79817234	79817234	G	C	P4HB:NM_000918:exon2:c.C175G:p.L59V
ICC8	PAF1	Missense	chr19	39879943	39879943	G	A	PAF1:NM_001256826:exon5:c.C410T:p.S137F
ICC15	PARP9	Missense	chr3	122274786	122274786	T	C	PARP9:NM_001146102:exon4:c.A337G:p.M113V
ICC8	PCNT	Missense	chr21	47836722	47836722	C	G	PCNT:NM_006031:exon30:c.C6890G:p.A2297G
ICC15	PDCD11	Silent	chr10	105203063	105203063	C	T	PDCD11:NM_014976:exon33:c.C5097T:p.D1699D
ICC4	PDCD7	Missense	chr15	65426067	65426067	T	C	PDCD7:NM_005707:exon1:c.A53G:p.Q18R
ICC8	PDCD7	Silent	chr15	65426084	65426084	T	C	PDCD7:NM_005707:exon1:c.A36G:p.P12P
ICC15	PDE4DIP	Silent	chr1	144921924	144921924	G	A	PDE4DIP:NM_014644:exon9:c.C1105T:p.L369L
ICC16	PDE4DIP	Missense	chr1	144917841	144917841	T	C	PDE4DIP:NM_014644:exon11:c.A1445G:p.H482R
ICC16	PDE4DIP	Nonsense	chr1	144915561	144915561	G	A	PDE4DIP:NM_014644:exon14:c.C1864T:p.R622X
ICC16	PDE8A	Silent	chr15	85656666	85656666	G	T	PDE8A:NM_001243137:exon13:c.G957T:p.A319A
ICC8	PDPR	Missense	chr16	70154480	70154480	A	G	PDPR:NM_017990:exon3:c.A85G:p.T29A
ICC8	PELI3	Missense	chr11	66238754	66238754	G	A	PELI3:NM_001098510:exon3:c.G194A:p.R65Q
ICC14	PGBD1	Frameshift	chr6	28268666	28268666	A	AA	PGBD1:NM_032507:exon7:c.1035_1035delinsAA
ICC16	PIK3R4	Missense	chr3	130435311	130435311	C	G	PIK3R4:NM_014602:exon9:c.G2260C:p.G754R
ICC8	PKHD1	Missense	chr6	51890914	51890914	C	T	PKHD1:NM_138694:exon32:c.G3694A:p.V1232M
ICC15	PKN2	Missense	chr1	89236082	89236082	G	C	PKN2:NM_006256:exon4:c.G552C:p.K184N

ICC8	PKNOX1	Silent	chr21	44450196	44450196	T	C	PKNOX1:NM_004571:exon11:c.T1296C:p.S432S
ICC3	PLEKHM1	Missense	chr17	43552520	43552520	C	T	PLEKHM1:NM_014798:exon4:c.G869A:p.C290Y
ICC3	PLEKHM1	Missense	chr17	43552478	43552478	C	T	PLEKHM1:NM_014798:exon4:c.G911A:p.R304Q
ICC6	PLXNB2	Nonframeshift	chr22	50719580	50719583	ATCT	A	PLXNB2:NM_012401:exon23:c.3698_3701T
ICC8	PNN	Missense	chr14	39647083	39647083	A	G	PNN:NM_002687:exon6:c.A461G:p.Q154R
ICC8	POMGNT1	Missense	chr1	46654420	46654420	G	C	POMGNT1:NM_001243766:exon23:c.C2218G:p.L740V
ICC8	PORCN	Missense	chrX	48370779	48370779	G	A	PORCN:NM_001282167:exon5:c.G226A:p.G76S
ICC16	POT1	Missense	chr7	124493121	124493121	C	A	POT1:NM_015450:exon10:c.G774T:p.M258I
ICC8	PPARD	Silent	chr6	35392426	35392426	C	T	PPARD:NM_001171819:exon6:c.C831T:p.F277F
ICC8	PPIP5K2	Missense	chr5	102513396	102513396	C	G	PPIP5K2:NM_015216:exon21:c.C2558G:p.A853G
ICC3	PPP1R13L	Silent	chr19	45895561	45895561	C	T	PPP1R13L:NM_001142502:exon8:c.G1392A:p.P464P
ICC10	PPP2R1A	Nonsense	chr19	52729019	52729019	C	T	PPP2R1A:NM_014225:exon14:c.C1711T:p.Q571X
ICC8	PPP2R1A	Silent	chr19	52723099	52723099	C	T	PPP2R1A:NM_014225:exon10:c.C1284T:p.P428P
ICC11	PRDM16	Missense	chr1	3329336	3329336	T	C	PRDM16:NM_022114:exon9:c.T2575C:p.S859P
ICC10	PRKAG2	Missense	chr7	151267290	151267290	A	C	PRKAG2:NM_024429:exon6:c.T350G:p.F117C
ICC14	PRR12	Missense	chr19	50103177	50103177	A	T	PRR12:NM_020719:exon5:c.A4327T:p.N1443Y
ICC15	PRRT2	Missense	chr16	29824488	29824488	T	C	PRRT2:NM_001256443:exon2:c.T113C:p.L38P
ICC5	PRRT2	Missense	chr16	29824488	29824488	T	C	PRRT2:NM_001256443:exon2:c.T113C:p.L38P
ICC4	PRSS3	Missense	chr9	33797861	33797861	A	G	PRSS3:NM_001197098:exon3:c.A214G:p.K72E
ICC10	PSCA	Missense	chr8	143762807	143762807	G	A	PSCA:NM_005672:exon2:c.G88A:p.E30K
ICC10	PSCA	Silent	chr8	143763547	143763547	C	A	PSCA:NM_005672:exon3:c.C342A:p.L114L
ICC10	PSCA	Silent	chr8	143763490	143763490	T	C	PSCA:NM_005672:exon3:c.T285C:p.A95A
ICC8	PSMB10	Missense	chr16	67970627	67970627	C	A	PSMB10:NM_002801:exon1:c.G26T:p.R9L
ICC16	PSME2	Splicing	chr14	24614484	24614484	T	C	PSME2(NM_002818:exon6:c.232-2A > G)
ICC6	PSMG2	Silent	chr18	12706644	12706644	T	C	PSMG2:NM_020232:exon2:c.T153C:p.C51C
ICC5	PSPH	Missense	chr7	56088779	56088779	C	T	PSPH:NM_004577:exon4:c.G127A:p.A43T
ICC11	PSRC1	Missense	chr1	109824620	109824620	G	A	PSRC1:NM_032636:exon4:c.C140T:p.S47F
ICC16	PTPN14	Missense	chr1	214537983	214537983	T	C	PTPN14:NM_005401:exon18:c.A3307G:p.T1103A

ICC10	PTPN3	Nonsense	chr9	112200427	112200427	A	T	PTPN3:NM_001145369:exon3:c.T161A:p.L54X
ICC8	PTPRF	Missense	chr1	44084343	44084343	T	C	PTPRF:NM_002840:exon26:c.T4414C:p.C1472R
ICC10	PTPRG	Frameshift	chr3	62204534	62204534	A	AA	PTPRG:NM_002841:exon13:c.2165_2165delinsAA
ICC4	PVRIG	Missense	chr7	99817761	99817761	T	C	PVRIG:NM_024070:exon3:c.T143C:p.V48A
ICC10	QRICH1	Missense	chr3	49095196	49095196	G	A	QRICH1:NM_198880:exon3:c.C437T:p.S146L
ICC8	RAB11A	Silent	chr15	66172028	66172028	C	T	RAB11A:NM_004663:exon4:c.C450T:p.F150F
ICC8	RAI14	Missense	chr5	34829888	34829888	C	G	RAI14:NM_001145523:exon16:c.C2827G:p.L943V
ICC16	RALGDS	Missense	chr9	135985719	135985719	G	A	RALGDS:NM_001042368:exon3:c.C287T:p.T96I
ICC3	RASSF8	Missense	chr12	26217980	26217980	T	C	RASSF8:NM_001164746:exon3:c.T653C:p.V218A
ICC8	RBMX	Missense	chrX	135958783	135958783	C	T	RBMX:NM_002139:exon5:c.G420A:p.M140I
ICC4	RBMXL1	Missense	chr1	89448623	89448623	A	G	RBMXL1:NM_019610:exon2:c.T887C:p.L296P
ICC6	RBMXL1	Missense	chr1	89449390	89449390	T	C	RBMXL1:NM_019610:exon2:c.A120G:p.I40M
ICC4	RERE	Missense	chr1	8674712	8674712	T	C	RERE:NM_012102:exon5:c.A430G:p.T144A
ICC16	RFPL4A	Silent	chr19	56274424	56274424	C	A	RFPL4A:NM_001145014:exon3:c.C747A:p.P249P
ICC16	RFWD3	Missense	chr16	74666479	74666479	T	C	RFWD3:NM_018124:exon9:c.A1519G:p.S507G
ICC16	RHBDF2	Nonsense	chr17	74469139	74469139	C	A	RHBDF2:NM_001005498:exon16:c.G1858T:p.E620X
ICC11	RHPN1	Missense	chr8	144463817	144463817	G	A	RHPN1:NM_052924:exon13:c.G1564A:p.G522S
ICC4	RHPN1	Missense	chr8	144464801	144464801	A	G	RHPN1:NM_052924:exon15:c.A1993G:p.K665E
ICC4	RNF149	Missense	chr2	101898414	101898414	C	T	RNF149:NM_173647:exon6:c.G1066A:p.D356N
ICC14	RNMT	Nonsense	chr18	13737100	13737100	G	A	RNMT:NM_003799:exon5:c.G645A:p.W215X
ICC8	RPL10	Missense	chrX	153629234	153629234	A	T	RPL10:NM_001256577:exon6:c.A521T:p.N174I
ICC16	RTCA	Missense	chr1	100757031	100757031	G	A	RTCA:NM_001130841:exon12:c.G1111A:p.G371R
ICC4	RTFDC1	Missense	chr20	55092192	55092192	G	A	RTFDC1:NM_016407:exon8:c.G677A:p.G226E
ICC8	RTTN	Silent	chr18	67857849	67857849	G	A	RTTN:NM_173630:exon9:c.C1114T:p.L372L
ICC15	RUFY1	Missense	chr5	178977596	178977596	C	T	RUFY1:NM_025158:exon1:c.C26T:p.A9V
ICC8	S100P	Nonsense	chr4	6698749	6698749	G	T	S100P:NM_005980:exon2:c.G268T:p.E90X
ICC8	SBF2	Missense	chr11	9989911	9989911	A	G	SBF2:NM_030962:exon14:c.T1577C:p.V526A

ICC3	SEC11C	Missense	chr18	56819785	56819785	C	G	SEC11C:NM_033280:exon3:c.C215G:p.A72G
ICC15	SEC14L6	Missense	chr22	30921695	30921695	T	C	SEC14L6:NM_001193336:exon10:c.A814G:p.K272E
ICC16	SEC23IP	Missense	chr10	121692528	121692528	G	A	SEC23IP:NM_007190:exon17:c.G2770A:p.V924I
ICC15	SENP7	Missense	chr3	101059033	101059033	G	C	SENP7:NM_001282803:exon13:c.C1771G:p.P591A
ICC8	SETD2	Missense	chr3	47165300	47165300	C	G	SETD2:NM_014159:exon3:c.G826C:p.D276H
ICC8	SF3B1	Missense	chr2	198283248	198283248	G	C	SF3B1:NM_012433:exon5:c.C480G:p.H160Q
ICC16	SH3BP2	Missense	chr4	2835556	2835556	A	G	SH3BP2:NM_001145856:exon13:c.A1852G:p.R618G
ICC10	SH3BP4	Nonsense	chr2	235951091	235951091	C	T	SH3BP4:NM_014521:exon4:c.C1678T:p.R560X
ICC10	SH3BP5	Missense	chr3	15297738	15297738	C	T	SH3BP5:NM_004844:exon9:c.G1223A:p.S408N
ICC15	SH3YL1	Missense	chr2	242800	242800	T	C	SH3YL1:NM_001282682:exon6:c.A1G:p.M1V
ICC8	SHROOM3	Silent	chr4	77661573	77661573	G	A	SHROOM3:NM_020859:exon5:c.G2247A:p.S749S
ICC8	SHROOM4	Silent	chrX	50345717	50345717	C	T	SHROOM4:NM_020717:exon7:c.G3858A:p.L1286L
ICC8	SLC30A1	Nonsense	chr1	211751857	211751857	G	T	SLC30A1:NM_021194:exon1:c.C98A:p.S33X
ICC8	SLC36A4	Silent	chr11	92917650	92917650	A	G	SLC36A4:NM_152313:exon3:c.T216C:p.N72N
ICC10	SLC38A10	Missense	chr17	79226927	79226927	G	A	SLC38A10:NM_138570:exon12:c.G1402T:p.A468S
ICC15	SLC6A6	Missense	chr3	14485200	14485200	C	G	SLC6A6:NM_001134367:exon3:c.C58G:p.P20A
ICC6	SLC9A6	Missense	chrX	135092644	135092644	T	C	SLC9A6:NM_006359:exon7:c.T847C:p.F283L
ICC8	SMAD4	Missense	chr18	48575208	48575208	A	T	SMAD4:NM_005359:exon3:c.A402T:p.E134D
ICC3	SMARCA4	Missense	chr19	11141502	11141502	G	T	SMARCA4:NM_001128845:exon24:c.G3479T:p.G1160V
ICC8	SMARCAL1	Missense	chr2	217303154	217303154	C	G	SMARCAL1:NM_014140:exon10:c.C1656G:p.H552Q
ICC6	SMARCC1	Silent	chr3	47719729	47719729	A	T	SMARCC1:NM_003074:exon16:c.T1530A:p.A510A
ICC3	SMC4	Silent	chr3	160141344	160141344	A	T	SMC4:NM_001002800:exon14:c.A2151T:p.V717V
ICC14	SOS1	Silent	chr2	39239440	39239440	C	T	SOS1:NM_005633:exon14:c.G2217A:p.R739R
ICC15	SPATA2	Missense	chr20	48524735	48524735	T	C	SPATA2:NM_001135773:exon2:c.A293G:p.N98S
ICC6	SPEN	Missense	chr1	16257668	16257668	G	A	SPEN:NM_015001:exon11:c.G4933A:p.V1645I

ICC15	SPG11	Missense	chr15	44888478	44888478	C	A	SPG11:NM_001160227:exon25:c.G4237T:p.V1413L
ICC5	SPIRE1	Silent	chr18	12479725	12479725	C	A	SPIRE1:NM_001128627:exon9:c.G975T:p.L325L
ICC15	SPRED1	Missense	chr15	38643399	38643399	C	T	SPRED1:NM_152594:exon7:c.C869T:p.S290L
ICC3	SPRED1	Missense	chr15	38614578	38614578	G	A	SPRED1:NM_152594:exon3:c.G344A:p.G115D
ICC15	SRCAP	Missense	chr16	30720856	30720856	C	T	SRCAP:NM_006662:exon7:c.C656T:p.S219F
ICC8	SRFBP1	Missense	chr5	121362765	121362765	G	C	SRFBP1:NM_152546:exon8:c.G1234C:p.E412Q
ICC15	STAP2	Missense	chr19	4324641	4324641	C	T	STAP2:NM_017720:exon12:c.G1096A:p.A366T
ICC11	STARD9	Frameshift	chr15	42983833	42983834	CA	C	STARD9:NM_020759:exon23:c.10057_10058C
ICC8	STAT5A	Silent	chr17	40453362	40453362	C	T	STAT5A:NM_003152:exon10:c.C1059T:p.R353R
ICC8	STXBP4	Missense	chr17	53084911	53084911	A	G	STXBP4:NM_178509:exon8:c.A619G:p.I207V
ICC15	STYX	Missense	chr14	53235599	53235599	G	C	STYX:NM_001130701:exon11:c.G546C:p.M182I
ICC8	SUMF1	Missense	chr3	4508853	4508853	G	A	SUMF1:NM_001164675:exon1:c.C77T:p.S26L
ICC11	SYNE2	Missense	chr14	64610566	64610566	G	C	SYNE2:NM_015180:exon83:c.G15383C:p.C5128S
ICC11	TAF2	Missense	chr8	120774835	120774835	T	C	TAF2:NM_003184:exon19:c.A2378G:p.Y793C
ICC3	TBP	Silent	chr6	170871013	170871013	A	G	TBP:NM_001172085:exon2:c.A129G:p.Q43Q
ICC8	TCF7L2	Silent	chr10	114710992	114710992	C	T	TCF7L2:NM_001146284:exon2:c.C216T:p.S72S
ICC15	TGOLN2	Silent	chr2	85552107	85552107	G	A	TGOLN2:NM_001206844:exon4:c.C1065T:p.V355V
ICC6	TICAM1	Missense	chr19	4817266	4817266	T	C	TICAM1:NM_182919:exon2:c.A1124G:p.H375R
ICC16	TIMM23	Missense	chr10	51620361	51620361	G	C	TIMM23:NM_006327:exon2:c.C128G:p.S43C
ICC11	TMBIM1	Missense	chr2	219143263	219143263	T	C	TMBIM1:NM_022152:exon6:c.A448G:p.I150V
ICC8	TMC4	Silent	chr19	54672357	54672357	C	T	TMC4:NM_001145303:exon4:c.G510A:p.L170L
ICC4	TMC6	Silent	chr17	76113978	76113978	G	A	TMC6:NM_007267:exon16:c.C1926T:p.P642P
ICC6	TMCC3	Missense	chr12	94972226	94972226	C	T	TMCC3:NM_020698:exon3:c.G1075A:p.A359T
ICC14	TMEM131	Frameshift	chr2	98422026	98422026	T	TT	TMEM131:NM_015348:exon20:c.2196_2196delinsAA
ICC11	TMEM159	Missense	chr16	21181833	21181833	T	G	TMEM159:NM_020422:exon3:c.T172G:p.F58V

ICC16	TMEM2	Silent	chr9	74315641	74315641	G	T	TMEM2:NM_001135820:exon18:c.C3105A:p.I1035I
ICC4	TMEM230	Missense	chr20	5092172	5092172	T	C	TMEM230:NM_001009923:exon2:c.A148G:p.S50G
ICC8	TMPO	Missense	chr12	98927902	98927902	G	C	TMPO:NM_003276:exon4:c.G1867C:p.D623H
ICC16	TNFRSF21	Silent	chr6	47254023	47254023	A	G	TNFRSF21:NM_014452:exon2:c.T405C:p.P135P
ICC8	TOB2	Missense	chr22	41833096	41833096	T	A	TOB2:NM_016272:exon2:c.A254T:p.N85I
ICC8	TOE1	Missense	chr1	45806850	45806850	T	C	TOE1:NM_025077:exon2:c.T158C:p.L53P
ICC10	TP53	Missense	chr17	7578440	7578440	T	C	TP53:NM_001276698:exon1:c.A13G:p.K5E
ICC16	TP53	Nonsense	chr17	7578263	7578263	G	A	TP53:NM_001276698:exon2:c.C109T:p.R37X
ICC10	TRIM16L	Silent	chr17	18638653	18638653	G	A	TRIM16L:NM_001037330:exon5:c.G927A:p.K309K
ICC16	TRIM23	Missense	chr5	64906836	64906836	C	T	TRIM23:NM_033228:exon5:c.G680A:p.R227Q
ICC8	TRPS1	Missense	chr8	116616184	116616184	T	C	TRPS1:NM_014112:exon4:c.A2012G:p.Q671R
ICC16	TSC22D2	Missense	chr3	150128962	150128962	A	G	TSC22D2:NM_014779:exon1:c.A1825G:p.I609V
ICC15	TTC26	Silent	chr7	138831902	138831902	G	A	TTC26:NM_001144920:exon6:c.G411A:p.E137E
ICC8	TUBG2	Silent	chr17	40818353	40818353	C	T	TUBG2:NM_016437:exon10:c.C1009T:p.L337L
ICC15	TUBGCP3	Missense	chr13	113140317	113140317	G	C	TUBGCP3:NM_001286277:exon22:c.C2684G:p.S895C
ICC3	TYK2	Missense	chr19	10461630	10461630	C	G	TYK2:NM_003331:exon25:c.G3444C:p.M1148I
ICC11	UBC	Silent	chr12	125398267	125398267	A	C	UBC:NM_021009:exon2:c.T51G:p.V17V
ICC8	UBC	Silent	chr12	125398267	125398267	A	C	UBC:NM_021009:exon2:c.T51G:p.V17V
ICC8	UBTD2	Readthrough	chr5	171638835	171638835	C	A	UBTD2:NM_152277:exon3:c.G704T:p.X235L
ICC16	UBXN1	Missense	chr11	62445991	62445991	A	C	UBXN1:NM_001286077:exon3:c.T196G:p.S66A
ICC8	UCHL3	Missense	chr13	76140934	76140934	G	T	UCHL3:NM_006002:exon4:c.G287T:p.G96V
ICC16	UGT2B11	Silent	chr4	70071232	70071232	C	T	UGT2B11:NM_001073:exon4:c.G1056A:p.R352R
ICC16	UGT2B11	Silent	chr4	70070252	70070252	G	A	UGT2B11:NM_001073:exon5:c.C1206T:p.N402N
ICC8	ULK1	Missense	chr12	132394835	132394835	C	G	ULK1:NM_003565:exon11:c.C845G:p.P282R
ICC16	URGCP	Missense	chr7	43916423	43916423	C	T	URGCP:NM_001077664:exon6:c.G2510A:p.G837D
ICC15	VCP	Silent	chr9	35065278	35065278	G	T	VCP:NM_007126:exon5:c.C546A:p.I182I
ICC10	VGLL4	Missense	chr3	11600049	11600049	G	T	VGLL4:NM_001284390:exon7:c.C869A:p.S290Y
ICC10	VSIG10L	Silent	chr19	51840598	51840598	G	A	VSIG10L:NM_001163922:exon7:c.C2199T:p.A733A

ICC16	WASH1	Missense	chr9	16730	16730	C	T	WASH1:NM_182905:exon8:c.G1030A:p.A344T
ICC3	WASH1	Missense	chr9	16730	16730	C	T	WASH1:NM_182905:exon8:c.G1030A:p.A344T
ICC5	WASH1	Missense	chr9	16730	16730	C	T	WASH1:NM_182905:exon8:c.G1030A:p.A344T
ICC14	WDR11	Silent	chr10	122622314	122622314	G	A	WDR11:NM_018117:exon5:c.G594A:p.G198G
ICC3	WDR52	Frameshift	chr3	113025054	113025054	T	TT	WDR52:NM_001164496:exon30:c.4752_4752delinsAA
ICC8	WDR7	Missense	chr18	54349982	54349982	G	A	WDR7:NM_052834:exon5:c.G418A:p.V140I
ICC16	XPO1	Missense	chr2	61749770	61749770	T	C	XPO1:NM_003400:exon4:c.A277G:p.I93V
ICC8	XRCC6	Silent	chr22	42032665	42032665	G	A	XRCC6:NM_001469:exon5:c.G480A:p.K160K
ICC8	YARS2	Silent	chr12	32908221	32908221	C	T	YARS2:NM_001040436:exon1:c.G588A:p.G196G
ICC15	ZBTB7A	Missense	chr19	4054222	4054222	T	G	ZBTB7A:NM_015898:exon2:c.A1009C:p.S337R
ICC8	ZC3H7A	Missense	chr16	11846644	11846644	C	G	ZC3H7A:NM_014153:exon22:c.G2607C:p.E869D
ICC5	ZC3HAV1L	Missense	chr7	138720483	138720483	T	C	ZC3HAV1L:NM_080660:exon1:c.A281G:p.Y94C
ICC15	ZNF132	Silent	chr19	58945017	58945017	A	G	ZNF132:NM_003433:exon3:c.T1794C:p.P598P
ICC8	ZNF235	Missense	chr19	44803803	44803803	T	C	ZNF235:NM_004234:exon3:c.A98G:p.Y33C
ICC8	ZNF250	Missense	chr8	146107022	146107022	C	A	ZNF250:NM_021061:exon6:c.G1561T:p.V521L
ICC15	ZNF334	Silent	chr20	45130634	45130634	G	A	ZNF334:NM_001270497:exon6:c.C1341T:p.L447L
ICC11	ZNF354B	Nonsense	chr5	178309766	178309766	G	T	ZNF354B:NM_058230:exon5:c.G313T:p.E105X
ICC8	ZNF37A	Silent	chr10	38407606	38407606	A	G	ZNF37A:NM_001007094:exon8:c.A1527G:p.K509K
ICC11	ZNF429	Missense	chr19	21720411	21720411	T	A	ZNF429:NM_001001415:exon4:c.T1556A:p.I519N
ICC8	ZNF445	Silent	chr3	44489678	44489678	A	G	ZNF445:NM_181489:exon8:c.T1485C:p.S495S
ICC16	ZNF521	Missense	chr18	22671992	22671992	C	G	ZNF521:NM_015461:exon6:c.G3712C:p.A1238P
ICC4	ZNF527	Silent	chr19	37880682	37880682	T	C	ZNF527:NM_032453:exon5:c.T1731C:p.A577A
ICC16	ZNF559	Missense	chr19	9449888	9449888	A	G	ZNF559:NM_001202406:exon4:c.A245G:p.D82G
ICC11	ZNF587B	Missense	chr19	58355651	58355651	A	G	ZNF587B:NM_001204818:exon4:c.A1150G:p.K384E
ICC10	ZNF616	Missense	chr19	52619071	52619071	T	G	ZNF616:NM_178523:exon4:c.A1346C:p.H449P
ICC15	ZNF629	Missense	chr16	30795051	30795051	G	C	ZNF629:NM_001080417:exon3:c.C598G:p.H200D

ICC10	ZNF669	Missense	chr1	247263717	247263717	T	C	ZNF669:NM_001142572:exon4:c.A1096G:p.N366D
ICC8	ZNF669	Missense	chr1	247263717	247263717	T	C	ZNF669:NM_001142572:exon4:c.A1096G:p.N366D
ICC10	ZNF681	Silent	chr19	23927185	23927185	A	T	ZNF681:NM_138286:exon4:c.T1167A:p.T389T
ICC15	ZNF684	Silent	chr1	41012544	41012544	G	A	ZNF684:NM_152373:exon5:c.G549A:p.R183R
ICC16	ZNF700	Missense	chr19	12059912	12059912	G	C	ZNF700:NM_144566:exon4:c.G1073C:p.G358A
ICC4	ZNF721	Missense	chr4	437300	437300	T	A	ZNF721:NM_133474:exon3:c.A956T:p.N319I
ICC8	ZNF776	Missense	chr19	58265844	58265844	A	G	ZNF776:NM_173632:exon3:c.A1346G:p.Q449R
ICC15	ZNF799	Readthrough	chr19	12501281	12501281	T	A	ZNF799:NM_001080821:exon4:c.A1931T:p.X644L
ICC15	ZNF799	Silent	chr19	12501451	12501451	C	T	ZNF799:NM_001080821:exon4:c.G1761A:p.P587P
ICC11	ZNF814	Silent	chr19	58385762	58385762	C	G	ZNF814:NM_001144989:exon3:c.G996C:p.S332S
ICC15	ZNF836	Silent	chr19	52658647	52658647	C	T	ZNF836:NM_001102657:exon5:c.G2289A:p.S763S
ICC10	ZNF91	Missense	chr19	23542297	23542297	T	C	ZNF91:NM_003430:exon4:c.A3484G:p.I1162V
ICC10	ZSCAN30	Missense	chr18	32833830	32833830	A	G	ZSCAN30:NM_001112734:exon4:c.T1069C:p.Y357H
ICC10	ZSCAN30	Silent	chr18	32833948	32833948	A	G	ZSCAN30:NM_001112734:exon4:c.T951C:p.I317I
ICC10	ZSCAN30	Silent	chr18	32833915	32833915	A	G	ZSCAN30:NM_001112734:exon4:c.T984C:p.P328P
ICC8	ZXDA	Silent	chrX	57935700	57935700	G	A	ZXDA:NM_007156:exon1:c.C1155T:p.P385P

A total of 465 somatic variants of point mutations and indels are listed. Category and Description entries are as available from ANNOVAR output. Genomic coordinates (Chr-Start-End) are shown in accordance with hg19.

Supplementary Table S5: Sequencing depth and coverage of RNA-seq

Case	Total reads	Processed reads	Mapped reads (%)*
ICC3N	39,240,095	39,236,707	26,982,502 (68.77%)
ICC3T	41,584,465	41,581,871	28,075,932 (67.52%)
ICC4N	44,541,559	44,541,334	31,819,783 (71.44%)
ICC4T	42,314,860	42,314,671	28,613,455 (67.62%)
ICC5N	32,569,502	32,552,253	22,324,087 (68.58%)
ICC5T	38,858,041	38,837,045	26,598,910 (68.49%)
ICC6N	36,386,404	36,377,905	22,592,879 (62.11%)
ICC6T	35,751,940	35,743,586	23,714,643 (66.35%)
ICC8N	34,882,106	34,871,053	22,627,403 (64.89%)
ICC8T	38,533,764	38,521,049	26,017,949 (67.54%)
ICC10N	44,865,248	44,852,867	31,793,832 (70.88%)
ICC10T	40,379,483	40,367,741	29,332,807 (72.66%)
ICC11N	40,352,840	40,330,076	30,500,014 (75.63%)
ICC11T	33,770,469	33,751,129	24,071,853 (71.32%)
ICC14N	39,338,726	39,336,507	28,385,655 (72.16%)
ICC14T	38,969,644	38,967,440	26,276,632 (67.43%)
ICC15N	38,883,855	38,875,449	28,791,362 (74.06%)
ICC15T	45,572,720	45,563,058	33,915,385 (74.44%)
ICC16N	74,339,468	74,337,560	54,423,105 (73.21%)
ICC16T	77,824,196	77,822,242	54,973,243 (70.64%)

*Mapping rate is calculated based on TopHat output.

Supplementary Table S6: Pre- and post-operational treatment for ICC patients

	Pre-operative		Post-operative	
Case	Chemotherapy	Radiotherapy	Chemotherapy	Radiotherapy
ICC3	No	No	No	No
ICC4	No	No	No	No
ICC5	No	No	No	No
ICC6	No	No	No	No
ICC8	No	No	**Yes (cisplatin, gemcitabine)**	**Yes (4500cGy)**
ICC10	**Yes (gemcitabie, capecitabine, oxaliplatin)**	No	No	No
ICC11	**Yes (cisplatin, gemcitabine)**		**Yes (cisplatin, gemcitabine)**	No
ICC14	No	No	No	No
ICC15	No	No	No	No
ICC16	No	No	**Yes (cisplatin, gemcitabine)**	No
ICC19	No	No	**Yes (sorafenib tosylate)**	No
ICC23	No	No	No	No
ICC25	No	No	No	No
ICC26	No	No	**Yes (gemcitabine)**	No
ICC29	No	No	No	No
ICC30	No	No	No	No
ICC31	No	No	No	No

The drug regimen and radiation dose are shown if the patient has received chemotherapy or radiotherapy before or after the surgery.
